# DMGV Is a Rheostat of T Cell Survival and a Potential Therapeutic for Inflammatory Diseases and Cancers

**DOI:** 10.3389/fimmu.2022.918241

**Published:** 2022-08-05

**Authors:** Fengyuan Mandy Yang, Liya Shen, Dengxia Denise Fan, Kuan-Hung Chen, Jongdae Lee

**Affiliations:** ^1^ School of Basic Medical Sciences, Guangdong Provincial Key Laboratory of Allergy & Clinical Immunology, and the State Key Laboratory of Respiratory Diseases, Guangzhou Medical University, Guangzhou, China; ^2^ Department of Orthopedics, The 1st Affiliated Hospital of Sun Yat-sen University, Guangdong, China

**Keywords:** DMGV, ADMA, SDMA, AGXT2, MCU, NLCX

## Abstract

Activated effector T cells (Teff) and/or compromised regulatory T cells (Treg) underlie many chronic inflammatory diseases. We discovered a novel pathway to regulate survival and expansion of Teff without compromising Treg survival and a potential therapeutic to treat these diseases. We found dimethylguanidino valeric acid (DMGV) as a rheostat for Teff survival: while cell-intrinsic DMGV generated by Alanine-Glyoxylate Aminotransferase 2 (AGXT2) is essential for survival and expansion by inducing mitochondrial ROS and regulation of glycolysis, an excessive (or exogenous) DMGV level inhibits activated Teff survival, thereby the AGXT2-DMGV-ROS axis functioning as a switch to turn on and off Teff expansion. DMGV-induced ROS is essential for glycolysis in Teff, and paradoxically DMGV induces ROS only when glycolysis is active. Mechanistically, DMGV rapidly activates mitochondrial calcium uniporter (MCU), causing a surge in mitochondrial Ca^2+^ without provoking calcium influx to the cytosol. The mitochondrial Ca^2+^ surge in turn triggers the mitochondrial Na^+^/Ca^2+^ exchanger (NCLX) and the subsequent mitochondrial Na^+^ import induces ROS by uncoupling the Coenzyme Q cycle in Complex III of the electron transport chain. In preclinical studies, DMGV administration significantly diminished the number of inflammatory T cells, effectively suppressing chronic inflammation in mouse models of colitis and rheumatoid arthritis. DMGV also suppressed expansion of cancer cells *in vitro* and in a mouse T cell leukemic model by the same mechanism. Our data provide a new pathway regulating T cell survival and a novel mode to treat autoimmune diseases and cancers.

## Introduction

Although biologics such as TNF antagonists revolutionized the treatments of chronic inflammatory diseases including inflammatory bowel disease (IBD) and rheumatoid arthritis (RA), a large portion of patients do not respond or develop resistance to these drugs (1), necessitating alternative medicines to treat them. A meta-analysis shows that 1/3 of the IBD patients do not respond to anti-TNF treatment, and more than 1/3 patients receiving TNF antagonists experience a loss of response or intolerance to treatment (1). Similarly for RA, only 25–42% of patients reach the treatment target of ACR50 (American College of Rheumatology Score 50) (2). Teff, especially Th1 and Th17, are the main drivers of inflammatory bowel disease (IBD) and RA when the balance between Teff and Treg is perturbed (3, 4). For example, Treg cells from RA patients were found functionally compromised (5, 6) most likely due to the inflammatory environment in which they are localized (7). Therefore, disabling Teff without affecting Treg functions may effectively treat these diseases. Naïve CD4^+^ T cells differentiate into different Teff or Treg influenced by the microenvironmental milieu including cytokines (8). The metabolic reprograming determines the fate of Teff and memory T cells (T_M_), which utilize distinct metabolic pathways (9). While Teff use aerobic glycolysis as the main fuel for their functions, Treg use oxidative phosphorylation (10). However, a low glucose metabolism is sufficient for IL-2 production, and the mitochondrial ROS (reactive oxygen species) is required activation of nuclear factor of activated T cells (NFAT) and subsequent IL-2 induction (11).

Methylated arginine derivates are generated in the cells by protein arginine methyltransferases (PRMTs) and liberated when proteins containing them are recycled. PRMTs regulate a variety of cellular functions such as transcription, splicing, RNA biology, the DNA damage response and cell metabolism; these fundamental processes are altered in many diseases and thus PRMTs are therapeutic targets (12). Asymmetric dimethylarginine (ADMA) and its enantiomer symmetric dimethylarginine (SDMA) are generated by PRMT I and PRMT II enzymes, respectively (13). ADMA is a natural inhibitor of nitric oxide synthase (NOS) and thus implicated in cardiovascular diseases and others (13). However, SDMA has no direct effect on NOS (14). ADMA and SDMA are further metabolized to L-citrulline by dimethylarginine dimethylaminohydrolases (DDAHs) in the cytosol or to DMGV by AGXT2 in the mitochondria (15). While anti-cyclic citrullinated peptides (anti-CCPs) are used as one of the biomarkers for rheumatoid arthritis (16), DMGV was identified as a marker of liver fat and predictor of diabetes (17).

In this report, we identified DMGV as an important regulator of Teff survival, but not Treg, *via* induction of mitochondrial ROS, which makes it an ideal drug candidate to control T cell-mediated diseases. As DMGV regulates Teff survival through the control of glycolysis, it also limits survival of cancer cells depending on Warburg effect for proliferation. Our findings provide a novel approach to treat chronic inflammatory diseases and cancers.

## Results

### ADMA and SDMA Inhibit T Cell Proliferation

CD4^+^ effector T cells (Teff) are the major drivers of chronic inflammatory or autoimmune diseases including inflammatory bowel disease (IBD) and rheumatoid arthritis (RA) (3, 4). As immunometabolites regulate the functions of different immune cells (18–20), we embarked to discover novel small immunometabolites from non-immune cells that limit expansion of T cells. We screened several cell lines to discover soluble factors in the conditioned medium (CM) that regulate proliferation of mouse CD3^+^ T cells. We used live cell gating in flow cytometry for measuring cell death and survival since it was mostly consistent with Annexin V/PI staining (**Supplementary Figure 1A**). The mouse embryonic fibroblasts (MEF) CM completely abrogated the proliferation of T cells whereas the CM from the human epithelial cell line 16HBE (and other epithelial cell lines, data not shown) generally promoted it (**Supplementary Figure 1B**). To identify small molecules that inhibit T cell proliferation, we partially purified the MEF CM components smaller than 3kiliodaltons (3KD) (**Supplementary Figure 1C** left) and the inhibitory factor (named as LNY1) was found in fractions 4 and 5 (F4, F5) (**Supplementary Figure 1C**, right). LNY1 was further purified by HPLC (**Supplementary Figure 1D**) and the purified LNY1 inhibited T cell survival and proliferation (**Supplementary Figure 1E**) with the mass/charge (m/z) ratio of 203 (**Supplementary Figure 1F**). Two compounds with the same m/z (mass/charge) ratio that were reported to be produced by MEF are myo-inositol and asymmetric dimethylarginine (ADMA) (21). We focused on ADMA since myo-inositol can be converted to myo-inositol 1,4,5-trisphosphate (IP3), which activates calcium mobilization, a key T cell activation pathway. Protein arginine methyltransferase 1 (PRMT1) is the primary enzyme for ADMA biosynthesis while PRMT5 is essential for the generation of symmetric dimethylarginine (SDMA), an ADMA enantiomer (22). To test whether LNY1 is ADMA and/or SDMA, we collected CM after silencing PRMT1 and/or PRMT5 in MEF. The MEF CM after PMRT1 or PMRT5 knockdown (KD) inhibited T cell proliferation significantly less than the control CM did while the CM from PMRT1/5 double KD almost completely lost the ability to inhibit proliferation (**Supplementary Figure 1G**). Importantly, both ADMA and SDMA inhibited T cell proliferation (**Supplementary Figure 1H**). LNY1, ADMA, or SDMA also inhibited proliferation of mouse splenic CD19^+^ B cells (**Supplementary Figure 1I**) and human peripheral CD3^+^ T cells (**Supplementary Figure 1J**). LNY1 and ADMA also inhibited proliferation of pre-activated mouse and human T cells while rapamycin inhibited proliferation of activated human T cells only (**Supplementary Figure 1K**). Taken together, we discovered that ADMA and SDMA inhibit expansion of lymphocytes.

### ADMA and SDMA Inhibit T Cell Proliferation *via* Induction of Mitochondrial ROS

**Figure 1:**
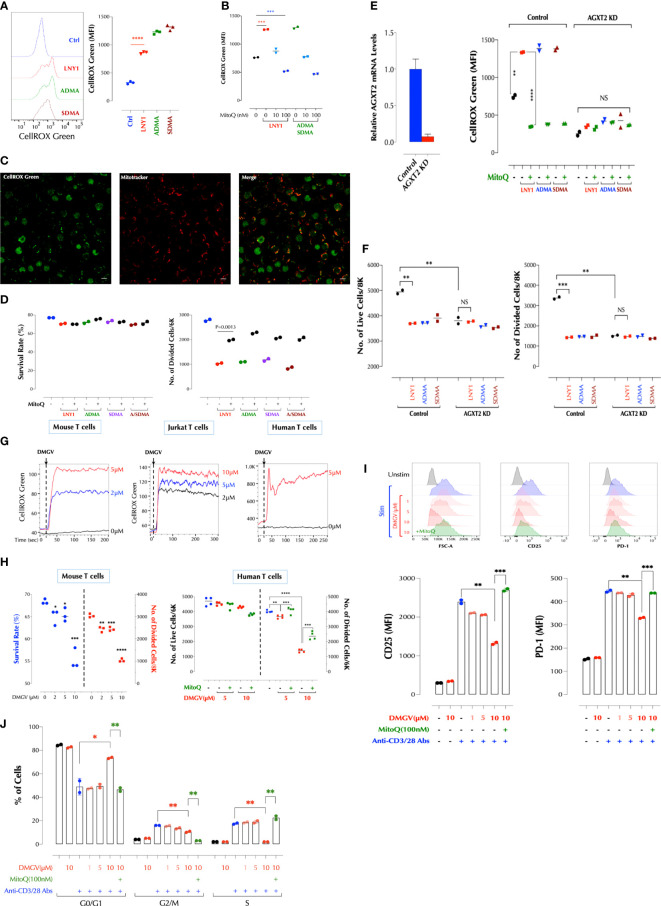
DMGV, a metabolite of ADMA and SDMA, inhibits T cell proliferation *via* induction of mitochondrial ROS. **(A)** ADMA and SDMA induces ROS in the mitochondria. Mouse splenic T cells were incubated with LNY (100ng/ml), ADMA (10µM), or SDMA (10µM) for 30min and ROS was measured by flow cytometry. (n=3 independent biological samples, ***<0.001) **(B)** ROS induction by ADMA and SDMA is inhibited by MitoQ. Mouse splenic T cells were incubated for 30min with LNY1 (100ng/ml) or ADMA + SDMA (10µM) with or without MitoQ as indicated and ROS was measured by flow cytometry. (n=2 independent biological samples, ***<0.001) **(C)** ADMA induces ROS in T cells. Freshly isolated mouse splenic T cells were incubated with ADMA (10µM), MitoTracker Deep Red, and CellROX Green for 30min and confocal live cell imaging was performed. **(D)** ADMA and SDMA inhibit T cell proliferation *via* induction of mitochondrial ROS. Mouse splenic T cells were stimulated with or without LNY1 (100ng/ml), ADMA (10µM), SDMA (10µM), or ADMA/SDMA (A/SDMA) and survival and proliferation were measured at 48h as described in the method. (n=2 independent biological samples). MitoQ (100nM) was added at the same time as indicated. **(E)** AGXT2 is essential for ADMA or SDMA to induce mitochondrial ROS. AGXT2 in mouse splenic CD3^+^ T cells was silenced using siRNA, and knockdown (KD) was confirmed by qPCR (left). Mitochondrial ROS induction by the indicated compounds in control or AGXT2 KD cells was measured with or without pre-incubation for 30min with MitoQ (100nM). (n=2 independent biological samples; NS, not significant; **<0.01, ****<0.0001) **(F)** AGXT2 is essential for ADMA or SDMA to inhibit T cell survival and proliferation. Survival and proliferation of control and AGXT2 KD mouse splenic CD3^+^ T cells, incubated with LNY1 (100ng/ml) or ADMA (10μM) alone, or together with MitoQ (100nM), were measured by flow cytometry. (n=2 independent biological samples, NS, not significant; **<0.01, ****<0.0001) **(G)** DMGV induces ROS in mouse, Jurkat, and human T cells in a dose-dependent manner. The indicated amount of DMGV was pulsed in at 20 seconds into CellROX Green-loaded cells and ROS was measured by time-lapse flow cytometry. **(H)** DMGV inhibits T cell survival and expansion *via* mitochondrial ROS induction. Mouse splenic CD3^+^ T cells (left) or human peripheral blood CD3^+^ T cells (right) were activated in the presence of DMGV alone or DMGV with MitoQ (100nM), and survival and proliferation were measured by flow cytometry. (3-4 biological samples, **<0.01, ***<0.001, ****<0.0001). All above data were repeated at least twice in similar settings with similar results. **(I)** DMGV inhibits T cell activation *via* ROS generation. Primary human T cells were activated in the presence of the indicated compounds for 24h, and the cell size (FSC-A) and the expression of CD25 and PD-1 was measured by flow cytometry. **(J)** DMGV inhibits cell cycle progression. Primary human T cells were activated in the presence of the indicated compounds for 48h, and the cell cycle was measured by flow cytometry. All the above data were repeated at least twice with the similar results. *<0.05.

In order to gain insights on how LNY1 (ADMA and SDMA) inhibits T cell proliferation, RNA sequencing was performed after mouse splenic T cells were stimulated with or without LNY1 for 24h, at which time cells were undivided and LNY1-induced cell death was minimal. LNY1 significantly changed the TCR-induced gene expression pattern (**Supplementary Figure 2A**, left), affecting several metabolic pathways. While the genes regulating cellular metabolisms (fatty acids, amino acids, glucose) and cell cycle pathways were down-regulated by LNY1, those related to neurogenerative diseases, oxidative phosphorylation, and chemokine/cytokine signaling pathways were up-regulated (**Supplementary Figure 2A**, right). The genes upregulated in the neurogenerative diseases and oxidative phosphorylation pathways were overlapping and the components of the mitochondrial electron transport chain, such as Cox5a, Atp5g3, Ndufa5, and Uqcr10. ADMA was reported to increase NOS (nitric oxide synthase)-derived ROS in endothelial cells in the absence of the NOS cofactor tetrahydrobiopterin (23). We, therefore, investigated whether LNY1 (ADMA/SDMA) regulates the mitochondrial functions. Overnight stimulation of T cells in the presence of LNY1 significantly decreased the TCR-induced-mitochondrial membrane potential (ΔΨm, TMRE) (**Supplementary Figure 2B**) but increased the ROS level (**Supplementary Figure 2C**). LNY1 directly induced ROS in the mitochondria measured by the mitochondria-specific ROS indicator CellROX Green but not in the cytoplasm measured by the cytoplasm-specific CellROX Deep Red (**Supplementary Figure 2D** left) while tBHP (tert-Butyl hydroperoxide) rapidly accumulated in the cytosol (**Supplementary Figure 2D** right). LNY1 enhanced TCR-induced mitochondrial ROS at various time points after TCR stimulation (**Supplementary Figure 2E**). Consistently, ADMA- or SDMA-induced mitochondrial ROS (**Figure 1A**) was abrogated by the mitochondria-directed antioxidant mitoquinone mesylate (MitoQ) (**Figure 1B**). ADMA- and SDMA-induced ROS in live splenic T cells were detected also by live-cell confocal microscopy (**Figure 1C** and **Supplementary Figure 2F**). Next, we tested whether accumulation of excessive mitochondrial ROS is responsible for ADMA/SDMA-induced inhibition of proliferation. Indeed, MitoQ rescued T cells from ADMA- or SDMA-mediated inhibition in proliferation (**Figure 1D**). The antioxidants glutathione (GSH) or N-acetylcysteine (NAC) also reversed the inhibition by ADMA and/or SDMA (data not shown), but these antioxidants by themselves had no significant effect on T cell proliferation (**Supplementary Figure 2G**). As ADMA and SDMA were reported to inhibit the activity of nitric oxide synthase (NOS) and thus decrease the NO level (24, 25), we tested whether the iNOS (inducible NOS) inhibitor N6-(1-iminoethyl)-L-lysine (NIL) inhibits T cell proliferation. NIL even at a high concentration did not significantly affect T cell proliferation (**Supplementary Figure 2G**) or induce ROS (**Supplementary Figure 2H**). Consistently, more CD4 and CD8 memory T cells were found in mice deficient in iNOS than in WT mice after immunization without an apparent defect in T cell proliferation (26). Together, these data demonstrate that both ADMA and SDMA inhibit proliferation of lymphocytes by inducing mitochondrial ROS.

### DMGV-Induced Mitochondrial ROS Functions as a Rheostat of T Cell Survival and Expansion

**Figure 2:**
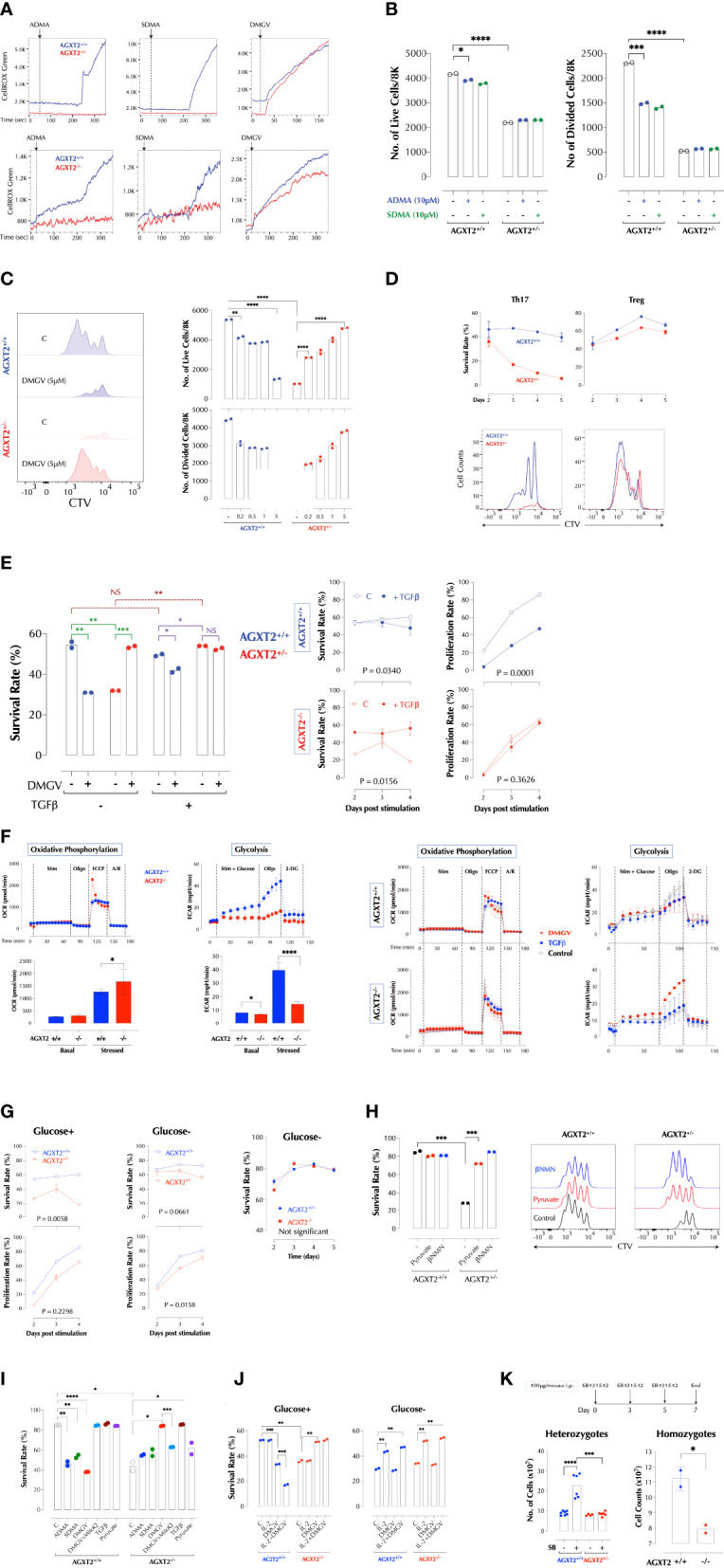
DMGV-induced mitochondrial ROS is a rheostat of T cell survival and expansion. **(A)** ADMA and SDMA induces mitochondrial ROS *via* AGXT2. ROS induction by 5µM of ADMA, SDMA, or DMGV in splenic T cells from WT and AGXT2^+/-^ mice (Upper panel) and AGXT2^-/-^ mice (Lower panel) was measured by time-lapse flow cytometry and graphed using the FlowJo kinetics. The data are the representatives of 3 independent experiments. **(B)** ADMA and SDMA inhibit T cell proliferation *via* AGXT2. CTV-labeled splenic T cells from WT and AGXT2^+/-^ mice were activated with or without 5µM of ADMA, SDMA, or DMGV for 2 days and survival and proliferation were measured by flow cytometry. **(C)** DMGV is a rheostat of T cell survival. CTV-labeled splenic T cells from WT and AGXT2^+/-^ mice were activated with the indicated amount of DMGV for 3 days and survival and proliferation were measured by flow cytometry. **(D)** DMGV-induced mitochondrial ROS controls survival of Th17 T cells, but not Treg. CTV-labeled splenic T cells from WT and AGXT2^+/-^ mice were activated as indicated and the survival rate was measured by flow cytometry. The data are the representatives of 2 independent experiments. **(E)** TGFβ and DMGV redundantly support survival of mouse T cells during expansion. Splenic CD3^+^ T cells from WT and AGXT2^+/-^ mice were activated with DMGV (5µM) and/or TGFβ (15ng/ml) for 3 days and survival was measured by flow cytometry. **(F)** Glycolysis is compromised in AGXT2^-/-^ T cells. Splenic WT and AGXT2^+/-^ T cells were stimulated with or without DMGV (5µM) or TGFβ (15ng/ml), and OxPhos and glycolysis potentials were measured by Seahorse mitostress and glycolysis assay. (Left) Glycolysis, but not OxPhos, is significantly compromised in AGXT2^-/-^ T cells. (Right) DMGV, but not TGFβ, rescues glycolysis in AGXT2^-/-^ T cells. **(G)** AGXT2 regulates glycolysis to support T cells survival and expansion. CTV-labeled splenic CD3^+^ T cells from WT and AGXT2^+/-^ mice (Left and middle panels) or AGXT2^-/-^ mice (Right panel) were activated in glucose-containing or glucose-free RPMI for 4-5 days and survival and proliferation were measured by flow cytometry. **(H)** The glycolysis metabolite pyruvate or βNMN rescues AGXT2^+/-^ T cells. CTV-labeled splenic CD3^+^ T cells from WT and AGXT2^+/-^ mice were activated with or without pyruvate (1mM) or βNMN (15µM) for 3 days and survival and expansion were measured by flow cytometry. **(I)** AGXT2^-/-^ T cells phenocopy AGXT2^+/-^ T cells. CTV-labeled splenic CD3^+^ T cells from WT and AGXT2^+/-^ mice were activated as indicated for 4 days and survival and expansion were measured by flow cytometry. **(J)** DMGV no longer affects T cell survival in a glucose-free medium. CTV-labeled splenic CD3^+^ T cells from WT and AGXT2^-/-^ mice were activated as indicated for 4 days either in glucose+ or glucose- RPMI and survival and expansion were measured by flow cytometry. **(K)** AGXT2 is necessary for expansion of splenocytes in the absence of TGFβ signaling *in vivo*. from WT and AGXT2^+/-^ mice (left) were injected i.p. with SB431542 (400µg/mouse) in 5% DMSO/PBS on days 0, 3, and 5 and the number of splenocytes on day 7 were counted manually with a hemocytometer. The data are the combined results of two separate experiments. WT and AGXT2^-/-^ mice (right) were injected i.p. with SB431542 (400µg/mouse) in 5% DMSO in PBS on days 0 and 3 and the number of splenocytes on day 5 were counted. *<0.05; **0.01; ***0.001; ****<0.001, NS, not significant.

ADMA and SDMA are metabolized by dimethylarginine dimethylaminohydrolases (DDAHs) to L-citrulline in the cytosols or to DMGV (dimethylguanidino valeric acid) by alanine-glyoxylate aminotransferase 2 (AGXT2) in the mitochondria (27, 28) (**Supplementary Figure 3A**). L-citrulline can be converted back to L-arginine, with which different isoforms of nitric oxide synthase (NOS) synthesize NO (29). L-citrulline neither induced ROS nor significantly inhibited T cell survival and proliferation while DMGV rapidly induced ROS (**Supplementary Figure 3B**). However, AGXT2 knockdown (KD) abrogated the ability of ADMA or SDMA to induce ROS (**Figure 1E**) or inhibit T cell survival and proliferation (**Figure 1F**). Unexpectedly, AGXT2 KD itself significantly compromised T cell survival (**Figure 1F**), suggesting that AGXT2 and thus the **intrinsic** DMGV-induced mitochondrial ROS is necessary for the optimal survival and expansion of T cells. DMGV rapidly induced ROS in mouse and human T cells as well as Jurkat T cells (**Figure 1G**), and inhibited cell survival and proliferation *via* mitochondrial ROS induction in mouse and human T cells (**Figure 1H**). DMGV *via* ROS inhibited the initial growth and activation of T cells as indicated by the smaller cell size and reduced expression of CD25 and PD-1 in the cells treated with DMGV for 24h (**Figure 1I**). DMGV blocked cell cycle progression *via* generation of ROS (**Figure 1J**). Overexpression (OE) of AGXT2 in T cells significantly increased mitochondrial ROS induced by ADMA or SDMA (**Supplementary Figure 3C**), and AGXT2 OE itself inhibited survival and expansion of T cells (**Supplementary Figure 3D**). However, ADMA and SDMA no longer had an impact on proliferation in the AGXT2-OE cells (**Supplementary Figure 3D**), suggesting that enough cell-intrinsic ADMA and/or SDMA are present in the AGXT2-OE cells to be converted to DMGV. AGXT2 was localized in the mitochondria of the human epithelial cell line HCT-8 (**Supplementary Figure 3E**). DMGV induced mitochondrial ROS in all types of human PBMCs within 1h (**Supplementary Figure 3F** top), which diffused to the cytosol in 24h (**Supplementary Figure 3F** bottom), most likely due to the compromised mitochondrial function. In contrast, tBHP (tert-butyl hydroperoxide) added to the medium was initially detected in the cytosol and then diffused to the mitochondria later. In the absence of TCR stimulation, however, DMGV did not significantly affect the viability of human PBMCs within 24h whereas tBHP killed both T and B cells (**Supplementary Figure 3G**). DMGV also caused ROS-mediated death in activated Jurkat T cells in 24h (**Supplementary Figure 3H**). ADMA, SDMA, and DMGV blocked entry to GO-G1 phase in the cell cycle (**Supplementary Figure 3I**) consistent with the data on human T cells (**Figure 1J**). In order to determine the *in vivo* roles of AGXT2, we generated AGXT2-deficient mice (C57BL/6 background). A few homozygotes were obtained up to 4 generations (**Supplementary Figures 4A, B**). Because the AGXT2^+/-^ T cells were hypomorphs expressing a very low level of AGXT2 compared to WT (**Supplementary Figures 4C, D**), the experiments were initially performed with the heterozygotes but later most of the important experiments were replicated with the homozygotes. No abnormality in T cell development was evident in the heterozygotes or homozygotes (**Supplementary Figure 4E**). Consistent with the AGXT2 KD results, ADMA and SDMA no longer induced ROS in AGXT2^+/-^ or AGXT2^-/-^ T cells while DMGV did; and ADMA and SDMA in WT T cells induced ROS at a considerably slower pace than DMGV did (**Figure 2A**), most likely reflecting the time for them to be metabolized to DMGV. ADMA and SDMA no longer inhibited T cell survival or proliferation in the heterozygotes (**Figure 2B**). Consistent with AGXT2-KD cells, survival and proliferation AGXT2^+/-^ T cells were severely compromised (**Figure 2B**). In addition, the AGXT2 mRNA was induced during T cell activation (**Supplementary Figure 4F**). These data indicate that a threshold and increasing level of AGXT2-DMGV-ROS is necessary for T cells to survive and expand while the AGXT2-DMGV-ROS level below or above the threshold is detrimental to T cells, establishing the AGXT2-DMGV-ROS axis as a rheostat of T cell survival (30). Indeed, while DMGV inhibited survival and proliferation in WT cells, it rescued AGXT2^+/-^ T cells from death in a dose-dependent manner (**Figure 2C**) *via* induction of mitochondrial ROS (**Supplementary Figure 4G**). A similar result was obtained in human T cells (**Supplementary Figure 4H**). We next tested if AGXT2 is necessary for survival of different CD4^+^ Teff. While naïve CD4^+^ AGXT2^+/-^ T cells under the Th1, 2, or 17 induction condition did not survive well, they survived similarly to the WT cells under the Treg induction condition (**Supplementary Figure 4I**). From the difference between Th17 (IL-6 + a low-dose TGFβ) and Treg induction condition (a high-dose TGFβ), we hypothesized that TGFβ protects AGXT2^+/-^ T cells from death during expansion and IL-6 negates the protection provided by TGFβ. Indeed, survival and division of naïve CD4^+^ AGXT2^+/-^ T cells were little affected under the Treg induction condition while they gradually died off under the Th17 induction condition (**Figure 2D**). Furthermore, TGFβ rescued total CD3^+^ AGXT2^+/-^ T cells (non-induction condition) while it slightly inhibited the survival of WT T cells (**Figure 2E**). It is thought that T cells preferentially use oxidative phosphorylation (OxPhos) in the initial phase of activation and fatty acid oxidation (FAO) but later switch to glycolysis and glutaminolysis to meet the energy requirement as well as generate building blocks necessary for the biosynthetic needs. As Foxp3 in Tregs promotes OxPhos but inhibits glycolysis (31), we suspected that AGXT2^+/-^ T cells have a defect in glycolysis. While there was no difference at the basal OxPhos level, OxPhos in AGXT2^-/-^ T cells under the stressed condition was slightly but significantly elevated compared to WT cells. On the other hand, glycolysis in AGXT2^-/-^ T cells was significantly compromised under both the basal and stressed conditions (**Figure 2F**, left panel). DMGV had no significant impact on OxPhos; DMGV slightly inhibited glycolysis in WT but restored it in AGXT2^-/-^ T cells (**Figure 2F**, right panel), consistent with the survival data (**Figure 2C**). However, TGFβ did not influence OxPhos but suppressed glycolysis slightly in WT without rescuing it in AGXT2^-/-^ T cells (**Figure 2F**, lower panel), indicating that TGFβ rescues AGXT2^-/-^ T cells by a different mechanism than DMGV. We next tried to rescue AGXT2^+/-^ T cells by forcing them to utilize OxPhos mostly in a glucose-free RPMI medium (with glucose provided only by 10% FBS) or supplementing them with the glycolysis intermediates. There was little difference in survival between WT and AGXT2^+/-^ or AGXT2^-/-^ T cells in glucose-free RPMI (**Figure 2G**), and in glucose+ medium, supplementation of either pyruvate or β-nicotinamide mononucleotide (βNMN) prevented death of AGXT2^+/-^ T cells during expansion while they had little effect on WT T cells (**Figure 2H**). Most of these results were reproduced in AGXT2^-/-^ T cells (**Figure 2I**). Unexpectedly, DMGV had no effect at all on T cell survival in a glucose-free medium, and IL-2 had no effect on AGXT2^-/-^ T cells with glucose but promoted survival of both WT and AGXT2^-/-^ T cells in the absence of glucose (**Figure 2J**). These data indicate that the major metabolic defect in AGXT2^+/-^ T cells lies in glycolysis rather than OxPhos and thus are unable to make the metabolic switch. These data were replicated in Jurkat T cells; AGXT2 KD significantly decreased the cell survival, which was reversed by DMGV or TGFβ (**Supplementary Figure 4J**). As glutaminolysis is another important metabolism for T cells, we performed a similar experiment in glutamine-free RPMI medium. DMGV had a less impact on WT survival but rescued AGXT2^-/-^ T cell survival (but not expansion) in a glutamine-free medium; however, TGFβ was not able to rescue AGXT2^-/-^ T cells (**Supplementary Figure 4K**). Thus, TGFβ rescues AGXT2^-/-^ T cells in a glutaminolysis-dependent mechanism. Since there was no difference in T cell development and numbers in the thymus and spleen between AGXT2^+/-^ and WT mice (**Supplementary Figure 4E**), we hypothesized that TGFβ signaling provides an alternative mechanism for AGXT2-deficient T cells to survive *in vivo*. TGFβ is not only crucial for Treg development but also restrains proliferation of self-reactive CD4^+^ and CD8^+^ T cells (32). Therefore, inhibition of TGFβ signaling in T cells causes autoimmunity similar to that in Treg-deficient mice (33) and TGFβ1 deficiency in humans causes severe inflammatory bowel disease and encephalopathy (34). To test if TGFβ provides a survival signaling to AGXT2^+/-^ or AGXT2^-/-^ T cells *in vivo*, we injected the TGFβ receptor inhibitor SB431542 to WT, AGXT2^+/-^ and AGXT2^-/-^ mice and measured the effects on T cell survival. As expected, the number of splenocytes increased significantly in WT mice, but unchanged in AGXT2^+/-^ or AGXT2^-/-^ mice (**Figure 2K**), indicating that AGXT2 is essential for lymphocyte expansion in the absence of TGFβ signaling. The T to B cell ratio in the spleens did not change after the SB431542 treatment (data not shown). Taken together, these data demonstrated that the intrinsic threshold level of DMGV-induced mitochondrial ROS dictated by the AGXT2 expression level during Teff expansion is essential for glycolysis and survival of T cells (in the absence of TGFβ signaling) whereas excessive DMGV compromises their survival. Glucose uptake wasonly slightly decreased in AGXT2^+/-^ T cells before and after TCR stimulation (**Supplementary Figure 4L**). Therefore, how the DMGV-ROS axis regulates glycolysis needs to be further investigated.

### DMGV Activates Mitochondrial Calcium Uniporter (MCU) to Induce ROS

We next investigated how DMGV induces ROS in the mitochondria. In Jurkat T cells, DMGV-induced ROS could be measured with two different mitochondrial-specific ROS indicators, MitoSOX and CellROX Green (**Supplementary Figure 5A**). CellROX Green was chosen because of the more robust signal. MitoQ abolished DMGV-induced ROS whereas tBHP was not immediately detectable by CellROX Green (**Supplementary Figure 5B**) although it can be detected over time (**Supplementary Figures 2D, 3F**). As DMGV also induces cell death in activated Jurkat cells *via* ROS (**Supplementary Figure 3H**), we used Jurkat cells as a model system to study the mechanism. DMGV induced ROS also in a cell-free system with the intact mitochondrial membrane potential in digitonin-permeabilized Jurkat cells (**Supplementary Figure 5C**) or mouse T cells (**Supplementary Figure 5D**).

**Figure 3:**
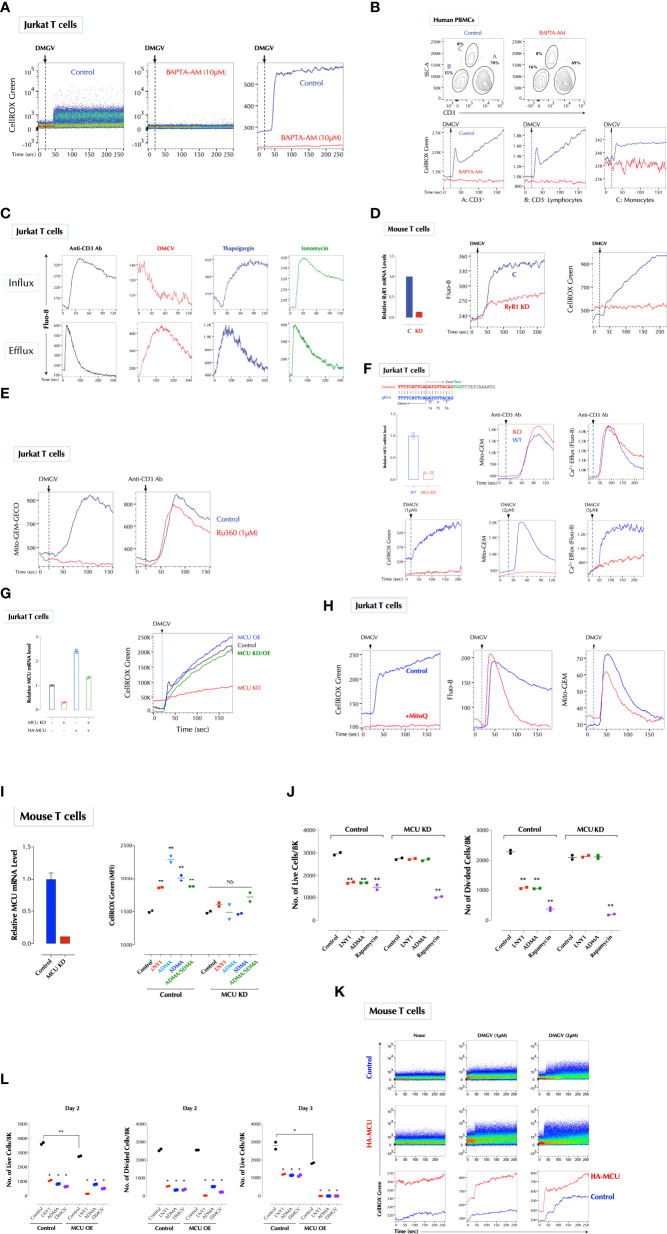
DMGV activates mitochondrial calcium uniporter (MCU) to induce ROS. **(A)** Calcium is essential for ROS induction by DMGV. Jurkat cells were loaded with CellROX Green with or without BAPTA-AM (10µM) for 15min and ROS induction by DMGV (5µM) was measured by flow cytometry. **(B)** Freshly isolated human PBMCs were loaded with CellROX Green with or without BAPTA-AM (10µM) for 15min and ROS induction by DMGV (5µM) was measured by flow cytometry. **(C)** DMGV induces calcium efflux, but not influx. Jurkat cells were loaded with Fluo-8 for 30min. For calcium efflux assay, cells were washed and resuspended in Ca^2^/Mg^2+^-free PBS before the assay by flow cytometry. Calcium influx was performed in HHBS (1X Hank’s with 20mM HEPES Buffer, pH 7.0) containing 1.2mM Ca^2+^. Anti-CD3 Ab (5µg/ml), Thapsigargin (1µg/ml), ionomycin (500ng/ml), or DMGV (5µM) was added 20 seconds after the start of acquisition. **(D)** RyR1 mediates calcium efflux and ROS induction by DMGV. Control or RyR1-KD mouse splenic T cells were treated with DMGV (5µM) to measure calcium efflux and ROS. **(E)** DMGV induces calcium influx to the mitochondria *via* MCU. DMGV-induced, but not TCR-induced, calcium influx to the mitochondria is inhibited by Ru360. Jurkat cells were incubated with Ru360 (1µM) for 30min and the induction of the mitochondrial Ca^2+^ by DMGV (5µM) or Anti-CD3 Ab (5µg/ml) was measured. **(F)** DMGV no longer induces mitochondrial calcium influx, calcium efflux, or ROS after MCU KO. MCU was knocked out using the Crispr/Cas9 system (top) and confirmed by qPCR. Control and MUC-KO cells expressing Mito-GEM-GECO were treated with the indicated amount of DMGV or anti-CD3 Ab (5µg/ml) and the mitochondrial calcium level, calcium efflux, and ROS were measured. **(G)** Ectopic MCU expression in MCU-deficient Jurkat T cells restores the ability of DMGV to induce mitochondrial ROS. Jurkat cells were transfected with MCU siRNA and/or HA-MCU-WT as indicated, and ROS induction by DMGV (5µM) was measured as above. (OE: over-expression) **(H)** Calcium mobilization by DMGV precedes ROS induction. Jurkat cells were incubated with MitoQ (100nM) for 30min, and ROS induction, calcium efflux, and mitochondrial calcium influx were measured. **(I)** ADMA and SDMA no longer induce ROS in MCU-deficient mouse T cells. MCU in mouse splenic T cells was silenced by siRNA and ROS induction by LNY1 (100ng/ml), ADMA (10µM), SDMA (10µM), or ADMA+SDMA was measured after 30min incubation by flow cytometry. **(J)** SDMA or ADMA no longer inhibits T cell proliferation after MCU KD. Control or MCU-KD mouse T cells were stimulated with or without LNY1 (100ng/ml), ADMA (5µM), or rapamycin (100ng/ml) for 3 days. The number of live and divided cells were measured by flow cytometry. **(K)** MCU OE induces ROS. Mouse splenic T cells were transfected with a control or HA-MCU plasmid, and ROS induction with or without DMGV (5µM) was measured. **(L)** MCU OE induces death of activated T cells. Control and MCU-OE mouse T cells were stimulated for 3 days with or without LNY1 (100ng/ml), ADMA (5µM), or DMGV (5µM), and the number of live and divided cells were measure by flow cytometry. All above data were repeated at least twice in similar settings with similar results. *<0.05; **0.01, NS, not significant.

Calcium influx to the mitochondria can generate ROS by activating dehydrogenases in the TCA cycle such as isocitrate dehydrogenase, α-ketoglutarate dehydrogenase, and pyruvate dehydrogenase, providing NADH to oxidative phosphorylation during which superoxide anion is produced (35). Calcium was required for DMGV to induce ROS since the intracellular calcium chelator BAPTA-AM completely abolished DMGV-induced ROS in Jurkat cells (**Figure 3A**) and human PBMCs (**Figure 3B**). We next tested whether DMGV directly induces calcium mobilization using the calcium indicator dye Fluo-8. While it did not induce calcium influx to the cytosol (**Figure 3C**, top), it rapidly induced calcium efflux measured in Ca^2+^/Mg^2+^-free PBS (**Figure 3C**, bottom). In contrast, stimulation of T cell receptor (TCR) with anti-CD3 antibody, the inhibitor of the sarco/endoplasmic reticulum Ca^2+^ ATPase (SERCA) Thapsigargin, or the ionophore ionomycin induced both influx and efflux (**Figure 3C**). The ER, due to its close proximity, is the major source of calcium for the mitochondria (36, 37). Ryanodine receptors (RYRs) and IP3 receptor (ITP3Rs) are the two major calcium channels in the ER that release calcium in response to appropriate stimuli. The RyR1 inhibitor ryanodine inhibited DMGV-induced ROS production in Jurkat cells in dose-dependent manner (**Supplementary Figure 5E**). Consistently, RyR1 KD inhibited DMGV-induced ROS as well as calcium efflux in mouse T cells (**Figure 3D**). Similarly, RyR1 KO decreased DMGV-induced ROS as well as calcium efflux in Jurkat cells while ITP3R1 KO did to a much less extent (**Supplementary Figure 5F**), thus establishing RyR1 as the major ER calcium channel for the DMGV-induced efflux. RyR1-depleted mouse T cells were also compromised in survival, but unlike AGXT2-deficient T cells they could not be rescued by DMGV (**Supplementary Figure 5G**), consistent with data that DMGV requires the functional RyR1 to induce ROS.

We next tested whether DMGV induces calcium import into the mitochondria using the mitochondria-specific calcium reporter Mito-GEM-GECO (38). DMGV rapidly induced calcium influx to the mitochondria, which was abolished by Ru360, a specific MCU blocker (39) whereas TCR-induced calcium influx to the mitochondria was not affected by Ru360 (**Figure 3E**). Consistently, DMGV induced neither calcium influx to the mitochondria nor ROS in MCU-KO Jurkat cells while TCR stimulation still induced mitochondrial calcium in MCU-KO cells (**Figure 3F**). In addition, DMGV-induced, but not TCR-induced, calcium efflux was abolished in MCU KO (**Figure 3F**), indicating that both calcium efflux and ROS induction by DMGV are the consequences of MCU-dependent calcium import to the mitochondria. To further verify the role of MCU, we over-expressed (OE) MCU-WT in MCU-KD Jurkat cells, which lost the response to DMGV. MCU-OE restored the ability of DMGV to induce ROS in MCU-KD cells (**Figure 3G**). These data together demonstrate that DMGV induces calcium import to the mitochondria *via* MCU to induce ROS. While mitochondrial calcium induces ROS, excessive ROS can also induce calcium in the mitochondria in a feed-forward cycle (40–42). DMGV still induced calcium efflux from the ER and influx to the mitochondria even after ROS induction was abolished by MitoQ (**Figure 3H**), confirming that calcium mobilization to the mitochondria by DMGV precedes and is responsible for ROS generation. Next, we investigated the role of MCU in T cell proliferation. ADMA and/or SDMA did not induce ROS (**Figure 3I**) and no longer inhibited T cells proliferation while rapamycin still did in MCU-KD mouse T cells (**Figure 3J**). Conversely, MCU OE elevated the basal mitochondrial ROS level (**Figure 3K**) and compromised the cell survival after TCR stimulation, in which all ADMA- or DMGV-treated MCU-OE T cells died after on day 3 (**Figure 3L**). Together these data demonstrate that DMGV induces ROS *via* MCU-mediated calcium influx to the mitochondria to regulate survival and expansion of activated T cells.

### Ile127 Is Essential for DMGV to Activate MCU

**Figure 4:**
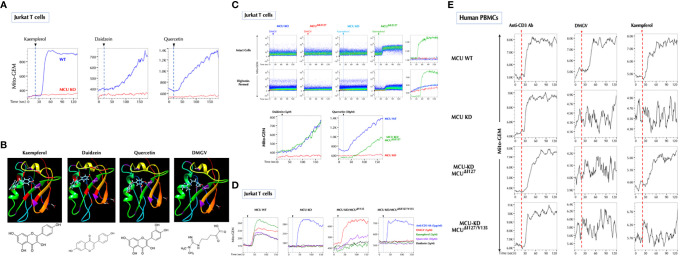
Ile127 is essential for DMGV to activate MCU. **(A)** The flavonoids induce calcium influx to the mitochondria *via* MCU. Control and MCU-KO Jurkat cells expressing Mito-GEM-GECO were treated with kaempferol (5µM), daidzein (5µM), or quercetin (50µM), and mitochondrial calcium flux (Mito-GEM) was measured by flow cytometry. **(B)** SwissDock predicts that the flavonoids and DMGV bind to MCU forming H-bonds to Ile127 and/or Val135. **(C)** DMGV no longer induces calcium through MCU^Δile127^ mutant. The MCU^Δile127^ mutant was transfected to MCU-KO Jurkat cells expressing Mito-GEM, and DMGV (5µM)-, kaempferol (5µM)-, daidzein (5µM), or quercetin (50µM)-induced mitochondrial calcium influx was measured. To measure the calcium flux in the cell-free system, the same set of cells were permeabilized with 50µM digitonin for 2min before the measurement. **(D)** Val135 is essential for the full activation by the flavonoids to induce calcium flux *via* MCU. MCU-KO Jurkat cells expressing Mito-GEM were transfected with MCU^Δval135^ or MCU^Δile127/Val135^ mutant, and DMGV (5µM)-, kaempferol (5µM)-, daidzein (5µM), or quercetin (50µM)-induced mitochondrial calcium influx was measured. Anti-CD3 Ab (5µg/ml) induced mitochondrial calcium influx independently of MCU. **(E)** Human PBMCs were infected with Lentiviruses expressing Mito-GEM-GECO and next day electroporated with control plasmid + control siRNA, control plasmid + MCU siRNA, MCU^Δile127^ plasmid + MCU siRNA, or MCU^Δile127/Val135^ plasmid + MCU siRNA. The following day, the cells were treated with anti-CD3 Ab (5µg/ml), DMGV (5µM), or kaempferol (5µM). All above data were repeated at least twice in similar settings with similar results.

Next, we investigated how DMGV induces the mitochondrial calcium flux *via* MCU. As previously reported (43), the flavonoids (kaempferol, daidzein and quercetin) induced mitochondrial calcium *via* MCU (**Figure 4A**), and also calcium efflux and mitochondrial ROS (data not shown). We tested whether the flavonoids can bind to MCU using the SwissDock program (44), and all 3 compounds were predicted to dock in the same pocket with different configurations. Kaempferol and daidzein were predicted to form two hydrogen bonds with MCU, one with Ile127 and another with Val135, but quercetin only with Ile127. Importantly, DMGV was also predicted to dock in the same pocket with a single hydrogen bond to Ile127 (**Figure 4B**). We predicted the carbonyl group in DMGV that forms a hydrogen bond to Ile127 is critical for its activity because the carbonyl group replaced the amino group in ADMA (**Supplementary Figure 6A**). The docking simulation data suggested that Ile127 and/or Val135 are the critical residues for these compounds to activate MCU. Expression of MCU^Δile127^ (Ile127 deletion) in MCU-KO cells completely restored the ability for kaempferol and daidzein to induce calcium or to a much less extent for quercetin, but not at all for DMGV (**Figure 4C**). These data indicate that Ile127 is essential for DMGV but that kaempferol and daidzein can still bind at Val135 to activate MCU. Indeed, DMGV and quercetin induced calcium *via* MCU^ΔVal135^, which significantly limited the induction by kaempferol and daidzein; neither the flavonoids nor DMGV could induce calcium to the mitochondria *via* MCU^Δile127/Val135^ (**Figure 4D**). The similar data were obtained when MCU^Δile127^ or MCU^Δile127/Val135^ was expressed in MCU-KD human PBMCs; while DMGV was completely dependent on Ile127, kaempferol could not induce calcium only when both Ile2127 and Val135 were deleted (**Figure 4E**). All recombinant MCU proteins were localized to the mitochondria (**Supplementary Figure 6B**).

### Na^+^ Import to the Mitochondria *via* NCLX Is Necessary for DMGV to Induce ROS

**Figure 5:**
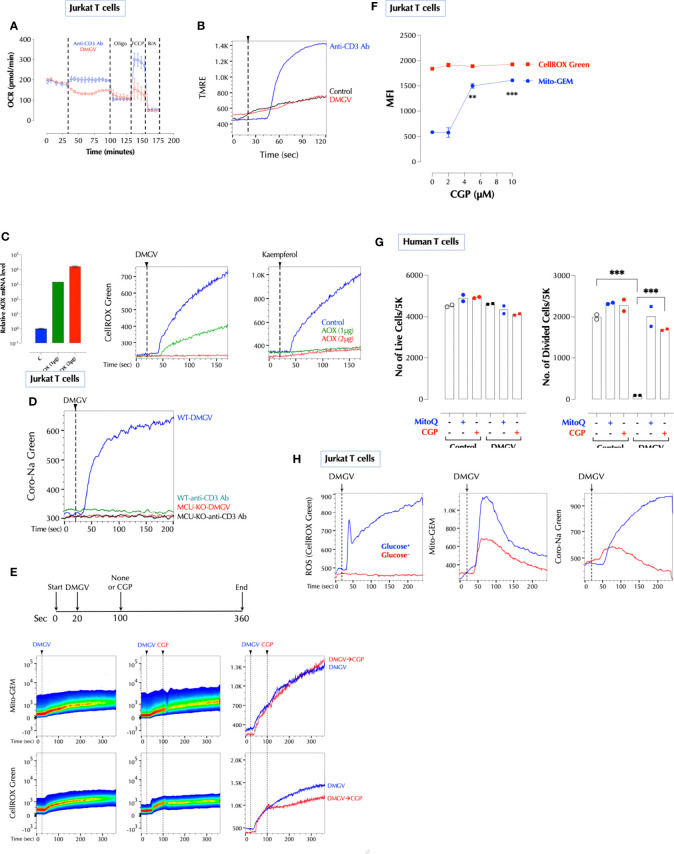
Na^+^ import to the mitochondria *via* NCLX is necessary for DMGV to induce ROS. **(A)** DMGV inhibits OxPhos in Jurkat cells. Seahorse assay was performed with DMGV (5µM) or anti-human CD3 Ab (5µg/ml) according to the manufacturer’s instruction. **(B)** DMGV does not increase the mitochondrial membrane potential. Jurkat cells loaded with TMRE were treated with none (control), DMGV (5µM) or anti-human CD3 Ab (5µg/ml). **(C)** Ectopic expression of alternative oxidase (AOX) inhibits ROS induction by DMGV. Jurkat cells were transfected with AOX, and ROS induction by DMGV (5µM) or kaempferol (5µM) was measured next day. **(D)** DMGV, but not TCR stimulation, induces Na^+^ influx to the mitochondria in an MCU-dependent manner. WT and MCU-KO Jurkat cells were loaded with Coro-Na Green and Na^+^ influx was measured by flow cytometry. **(E)** Induction of ROS, but not mitochondrial calcium influx, by DMGV is inhibited by the NCLX inhibitor CGP37157 (CGP, 10µM). Jurkat cells treated with DMGV (5µM) for 60 seconds followed by CGP (or none). **(F)** CGP37157 increases the mitochondrial calcium level without inducing ROS. Jurkat cells were incubated with the indicated amount of CGP37157 for 30min and Mito-GEM and CellROX Green were measured by flow cytometry (n = 2). **(G)** CGP prevents DMGV-induced inhibition of T cell proliferation. Primary human T cells were stimulated as indicated (5µM DMGV, 1µM CGP, 100nM MitoQ) and the number of live and divided cells were measured after 3 days by flow cytometry. **(H)** DMGV do not induce ROS in a glucose-free medium. Jurkat T cells incubated overnight in a glucose+ or glucose- RPMI, and mitochondrial ROS, calcium influx, and sodium influx were measured the next day as above. **<0.01; ***0.001.

Next, we investigated how DMGV/MCU-induced calcium generates ROS in the mitochondria. Initially, we thought that DMGV-induced calcium would activate TCA cycle to induce ROS (35). However, several data pointed out that this is not the case. Although TCR activation or ionomycin also rapidly mobilized calcium into the mitochondria (**Supplementary Figure 7A**), neither of them (or Thapsigargin) induced mitochondrial ROS immediately in Jurkat T cells (**Supplementary Figure 7B**). In addition, DMGV inhibited TCR-induced OxPhos *via* ROS in mouse and human T cells which were activated overnight (**Supplementary Figure 7C**). In Jurkat cells, DMGV inhibited OxPhos (**Figure 5A**) and did not increase the mitochondrial membrane potential as TCR stimulation did (**Figure 5B**). Therefore, we looked for a different mechanism to explain how DMGV induces ROS. A recent report (45) showed that ROS can be generated by a sudden influx of Na^+^ *via* the mitochondrial Na^+^/Ca^2+^ exchanger (NCLX) in response to a sharp rise of the mitochondrial Ca^2+^. Na^+^ promotes superoxide anion formation by uncoupling the Q cycle, which traps semiquinones in the Complex III Q0 site. *Ciona intestinalis* alternative oxidase (AOX), when ectopically expressed, oxidizes trapped ubiquinol to ubiquinone and thus inhibits superoxide anion formation (46, 47) by the same way MitoQ does. Indeed, expression of AOX completely inhibited ROS generation either by DMGV or kaempferol (**Figure 5C**), suggesting that DMGV- or kaempferol-induced Ca^2+^ generates superoxide anion by uncoupling the Q cycle. Next, we tested whether DMGV induces Na^+^ influx to the mitochondria using the Na^+^ indicator Coro-Na Green, which was mostly localized in the mitochondria (**Supplementary Figure 7D**). DMGV, but not TCR stimulation, induced Na^+^ influx into the mitochondria *via* NCLX in an MCU-dependent manner (**Figure 5D**). Inhibition of NCLX by CGP37157 inhibited DMGV-induced ROS without affecting the calcium flux induced by DMGV (**Figure 5E**). Furthermore, CGP37157 alone increased the mitochondrial calcium level without affecting the ROS level (**Figure 5F**), indicating that there is a steady-state Ca^2+^-Na^+^ exchange in the mitochondria and a calcium surge alone is insufficient to generate mitochondrial ROS. CGP37157, just like MitoQ, almost completely restored T cell proliferation from the inhibition by DMGV in human T cells (**Figure 5G**). Co-stimulation of CD3 and CD28 or PMA and ionomycin induced mitochondrial ROS whereas none of them did alone (**Supplementary Figure 7E**). Although PMA did not induce calcium flux, it enhanced ionomycin-induced calcium influx to both cytosol and mitochondria; together they induced Na^+^ entry to the mitochondria (**Supplementary Figure 7F**). These data together demonstrate the essential role of Na^+^-mediated uncoupling the Q cycle in the ROS generation by DMGV. We finally investigated why DMGV had no impact on T cell survival in a glucose-free medium (**Figure 2J**). DMGV did not induce ROS in a glucose-free medium largely due to the lack of NCLX activity (**Figure 5H**), confirming the importance of NCLX in DMGV-induced ROS generation.

### DMGV as a Therapeutic for Lymphoproliferative Diseases and Cancers

**Figure 6:**
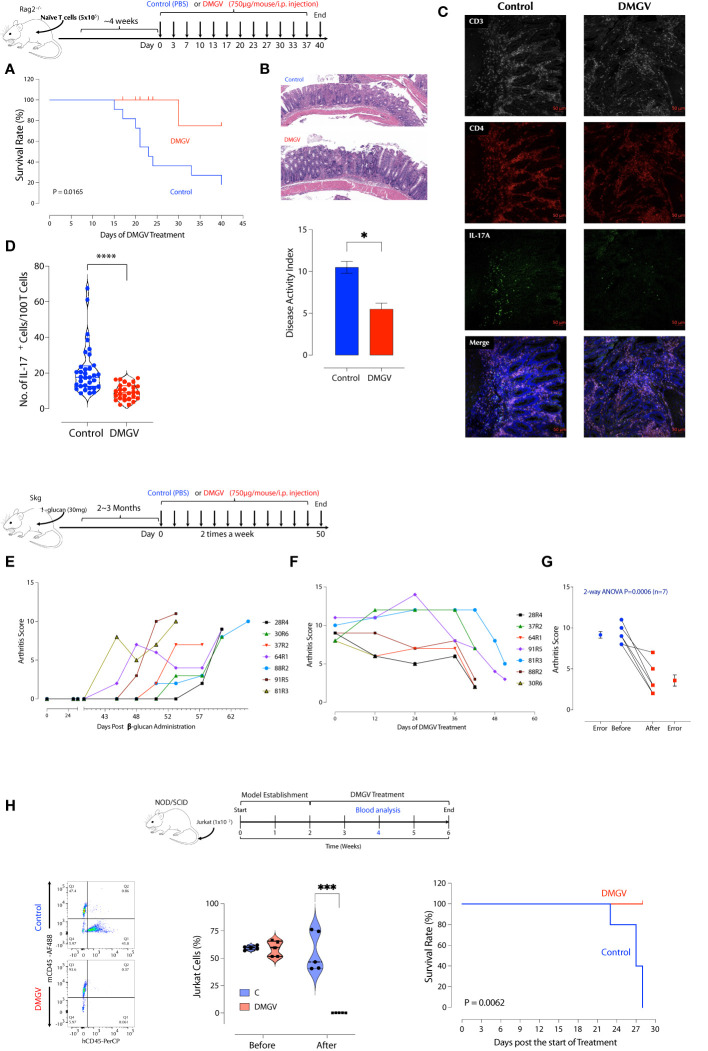
DMGV suppresses inflammation in animal models of IBD and RA. **(A)** RAG2^-/-^ deficient BALB/c mice were injected i.p. with 5x10^5^ naïve CD4^+^ T cells from BALB/c mice. Mice showing loose stool were immediately treated with PBS or DMGV (750µg in water/mice) i.p. twice a week. The experiment was terminated after 40 days of treatment. The statistical significance was calculated with the Kaplan-Meier method. **(B)** The representative colon H&E staining shows that the control group had more severe inflammation (ulceration, immune infiltrates) compared to the treated group. The disease activity index was measured as previously described (49). **(C)** The representative IL-17A staining in the colon section. **(D)** The morphometric analysis in the colon section shows a significant reduction in the IL-17A^+^ cell counts after the DMGV treatment. (Each dot represents a single confocal micrograph.) **(E)** SKG mice (n=16) were injected with laminarian (β-glucan, 10mg/mouse) and the arthritis score was measured weekly. Mice reaching the threshold score of 8 were selected and immediately treated with DMGV. **(F)** The selected mice (n=7) were administered i.p. with DMGV (750µg in water/mouse) twice a week. The experiment was terminated at the same time although the treatment started at different time points. **(G)** The statistical significance was calculated with 2-way ANOVA. **(H)** DMGV eliminates T cell leukemia in a mouse model. The model (Top panel) was generated by injecting 1x10^7^ Jurkat cells per NOD/SCID mouse i.v. and measuring the Jurkat to mouse CD45^+^ cell ratio in the peripheral blood after 2 weeks by flow cytometry. PBS or DMGV was injected as indicated and the final analysis was made after 2 weeks of treatment. The same results were obtained in two separate experiments. * < 0.05, *** < 0.001, **** < 0.0001.

As exogenous or excessive intrinsic DMGV (e.g. AGXT2 OE) inhibits survival and expansion of activated lymphocytes, we investigated whether it can suppress inflammation in T cell-mediated diseases in animal models. First, *in vitro* DMGV was able to inhibit survival of dividing WT T cells and rescue AGXT2^-/-^ T cells even when it was added 1 or 2 days after stimulation (**Supplementary Figure 8A**). Next, we tested whether DMGV inhibits T cell proliferation *in vivo*. Mouse total T cells were adoptively transferred to SCID mice to elicit lymphopenia-induced proliferation and 2 weeks later we injected DMGV (1mg/mouse) i.p. for a week. DMGV inhibited division of T cells as well as other cells in the spleen and colon (**Supplementary Figures 8B, C**). Adoptive transfer of naïve CD4^+^ T cells from syngeneic mice into immunodeficient mice induces chronic colitis, similar to inflammatory bowel diseases (IBD) in humans, due to differentiation of T cells into pathogenic Th1 and Th17 cells in the absence of regulatory T cells (Tregs) (48). After adoptively transferring naïve CD4^+^ T cells from syngeneic mice to recombinase activating gene-2-deficient (*Rag*2^-/-^) mice, the mice showing the colitis symptom in the form of loose stool in 4-6 weeks were randomly divided into two groups. One group (n=9) was treated with DMGV (i.p. 750µg/mouse, twice a week) and the control group (n=11) with PBS up to 40 days and mortality was observed. At the end of the treatment, all but 2 mice died in the control group whereas all except for one mouse survived in the DMGV-treated group (**Figure 6A**). The survived control mice showed more severe inflammation with ulceration and infiltrates in the colons while DMGV-treated mice showed significantly less inflammation (**Figure 6B**). The normalized IL-17A^+^ T cell number was significantly lower in the DMGV treatment group compared to the control group (**Figures 6C, D**). SKG mice harbor a point mutation in ZAP-70^W163C^, which leads to aberrant T cell activation and eventually to chronic autoimmune arthritis similar to human RA (50), which can be accelerated by injection of β-glucan (51). Laminarian (β-glucan, 30mg/mouse) was administered i.p. to SKG mice to induce the disease. In a pilot study, we tested whether DMGV can prevent the progression of arthritis. Mice (n=3) with a moderate arthritis score (AS 4-5) were treated with PBS or DMGV for 2 weeks and the arthritis score was monitored. Arthritis in all control mice became severe whereas DMGV-treated mice maintained the same level for the duration (**Supplementary Figure 8D**). In a longer-term study, we tested whether DMGV can treat severe arthritis. The treatment with DMGV (i.p. 750µg/mouse, twice a week) began with any mouse reaching the threshold arthritis score (51) of 8 (**Figure 6E**). The treatment was terminated when DMGV significantly decreased the arthritis score in all treated mice (**Figures 6F, G**). The ankle tissues of a diseased mouse showed IL-17^+^ T cells along the synovial membranes whereas few IL-17^+^ T cells were observed in the ankle of a normal SKG mouse (**Supplementary Figure 8E**). The histology and IL-17^+^ T cells in the hind ankles were consistent with the arthritis scores (**Supplementary Figure 8F**). Finally, we tested whether DMGV can induce cell death in hyperproliferative blood cancer cell lines. DMGV induced cell death within 3 days in Jurkat (without TCR stimulation, acute T cell leukemia), THP-1 (acute monocytic leukemia), HL-60 (acute promyelocytic leukemia), and K-562 (chronic myelogenous leukemia, CML) in a dose-dependent manner (**Supplementary Figure 9A**). It also induced cell death in the human colorectal cancer cell line HCT-8 and was much more effective when cells were cultured in 3D (Matrigel) than in 2D (**Supplementary Figure 9A**). It induced cell death by the same mechanism as in normal T cells since Jurkat cells were rescued from DMGV-induced death by anti-oxidant (NAC), the MCU inhibitor Ru360, NCLX inhibitor CGP37157, TGFβ, pyruvate, and βNMN (**Supplementary Figure 9B**). Next, we tested DMGV in a T cell leukemic model in NOD/SCID mice (**Supplementary Figure 9C**). A 2-week of DMGV treatment completely eliminated engrafted T cells, and all control mice died within 2 weeks after the treatment while all treated mice survived (**Figure 6H**). These data demonstrate DMGV as a potential therapeutic agent for lymphoproliferative diseases including autoimmune diseases, and hematological cancers, and potentially solid tumors. To evaluate the potential of DMGV as an oral drug, we performed a DMPK (Drug Metabolism and Pharmacokinetics) study in rats. DMGV (12.5mg/kg) was injected either I.P. (intraperitoneal) or delivered by gavage (PO: Per Os) to Sprague Dawley (SD) rats. The plasma DMGV level peaked at 0.25h after I.P. injection and 0.833h after P.O. with the relative bioavailability [F_Rel_] of 15% (**Supplementary Figure 10**). No adverse effects were observed after DMGV administration by either route.

## Discussion

**Figure 7:**
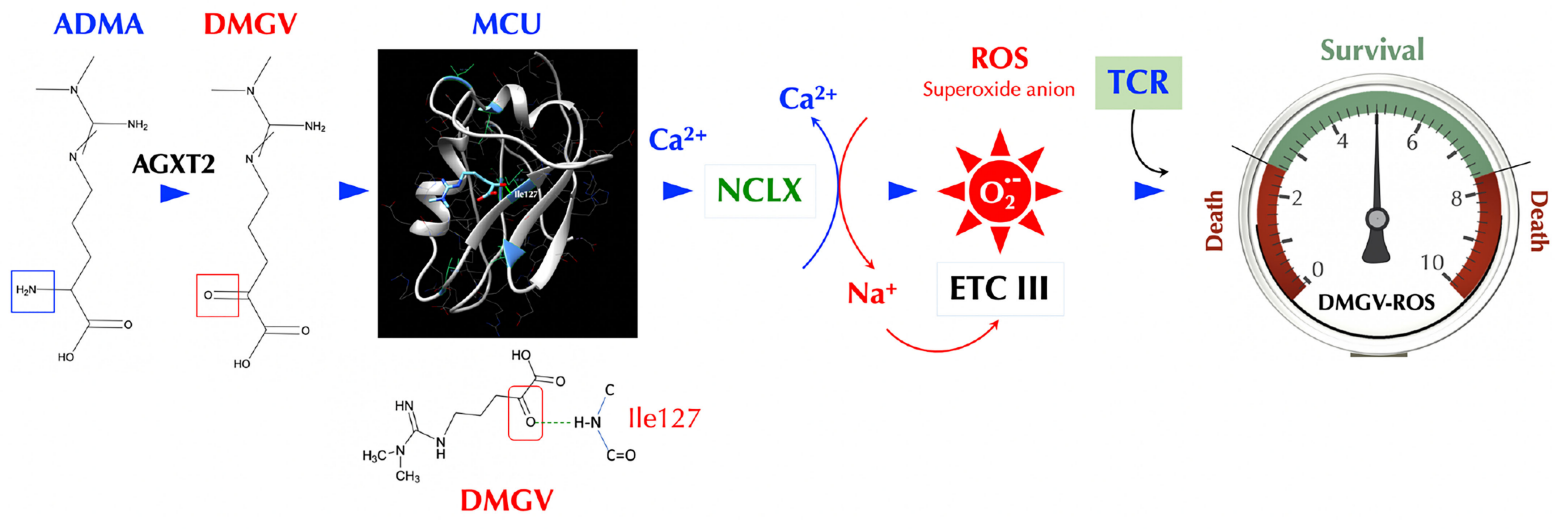
DMGV is a rheostat of T cell survival. When ADMA (or SDMA) is converted to DMGV by AGXT2 in the mitochondria, DMGV binds to MCU *via* a hydrogen bond at Ile127 and activates it to induce calcium import to the mitochondria, which triggers ER to release calcium. A rapid calcium ion rise in the mitochondria activates NCLK which exports calcium ions out of and imports sodium ions into the mitochondria. Sodium ions (positive charge) bind to the inner leaflets of the mitochondrial membrane (negative charge), which makes the membrane rigid and restricts the movement of semiquinones. This uncouples the Q cycle by trapping semiquinones in the Complex III Q0 site, thus generating superoxide anions (45). There is the range of AGXT2-DMGV-MCU-ROS levels that support survival and expansion of T cells while T cells do not survive outside that range.

ADMA has been described mainly as a natural inhibitor of NOS so far and suspected as a culprit in several diseases including cardiovascular diseases (52, 53), hypertension (27), and kidney disease (25). It is notable that the ADMA levels were found to be positively correlated with the cumulative inflammatory burden in RA patients (54). However, no definitive adverse role of ADMA in these diseases has been established to our knowledge. Daily ADMA treatment enhanced the blood-brain barrier disruption in experimental autoimmune encephalomyelitis mice and exacerbated the clinical and central nervous system disease, suggesting ADMA as a NOS inhibitor can be harmful in certain inflammatory conditions (55). ADMA also promoted Th1 and Th17 immune responses without affecting Treg response in the same study, the latter of which is inconsistent with our findings and needs to be further evaluated. Nonetheless, using NOS inhibitors such as ADMA as a treatment for inflammatory diseases needs more careful investigation. Here we presented an entirely new physiological function of ADMA, SDMA, and their metabolite DMGV. After being metabolized to DMGV by AGXT2, the cell-intrinsic level of DMGV is essential for survival and expansion of Teff cells but not Treg cells. However, a high level of DMGV, cell-intrinsic or exogenous, inhibits survival and expansion of lymphocytes, thus functioning as a rheostat of Teff survival and making DMGV a potential therapeutic for lymphoproliferative diseases. Our data showed the mitochondrial ROS, induced by DMGV and essential for T cell expansion, is regulated at multiple levels, i.e. AGXT2, MCU, and NCLX (**Figure 7**). A previous report found that various methylarginines including ADMA and SDMA were elevated while the DMGV level was decreased in the circulation of the AGXT2^-/-^ mice, which were hypertensive (56), suggesting a physiological importance of the enzyme. In another report, AGXT2^-/-^ mice exhibited hyperoxaluria and crystalluria and half of the male mice in mixed genetic background developed calcium oxalate urinary stones (57). However, neither report assessed the role of mitochondrial ROS or immune functions in these abnormalities. As the physiological level of ROS is essential for activation of NFAT, c-Myc, and HIFα needed for sustained T cell proliferation (58) and DMGV appears to be a major mitochondrial ROS inducer, more studies are warranted on the role of AGXT2 in T cell biology and immunity. While DMGV and the flavonoids induces mitochondrial calcium flux strictly *via* MCU, mitochondrial calcium flux upon TCR stimulation was independent of MCU. MCU-KO mice are born without much defect although MCU^−/−^ mitochondria lost the ability uptake calcium upon stimulation by histamine or isoproterenol (59). Our data indicate that there is an MCU-independent calcium transporter in the mitochondria, which is essential for TCR signaling but yet to be discovered. As we demonstrated with 3 different animal models, the fact that DMGV functions as a rheostat of T cell survival and expansion can be exploited for the treatment of autoimmune/lymphoproliferative diseases and cancers. In the case of autoimmune diseases caused by uncontrolled expansion of Teff due to TGFβ deficiency, our data suggest that AGXT2 inhibitors may suppress inflammation as our data show T cells are dependent on these two signals to survive *in vivo*.

## Materials and Methods

### Cell Culture

Cells were routinely maintained in cell culture incubators (95% air, 5% CO2 in gas phase, at 37°C). MEF (mouse embryonic fibroblasts), 16HBE, THP-1, HL-60, K562, HCT-8 and Jurkat cells were purchased from American Type Culture Collection and cultured in DMEM or RPMI medium from Gibco, supplemented with 10% heat-inactivated fetal bovine serum (Biological Industries, USA), 100U/mL penicillin and 100μg/mL streptomycin (ThermoFisher Scientific). All cultures were routinely tested for Mycoplasma contamination.

### Collection of Conditioned Medium (CM)

When MEF or 16HBE cells reached the confluency of 60%-70% in T75 fSKG, the cells were washed with PBS 3 times and the medium was replaced. CMs were collected after 24h and filtered with Steriflip^®^ Vacuum Driven Sterile Filter (Millipore).

### Purification of Soluble Factors From CM

To obtain small molecules in the CM, the pooled CM was applied to the 15ml Pierce protein concentrator (ThermoFisher Scientific, with the cutoff molecular weight of 3KD, and centrifuged at 2,000xg for 30min. The bottom fraction containing molecules below 3KD was lyophilized and dissolved in ddH_2_O (1/8 the original volume). The concentrated CM<3KD was desalted and partially purified with 5ml polyacrylamide desalting column (ThermoFisher Scientific) by gravity following the manufacturer’s protocol. Fractions obtained from desalting (10% vol/vol) was then tested in T cell proliferation assay. The positive fractions were pooled and separated by HPLC with ultrapure water as the mobile phase isocratically on a reverse phase C18 analytical (4.6×250 mm, 5μm) column (Guangzhou Research&Creativity Biotechnology Co., Ltd) at the flow rate of 0.3 mL min^-1^. The UV detector was set at 210 nm.

### Mass Spectrometry

The activity peak from HPLC was loaded onto Orbitrap Elite hybrid mass spectrometer (ThermoFisher Scientific) coupled to EASY-nLC II system (ThermoFisher Scientific) using the Xcalibur version 2.7.0 SP1 (ThermoFisher Scientific). The MS analysis was conducted in data-dependent acquisition where one high resolution (120000) FTMS full scan (m/z 300–1700) was followed by top20 CID-MS2 scans in ion trap (energy 35). Only the precursor ions with over 500 ion counts were allowed for MSn. Charge state rejection was enabled as well as dynamic exclusion which was fixed at 30s for the selected ions.

### Nucleic Acid Extraction and RNA Reverse Transcription

Total RNAs were extracted using Trizol (ThermoFisher Scientific) or NucleoZOL (TaKaRa) according to the manufacturer’s protocol. Extracted RNA was dissolved in RNase- and DNase-treated water and then immediately reverse-transcribed using HiScript Q RT SuperMix for qPCR (Vazyme). cDNA was stored at −20°C for subsequent qPCR analyses.

### Isolation of Human PBMCs

For human PBMCs and T cell isolation, the study was approved by the institutional ethics committee of School of Basic Medical Sciences of Guangzhou Medical University and a signed informed consent was obtained from every participant before being included into the study. blood was drawn into heparin sodium-containing tubes (K2E Vacutainer, BD) from healthy volunteers in the laboratory (non-shipped). Samples were processed within 2 hours. The time interval between the two blood drawings of each individual was 20–30 days and none of the individuals showed or reported any disease symptoms. All steps of PBMC preparation were carried out at room temperature. The blood from the same donor was pooled and mixed 1:1 with pre-warmed (room temperature) 1×PBS (without Ca^2+^ or Mg^2+^). The mixture was layered onto Lymphoprep (Stem Cell Technologies) in a SepMate™ tube and centrifuged at 1200rcf for 10min. The top layer containing enriched mononuclear cells was poured into a new tube and washed with PBS containing 2% FBS twice by centrifuging at 300rcf for 8min. Cells were resuspended in RPMI1640 medium for subsequent procedures.

### Flow Cytometry

Flow cytometry was performed with BD FACS Verse or BD FACS Aria II (San Jose, USA) for various assays described in detail below.

### Isolation of Mouse and Human Lymphocytes

Mouse splenic CD3^+^ T cells or CD19^+^ B cells, or human peripheral T cells from PBMCs were magnetically isolated according to the manufacturer’s instructions (Stem Cell Technology) and the purity of CD3^+^ or CD19^+^ cells were confirmed to be above 95% by flow cytometry.

### Cell Proliferation and Cytotoxicity Assay for Adherent Cells

Cell Counting Kit-8 (CCK-8) was used to determine the effects of DMGV on adherent cells at 72 hours according to the manufacturer’s instruction (Absin). For 3D culture, Matrigel™ (Corning) was diluted in culture medium at 1:3, mixed with cells in an equal volume, and plated in a 96-well round-bottom plate. Next day, cells were treated with DMGV and viability was measured after 72h.

### RNA Sequencing

Total RNA was extracted using TRIzol (ThermoFisher Scientific). RNAseq libraries were prepared using the VAHTS TM mRNAseq V2 Library Prep Kit for Illumina^®^ (Vazyme #NR601), and libraries were sequenced on an Illumina Xten to a read depth of 25–35 million reads per sample. Sequencing quality was assessed using Agilent 4200 Bioanalyzer. The sequencing data was filtered with SOAPnuke (v1.5.2) to remove the reads containing the sequencing adapter; Reads whose low-quality base ratio (base quality less than or equal to 5) was more than 20% were removed; After removing reads whose unknown base (‘N’ base) ratio is more than 5%, the clean reads were obtained and stored in FASTQ format. The clean reads were mapped to the reference genome using HISAT2 (v2.0.4), and then expression level of gene was calculated by STRING TIE (v1.2.3). To get an insight to the change of phenotype, GO (http://www.geneontology.org/) and KEGG (https://www.kegg.jp/) enrichment analysis of annotated different expressed gene was performed by Phyper based on Hypergeometric test. The significant levels of terms and pathways were corrected by P value with a rigorous threshold (P value ≤ 0.05) by Bonferroni.

### Immunofluorescence Confocal Microscopy

HCT-8 cells were seeded in chambered coverglasses and transfected with the mouse AGXT2 construct. Next day, the cells were fixed and permeabilized using CytoFix/CytoPerm kit (BD Biosciences), and stained and washed in Perm/Wash buffer. For localization of MCU WT and mutants, transfected Jurkat cells, after fixing with 4% paraformaldehyde (PFA) and permeabilizing with PBS/0.1% triton X-100 (PBS-T), were stained with anti-HA antibody followed by anti-rabbit IgG-Alexa Fluor 555. After washing with PBS-T, cells were stained with anti-COX IV-Alexa Fluor 647 and DAPI, and cyto-spun. Frozen mouse colon tissues were, air-dried, fixed with 4% PFA, permeabilized with PBS-T, blocked with 2% rat serum in PBS for 30min, and then stained with anti-CD4-AF (Alexa Fluor) 647 (BioLegend), anti-IL-17A-AF488 (ThermoFisher) and DAPI. All samples were visualized under Zeiss LSM 880 confocal microscope.

### Transfection of Lymphocytes by Electroporation

For gene knockdown or overexpression, siRNAs and plasmids were introduced into cells by Neon Transfection System (Invitrogen) according to the manufacturer’s protocol. After the cells were pre-incubated in complete cell culture medium containing the nucleic acid transfection enhancer NATE (1%) for 30min, the electroporation pipette tip was filled with 2×10^6^ cells in transfection R buffer containing siRNA/plasmids. Three milliliters of Electrolytic buffer were added to a buffer container. The electrical conditions were set at 1,600V, 10ms, and 3 pulses. After electroporation, cells were rapidly dispensed into 5ml round bottom sterile tubes using a pipette, and were then cultured in a CO_2_ incubator at 37°C.

### Lentivirus Production and Infection

Lentiviruses were packaged by GenePharma Inc. (Shanghai, China) and cells were infected with the viruses in the presence of polybrene (5µg/ml) overnight. Some stable cell lines were generated by selection with puromycin (5µg/ml).

### Animals

All animal work was done following the Guide for the Care and Use of Laboratory Animal and approved by the institutional ethics committee of School of Basic Medical Sciences of Guangzhou Medical University, in accordance with the vivarium of Institute of Animal Health, Guangdong Academy of Agricultural Sciences. All efforts were made to minimize the number of animals used and their suffering. All mice were housed in specific pathogen-free conditions before use. Male and female mice were 6–8 weeks old at the time of use. C57BL mice were obtained from Jinan Pengyue Animal Center, and SKG mice were obtained from CLEA Japan, Inc. BALC/c and Rag2^-/-^ mice were purchased from GemPharmatech Co., Ltd. (China).

### Generation of AGXT2 KO Mice

AGXT2 gene of mouse was edited with CRISPR/Cas9 technology. The Agxt2 gene has 4 transcripts. Exon 4 of Agxt2-204 (ENSMUST00000110542.7) transcript was chosen as the knockout target. Cas9 and sgRNA (GGATGCAGTCCCTATACACT) were microinjected into the fertilized eggs of C57BL/6J mice. Fertilized eggs were transplanted to obtain positive F0 mice which were confirmed by PCR and sequencing. A stable F1 generation mouse model was obtained by mating positive F0 generation mice with C57BL/6J mice. Newly born mice were genotyped by PCR using two different primer sets below. Set 1 amplifies both WT (2.7kb) and KO (297bp), and set 2 only amplifies WT (366bp). The primer set 1 are 5’- ATCTCACCGCATTCCTATCACTACC-3’ (forward) and 5’- TCCTAGAGACTCACAGTGGTTAGCAG-3’ (reverse); the primer set 2 are 5’-TCTTGAAGAAGTAGCCTGGGGTC-3’ and 5’- CCTACTTTCTTCAGCTTGAGTGCTC-3’ (reverse).

### Gene Knockout in Cells by Crispr/Cas9

For human MCU knockout, a Crispr/Cas9 construct in LGE-4(LentiV2-gRNACas9Puro) containing the sgRNA was packaged in Lentiviruses (GenePharma, China). Jurkat cells were infected with either control viruses or MCU-KO viruses and selected with puromycin. KO was confirmed by qPCR. For some experiments, Jurat cells were co-infected with Mito-GEM-GECO carrying viruses and MCU-KO viruses, and selected. For RyR1 or ITPR1 knockout, sgRNA was transfected into the Jurkat control cells carrying Cas9 [LGE-4(LentiV2-gRNACas9Puro) by electroporation. Short guide RNA for human MCU was 5’-TTTTCATTCAGATGTTACAG-3’; RyR1 5’-GACTACGTAACGGATCCCCG-3’ for human; 5’-GAGGCGGGCATATTTCACGG-3’ for human ITPR1.

### T and B Cell Proliferation Assay

Mouse or human T cells were labeled with CellTrace Violet (ThermoFisher Scientific) according to the manufacturer’s protocol. Mouse T cells were stimulated *in vitro* with anti-CD3 and -CD28 antibody coated beads (Gibco Dynabeads) and human T cells with anti-CD3 (5µg/ml) and -CD28 antibody (2µg/ml) coated on a 96-well plate, and expanded in RPMI1640 medium with 10% FBS up to 5 days. Dilution of CellTrace Violet was then evaluated by flow cytometry. Mouse B cells were stimulated with anti-IgM (20μg/ml) and IL-4 (10ng/ml) for 4-5 days.

### Cell Cycle Assay With PI Staining

Stimulated T cells were centrifuged and re-suspended in Cell Cycle Dye (Yishan Biology, Shanghai, China), incubated at room temperature for 30min according to manufacturer’s instructions. Cells were then analyzed by flow cytometry, and during analysis, several datasets, including forward scatter *vs*. side scatter, pulse area *vs*. pulse width, and cell count *vs*. propidium iodide, are collected to ensure only single cells are measured.

### Annexin V/PI Staining

For apoptosis analysis, Annexin V-APC/PI staining was performed using flow cytometry according to the manufacturer’s guidelines. Briefly, cells were incubated with PI and Annexin V-fluorescein isothiocynate in darkness at room temperature. Flow cytometric analysis was immediately performed.

### Antibodies

The following antibodies were used for the present study.

Anti-mouse CD3, clone number 17A2, BioLegend, 05112-40-100, V450;

Anti-mouse CD4, clone number GK1.5, BioLegend, 100434, PerCP/Cyanine5.5;

Anti-mouse CD8, clone number 53-6.7, BioLegend, 100714, APC/Cyanine7;

Anti-mouse CD25, clone number 3C7, BioLegend, 101908, FITC;

Anti-mouse CD69, clone number H1.2F3, BioLegend, 104508, PE;

Anti-mouse CTLA-4, clone number UC10-489, BioLegend, 106305, PE;

Anti-mouse PD-1, clone number 29F.1A12, BioLegend, 135210, APC;

Anti-mouse CD19, clone number 1D3, eBioscience, 12-0193-82, PE;

Anti-mouse CD45, clone number 30F11, BioLegend, 103122, Alexa Fluor 488;

Anti-human CD45, clone number HI30, BioLegend, 304025, PerCP;

Anti-mouse IFNγ, clone number XMG1.2, eBioscience, PE;

Anti-mouse IL-17A, clone number TC11-18H10.1, BioLegend, Alexa Fluor 647;

Anti-mouse IL-13, clone number 13A, BioLegend, PE/Cyanine7;

Anti-mouse Foxp3, clone number MF14, BioLegend, PE;

Anti-mouse TNFα, clone number MP6-XT22, BioLegend, Alexa Fluor 488;

Purified anti-mouse IFNγ antibody, clone number XMG1.2;

Purified anti-mouse IL-4 antibody, clone number 11B11.

### Mouse T Cell Differentiation

Naïve CD4^+^ T cells were isolated from the spleens according to the manufacturer’s instruction (Stem Cell) and stimulated with addition of IL-2 (30U/ml) and TGFβ (15ng/ml) for Tregs or TGFβ (15ng/ml), IL-6 (10ng/ml), anti-mouse IFNγ Ab (5µg/ml) and anti-mouse IL-4 Ab (5µg/ml) for Th17 for 4-5 days. Foxp3 was stained using FoxP3 Staining Kit (BD560131) according to the instruction.

### Intracellular Cytokine Measurement

T cells were stimulated with PMA (50ng/ml) and ionomycin (1µg/ml) in the presence of brefeldin A (1µg/ml) for 4h. Cells were fixed and permeabilized with BD Cytofix/CytoPerm for 10min, washed in PermWash buffer, stained with the indicated antibodies in PermWash buffer for over 2h, and analyzed by flow cytometry.

### DSS Colitis and Analysis

Mice were given 2.5% DSS (40,000 kDa; MP Biomedicals) ad libitum in drinking water for 7 days, followed by normal drinking water for 2 days. Control mice received PBS i.p. injection while Treated group received DMGV (750µg/mouse/injection) i.p. injection. In all experiments, water was changed twice a week. Body weight loss was determined, and mice were monitored for rectal bleeding, diarrhea and signs of morbidity over the course of disease. On day 10 post-DSS or body weight loss reached 20% of original, mice were killed. Colon length was measured. Colon sections were cut into two halves longitudinally and one half was embedded in OCT and frozen in liquid nitrogen and the other half was fixed in 10% phosphate buffered formalin. The frozen sections were cut using microtome and formalin fixed ones were paraffin embedded, processed and stained with H&E by Servicebio (Guangzhou, China).

### Seahorse Assay

Cells were washed three times in complete Seahorse medium (Seahorse Bioscience) with 10mM glucose, 1mM sodium pyruvate and 2mM glutamine. Cells were plated at 2×10^6^ cells per well in a 24-well Seahorse assay plate pretreated with poly-d-lysine. OCR (pmoles/min) and ECAR (mpH/min) were measured as indicated upon cell treatment with first DMGV or anti-CD3 stimulation, followed by oligomycin (2μM), FCCP (1.5μM) rotenone/antimycin A (0.5µM), according to the manufacturer’s instructions. For glycolysis stress test assay, cells were plated at 2×10^6^ cells per well in a 24-well Seahorse assay plate pretreated with poly-d-lysine. OCR (pmoles/min) and ECAR (mpH/min) were measured as indicated upon cell treatment with first DMGV or anti-CD3 stimulation, followed by glucose (10mM), oligomycin (1μM) and 2-DG (50mM), according to the manufacturer’s instructions.

### Calcium Flux Assay

Intracellular calcium was measured using the non-wash calcium assay Fluo8 kit (ab112129, Abcam) according to the manufacturer’s instructions. Briefly, T cells were spun down (500rcf, 5min), supernatant was aspirated, and calcium dye was added in calcium containing buffer for calcium influx assay or Mg^2+^/Ca^2+^-free PBS for calcium efflux assay. During the dye loading, inhibitors were added. Following the incubation for 30 min at 37°C, calcium flux induced by various agents was measured by time-lapse flow cytometry with agents added at 20s.

### Measurement of Intracellular ROS

For flow cytometry, cells were loaded with 5μM CellROX Green Reagent or Deep Red for 30 min at 37°C, during when different inducers or inhibitors were also added for MFI. Cells were washed in 300μl of PBS and then analyze with flow cytometry. For the time-lapse experiments, indicated inducers were injected to the cells 20sec after acquisition started and then acquisition lasted for different time length for specific experiments.

### Measurement of Mitochondrial Membrane Potential (Δψm)

For analysis of Δψm, T cells were put in sterile 5ml round bottom tubes at a final density of 1.5×10^5^ cells/tube. After incubation with various reagents, cells were loaded with 200nM TMRE dye for 15min at 37°C and spun at 500 rcf for 5 min. The cell pellet was washed with 1 ml warm 1X PBS, resuspended in 300 µl warm 1X PBS (for tubes), and analyzed by flow cytometry (BD FACS Verse, San Jose, USA). For the time-lapse experiments, cells were pre-loaded with TMRE and indicated inducers were injected to the cells 20sec after acquisition started.

### Measurement of Mitochondrial Na^+^

Jurkat cells were washed three times with HBSS containing Ca^2+^/Mg^2+^/glucose, incubated for 1h with 10μM Coro-Na Green-AM, washed three times with HBSS containing Ca^2+^/Mg^2+^/glucose, and incubated for an hour in HBSS containing Ca^2+^/Mg^2+^/glucose. After washing three more times with HBSS containing Ca^2+^/Mg^2+^/glucose, the cells were resuspended in HBSS containing Ca^2+^/Mg^2+^/glucose and analyzed by time-lapse flow cytometry. Coro-Na Green was visualized by confocal imaging as follows. Jurkat cells stained with Coro-Na Green as above and stained with MitoTracker Deep Red at the last hour of staining. The cells were washed and fixed with 4% PFA, cyto-spun, and mounted for confocal imaging.

### Synthesis of DMGV

DMGV was synthesized (60) and verified by mass spectrometry and NMR (data not shown) as described (61, 62).

### Molecular Docking

The SwissDock web program (44) was used to assess whether DMGV or the flavonoids directly bind to MCU (63). The flexibility of side chains was allowed within 5Å of any atom of the ligand in the reference binding mode.

### Animal Model of Colitis

*Rag*2^-/-^ mice were injected i.p. with naïve CD4^+^ T cells from BALB/c mice (5x10^5^/mouse) and mice showing loose stool were divided into two groups. One group was treated with DMGV (i.p. 750µg/mouse, twice a week) and the control group with PBS up to 40 days and mortality was observed.

### Frozen Colon Section Preparation

The colons were rolled around a toothpick to form a Swiss-roll, which was then immersed in OCT medium in a plastic mold. The base mold containing the tissues was placed into liquid nitrogen till tissues were frozen completely. The frozen tissue block was sectioned (10µm thickness) using a cryotome, placed onto glass slides, and stored at -80°C.

### Quantification of IL-17A^+^ Cells in the Colon Sections

Confocal micrographs of two mice in each group were taken throughout the colon sections, and 7-10 micrographs/mouse were obtained from each colon. Using the pointer tool in ImageJ, CD3^+^ or IL-17A^+^ cells were manually counted and normalized as the number of IL-17A^+^ cells per 100 CD3^+^ cells in each micrograph. The difference in the IL-17A^+^ cell number between the two groups was analyzed by unpaired student T test.

### Animal Model of Arthritis

SKG mice (n=16) were injected i.p. with laminarian (β-glucan, 30mg/mouse) was administered to SKG mice to induce the disease (51), and the mice reaching the threshold arthritis score (51) of 8 (n=7) were immediately treated with DMGV (i.p. 750µg/mouse, twice a week) up to 50 days. The treatment ended at the same time point.

### Preparation of Paraffin-Embedded Joint Tissues

SKG mouse joints were cut and fixed with 10% formalin for 24-48 hours at room temperature, washed with PBS, and submerged in the decalcification medium (Leagene, China). The medium was changed every day and the tissues were paraffin-embedded when the needle penetrates the bone without much difficulty (~14 days). Paraffin embedding was done in the following sequence: 70% ethanol, 2 changes, 1hour each; 80% ethanol, 1 change, 1hour; 95% ethanol, 1 change, 1hour; 100% ethanol, 3 changes, 1.5 hour each; xylene, 3 changes, 1.5 hour each; paraffin wax (58-60°C), 2 changes, 2 hours each. The paraffin blocks were trimmed and cut at 5µm and placed in water bath at 40-40°C before being mounted onto slides. The sections were air-dried for 30min and baked in a 45-50°C oven overnight.

### Processing of the Hind Legs of SKG Mice for H&E

Hind legs were fixed in formalin for 24h and washed in 70% alcohol and decalcified in a decalcification solution (Leagene, China) for 10 days until a needle penetrates tissue with ease.

### Immunofluorescence of SKG Ankle Tissues

Tissue slices were deparaffinized and boiled in a water bath in citrate buffer (pH 6.0) to retrieve antigens. Once the tissues were cooled to room temperature, they were stained in PBS containing 0.1% Triton-100 with DAPI, anti-mouse Il-17A-Alexa fluor 488, and anti-mouse CD3-Alex fluor 594 for 1h at room temperature, and autofluorescence was quenched with TrueVIEW^®^ Autofluorescence Quenching Kit (Vector laboratories) before imaging by confocal microscopy.

### Animal Model of Hematological Cancer

NSG/SCID mice (6-8 weeks old) was purchased from —. Mice were injected with 1x10^7^ Jurkat cells in 1ml of PBS *via* tail vein. Establishment of engraftment was confirmed by flow cytometry 2 weeks post injection. Mice were randomly divided into two groups and injected with either PBS or DMGV (750µg/mouse in 200µl PBS) on days 0, 3, 7, 10, and 14. The level of Jurkat cells in peripheral blood was measured after retroorbital bleeding (200µl) and the body weight change was measured.

### DMPK (Drug Metabolism and Pharmacokinetics) Study in Rats

The study was performed at WuXi AppTec (Shanghai, China). DMGV was dissolved in double distilled water at 30mg/ml and the dosage of 12.5mg/kg was administered I.P. or P.O. (Per Os) to 7-10 week-old male SD rats (n = 3). The P.O. groups was fasted overnight before the administration. The blood was drawn at the indicated time points. The plasma DMGV level was measured by HPLC and mass spectrometry.

## Data Availability Statement

The datasets presented in this study can be found in online repositories. The names of the repository/repositories and accession number(s) can be found below: NCBI GEO, accession no: GSE203613.

## Ethics Statement

The studies involving human participants were reviewed and approved by Guangzhou Medical University. The patients/participants provided their written informed consent to participate in this study. The animal study was reviewed and approved by Guangzhou Medical University.

## Author Contributions

All authors contributed significantly to the drafting and editing of this manuscript. JL and FY conceived the manuscript idea and revised the manuscript content. FY, LS, and DF, and K-HC performed the experiments. All authors contributed to the article and approved the submitted version.

## Funding

This work was supported by Guangzhou Medical University Startup Fund to JL.

## Acknowledgments

We deeply appreciate Jinhua Chen for his advices and help on many chemical questions and Jose M. González-Navajas, Mike Murphy, Eyal Raz, and Samuel Bertin for a critical reading of the manuscript and helpful comments.

## Conflict of Interest

The authors declare that the research was conducted in the absence of any commercial or financial relationships that could be construed as a potential conflict of interest.

## Publisher’s Note

All claims expressed in this article are solely those of the authors and do not necessarily represent those of their affiliated organizations, or those of the publisher, the editors and the reviewers. Any product that may be evaluated in this article, or claim that may be made by its manufacturer, is not guaranteed or endorsed by the publisher.
