# Supplementary material for: DMGV Is a Rheostat of T Cell Survival and a Potential Therapeutic for Inflammatory Diseases and Cancers
Source: Front Immunol. 2022 Aug 5;13:918241. doi: 10.3389/fimmu.2022.918241 (PMC9389583; doi:10.3389/fimmu.2022.918241)

Materials and Methods

Table 1. Reagents used in the study

NAME	Cat. No.	Source
CellTrace Violet	C34557	Thermo Fisher Scientific
PD404182	HY-16958	MedChemExpress
L-Citrulline	HY-N0391	MedChemExpress
ADMA	T5309	TopScience
SDMA	T7344	TopScience
MitoQ,	T12059	TopScience
rapamycin	T1537	TopScience
BAPTA-AM	A1076	Merck
Thapsigargin	TQ0302-1mg-20	TopScience
ionomycin	HY-13434	MedChemExpress
Ru360	HY-122898	MedChemExpress
ryanodine	HY-103306	MedChemExpress
kaempferol	52314	Selleck
quercetin	M3902	Abmole
daidzein	M5547	Abmole
CGP37157	M7732	Abmole
Coro-Na Green	C36676	InvitrogenTM
Pyruvate	11360-070	Gibco
β-nicotinamide mononucleotide	M9580	Abmole
SB431542	M1794	Abmole
Human TGFβ		
CellROX™ Green Flow Cytometry Assay Kit	C10492	Thermo Fisher Scientific
CellROX™ Deep Red Flow Cytometry Assay Kit	C10491	Thermo Fisher Scientific
Dynabeads™ Mouse T-Activator CD3/28	11452D	Thermo Fisher Scientific

Dynabeads™ Human T-Activator CD3/28	11131D	Thermo Fisher Scientific
NATE	lyec-nate	InvivoGen
MitoTracker™ Red CM-H2Xros	M7513	Invitrogen
N-Acetyl-L-cysteine	A7250	Merck
NIL (N6-(1-iminoethyl)-L-lysine)	482100	Merck
MitoSOX™ Red mitochondrial superoxide indicator	M36008	Thermo Fisher Scientific
tBHP (tert-butyl hydroperoxide)	C10492	CellROX™ Green Flow Cytometry Assay Kit (Thermo Fisher Scientific)
Mitochondrial Membrane Potential Assay Kit (TMRE)	13296s	Cell Signaling
Cell Counting Kit-8 (CCK-8)	abs50003	Absin
Matrigel	354248	Corning

Table 2. Antibodies used in the study

**Antigen**	**Fluorescent label**	**Clone Number**	**Source**
Mouse CD3	APC	17A2	Elabscience
Mouse CD19	FITC	1D3	BioGems
Human CD3	APC/Cy7	HIT3a	BioLegend
Human CD19	PE	HIB19	BioLegend
Human CD14	AF 488	HCD14	BioLegend
HA	-	C29F4	Cell Signaling
Human COX IV	AF 647	3E11	Cell Signaling
Mouse IL-17	AF 488	eBio17B7	ThermoFisher
Mouse CD3e	AF 594	17A2	BioLegend
Mouse CD4	PerCP/Cy5.5	GK1.5	BioLegend
Mouse IL-17A	AF 488	53-7177-81	ThermoFisher

Mouse CD3	AF 594	100240	BioLegend

Table 3. Lymphocyte isolation kits and PCR reagents

Name	Catalog No.	Source
EasySep™ Mouse T Cell Isolation Kit	19851A	Stem Cell Technologies
EasySep™ Mouse B Cell Isolation Kit	19854A	Stem Cell Technologies
EasySep™ Mouse Naïve CD4+ T Cell Isolation Kit	19765	Stem Cell Technologies
EasySep™ Human T Cell Isolation Kit	17951A	Stem Cell Technologies
EasySep™ Human CD8 T Cell Isolation Kit	17953	Stem Cell Technologies
ChamQ Universal SYBR qPCR Master Mix	Q711-02	Vazyme
Hieff UNICON qPCR SYBR Green Master Mix	11198ES03	Yeasen
HiScript III RT SuperMix for qPCR	R323-01	Vazyme
NucleoZOL	740404.200	TaKaRa Bio Inc.

Table 4. siRNAs used in the study

All double-stranded siRNAs against Mus musculus/Homo Sapiens genes were designed and purchased from Tsingke Biological Technology (China)

Gene name	Sense Sequence
Mouse AGXT2	UGAAAGAUACCAGUCCCUUTT
Mouse RYR1	GCCUCUUUCAUGGACAUAUTT
Mouse MCU	GGGAAUAAAGGGAUCUUAATT
Mouse PRMT1	CCGCAAGGUUAUUGGGAUUTT
Mouse PRMT5	GGAGUUCAUUCAGGAACCUTT
Human AGXT2	CAAGCUAAAGAUCAGUAUATT
Human RYR1	GCCUCUUUCAUGGACAUAUTT
Human MCU	AAGUAUCUCAGUUUCUGGAUGTT (3'-non-coding sequence)

Table 5. Plasmids used in the study

All expression constructs were chemically synthesized, cloned, and sequenced.

Insert	Vector	Tag
Mouse HA-AGXT2	pcDNA3.1(+)	None
Mouse HA-MCU-WT	pcDNA3.1(+)	HA
Human HA-MCU$^{\Delta Ile127}$	pcDNA3.1(+)	HA
Human HA-MCU$^{\Delta Ile127/Val135}$	pcDNA3.1(+)	HA
Human HA-MCU$^{\Delta Val135}$	pcDNA3.1(+)	HA
Mito-GEM-GECO[1]	LV5	None
AOX [2; 3]	pcDNA3.1(+)	None
Human MCU KO	LGE-4(LentiV2-gRNACas9Puro)	None

Table 6. qPCR Primers

Gene	Forward Primer (5'-3')	Reverse Primer (5'-3')
Mouse MCU	GAGCCGCATATTGCAGTACGGT	AAACACGCCGACTGAGTCAGAG
Human MCU	ACCGGACGGTACACCAGAG	GATAGGCTTGAGTGTGAACTGAC
Mouse RyR1	CGCACACAGTCGTATGTACCT	TAATCCCACGTCAAAGGCCAA
Mouse ITPR1	CGTTTTGAGTTTGAAGGCGTTT	CATCTTGCGCCAATTCCCG
Human RyR1	CTCCGCCTCTTTCATGGACAT	CTGCCCGGTAGTGACATGC
Human ITPR1	GCGGAGGGATCGACAAATGG	TGGGACATAGCTTAAAGAGGCA
Mouse AGXT2	CAGATAGACCGCCTGTGGCATA	GGCTTCTGATCCACTGTTCACC
AOX	GGTTCGTCACCTGGTTTCCT	CGCATCATTGCATCGTCAGG
Mouse PRMT1	TACTACTTTGACTCCTATGCCCA	ATGCCGATTGTGAAACATGGA
Mouse PRMT5	CTGAATTGCGTCCCCGAAATA	AGGTTCCTGAATGAACTCCCT
Mouse UBC	GCCCAGTGTTACCACCAAGA	CCCATCACACCCAAGAACA
Human GAPDH	ATCACCATCTTCCAGGAGCGAG	GGGCAGAGATGATGACCCTTTTG

[1] Y. Zhao, S. Araki, J. Wu, T. Teramoto, Y.F. Chang, M. Nakano, A.S. Abdelfattah, M. Fujiwara, T. Ishihara, T. Nagai, and R.E. Campbell, An expanded palette of genetically encoded Ca(2)(+) indicators. Science 333 (2011) 1888-91.

[2] G.A. Hakkaart, E.P. Dassa, H.T. Jacobs, and P. Rustin, Allotopic expression of a mitochondrial alternative oxidase confers cyanide resistance to human cell respiration. EMBO Rep 7 (2006) 341-5.
[3] I. Martinez-Reyes, L.R. Cardona, H. Kong, K. Vasan, G.S. McElroy, M. Werner, H. Kihshen, C.R. Reczek, S.E. Weinberg, P. Gao, E.M. Steinert, R. Piseaux, G.R.S. Budinger, and N.S. Chandel, Mitochondrial ubiquinol oxidation is necessary for tumour growth. Nature 585 (2020) 288-292.

Supplementary Data

Supplementary Figure 1. MEF secrete small molecules (ADMA and SDMA) that inhibit T cell proliferation

A. Cell survival and proliferation measurement strategy. The live gate in FSC/SSC graph corresponded to cell survival was measured by Annexin V/PI staining. Therefore, survival rates were measured in this way throughout the study with a few exceptions. **B.** A soluble factor(s) in MEF (but not in 16HBE) conditioned medium (CM) inhibits T cell proliferation. CellTrace Violet (CTV)-labeled mouse splenic $CD3^+$ T cells were activated by the immobilized anti-mouse CD3/CD28 antibodies (5μg/2μg/ml) with or without mouse embryonic fibroblasts (MEF) or 16HBE CM, and the survival rate and divided cell counts were measured at the indicated time points by flow cytometry (upper panel). The statistical significance was calculated by unpaired Student T test. (2 independent biological samples). **C.** Schematic representation of partial purification of the soluble factor (named as LNY1) from the MEF CM (left). To test the activity, each fraction was added to the culture medium (10% in volume) and the effects on mouse splenic $CD3^+$ T cell proliferation were measured at 48h (right). **D.** LNY1 was purified to homogeneity by HPLC from Fractions 4 and 5 as described in the method. **E.** LNY1 inhibits T cell proliferation. The indicated amount of LNY1 was added to splenic $CD3^+$ T cells and proliferation was measured at 72h. (n=3 independent biological samples, ****<0.0001). **F.** The mass/charge ratio of LNY1 was determined by mass spectrometry. **G.** Generation of LNY1 by MEF is dependent on PRMT1 and PRMT5. CMs were collected from MEF transfected with the indicated siRNA after 2 days and applied to mouse splenic $CD3^+$ T cell proliferation assay at 72h. **H.** Both ADMA and SDMA inhibit proliferation of mouse splenic $CD3^+$ T cells. The indicated amount of LNY1, ADMA, or SDMA was added to mouse splenic $CD3^+$ T cells, and survival and proliferation were measured at 72h. (n=2 independent biological samples, **<0.01, ***<0.001). **I.** Both ADMA and SDMA inhibit proliferation of mouse splenic $CD19^+$ B cells. LNY1 (100ng/ml), ADMA (10μM), or SDMA (10μM) was added to mouse splenic $CD19^+$ B cells stimulated with anti-IgM antibody plus IL-4, and survival and proliferation were measured at 72h as described in the method. (n=2 independent biological samples, *<0.05, **<0.01). **J.** Both ADMA and SDMA inhibit proliferation of human peripheral blood $CD3^+$ T cells. LNY1 (100ng/ml), ADMA (10μM), or SDMA (10μM) was added to human peripheral blood $CD3^+$ T cells, and the numbers of live and divided cells were measured by

flow cytometry after 4 days. (n=2 independent biological samples, *<0.05). **K.** ADMA and SDMA inhibit proliferation of pre-activated T cells. Mouse splenic CD3$^+$ T cells (left) and human peripheral blood CD3$^+$ T cells (right) were activated by the CD3/CD28 antibody-coated activation beads (mouse T cells, cells:beads=4:1), or immobilized anti-human CD3/CD28 antibodies (human T cells, 5μg/2μg/ml) for 24h, and then LNY1 (100ng/ml), ADMA (5μM) or rapamycin (100nM) were added as indicated. (n=2 independent biological samples, *<0.05, **<0.01). All the above data were repeated at least twice with the similar results.

Supplementary Figure 1

A

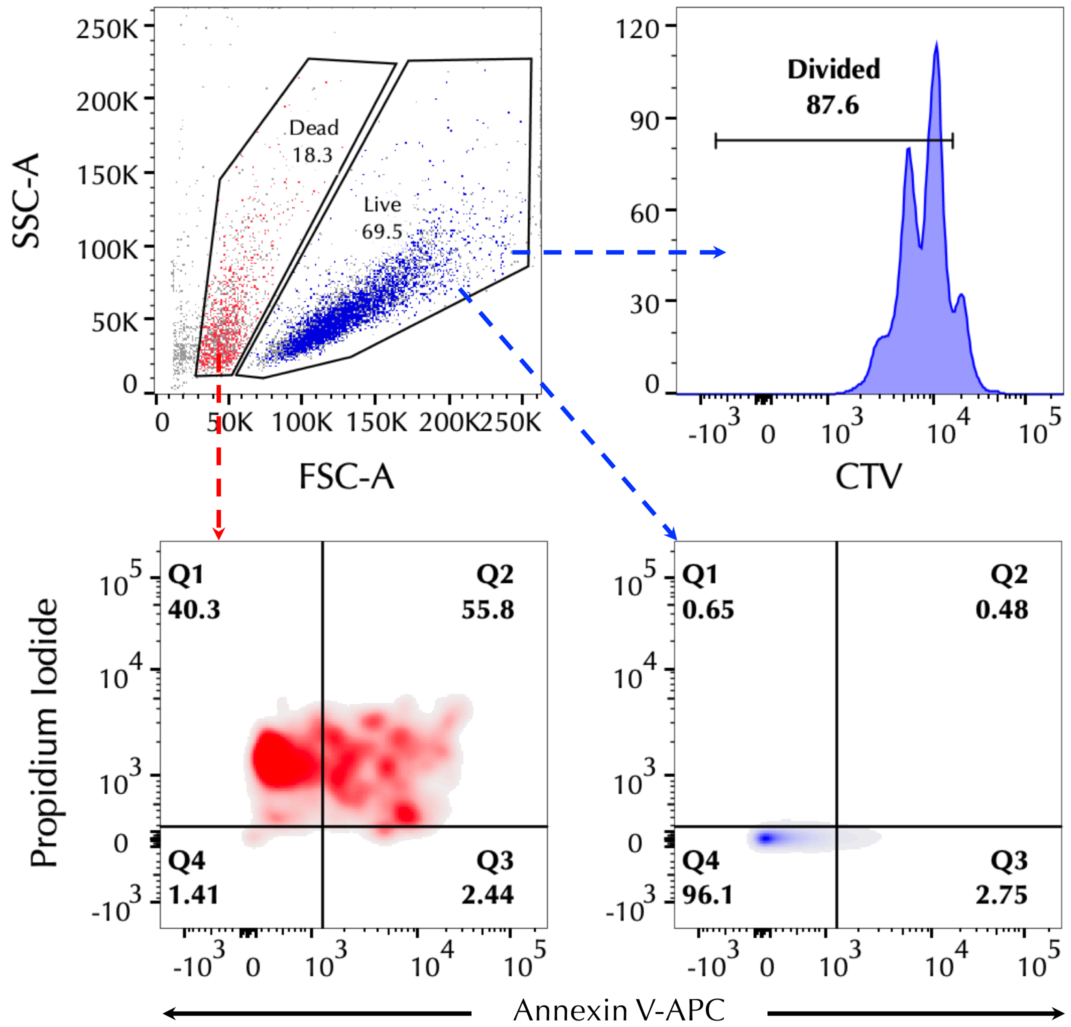

Supplementary Figure 1

B

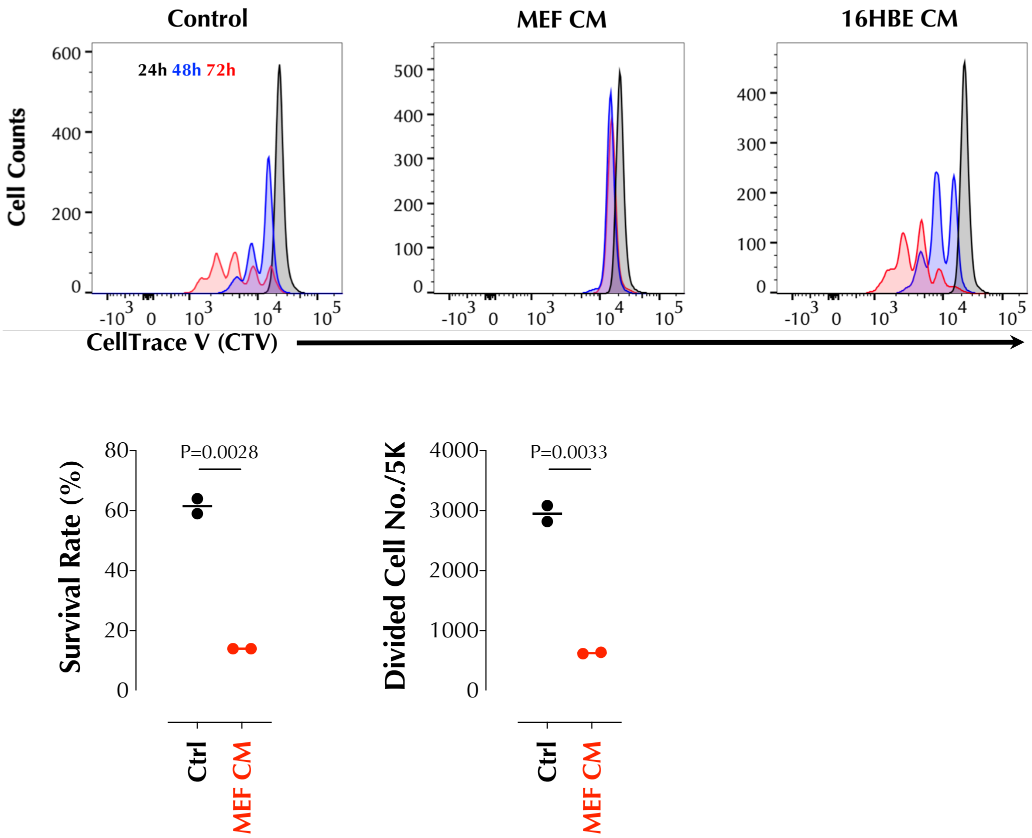

C

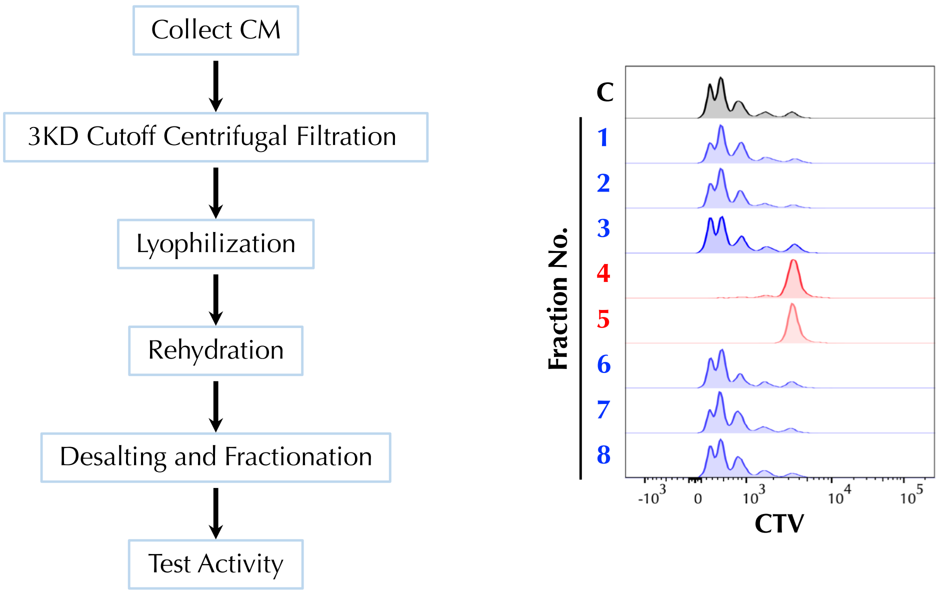

Supplementary Figure 1

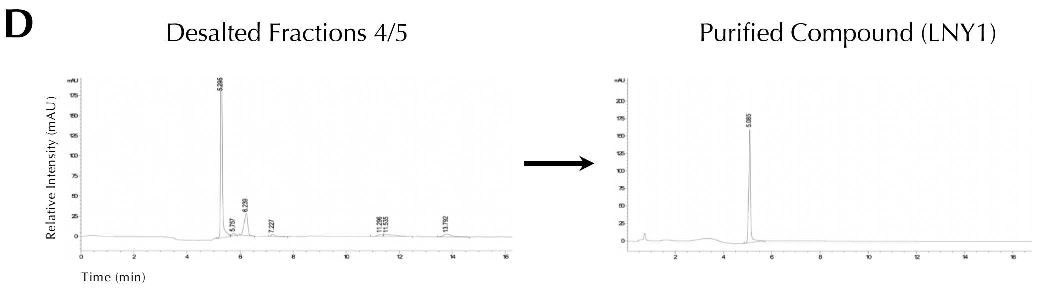

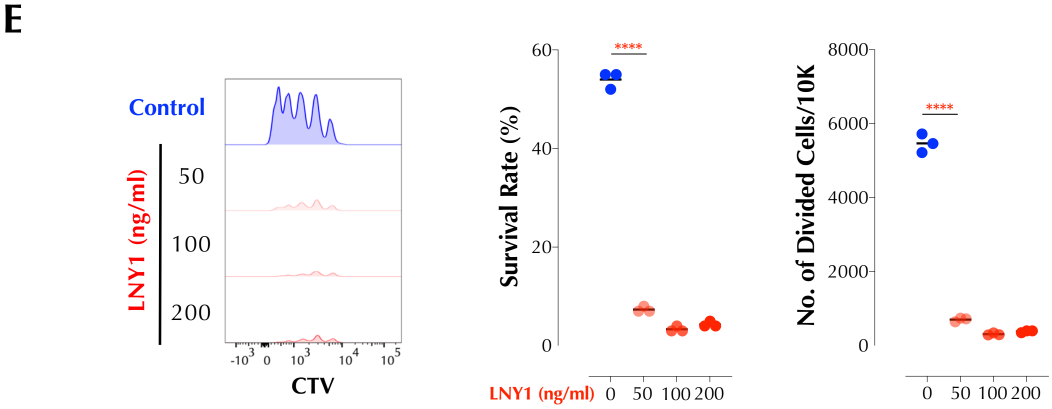

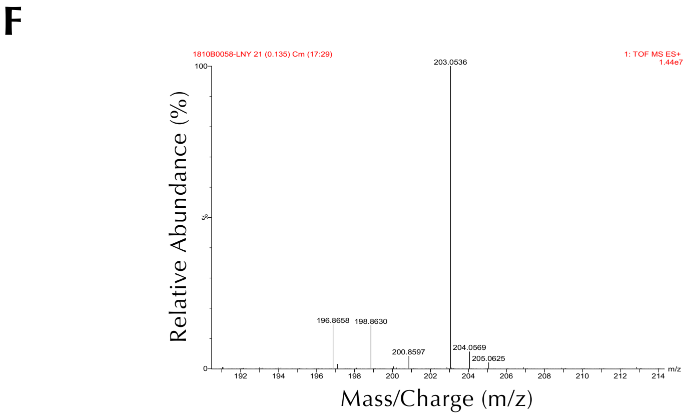

Supplementary Figure 1

G

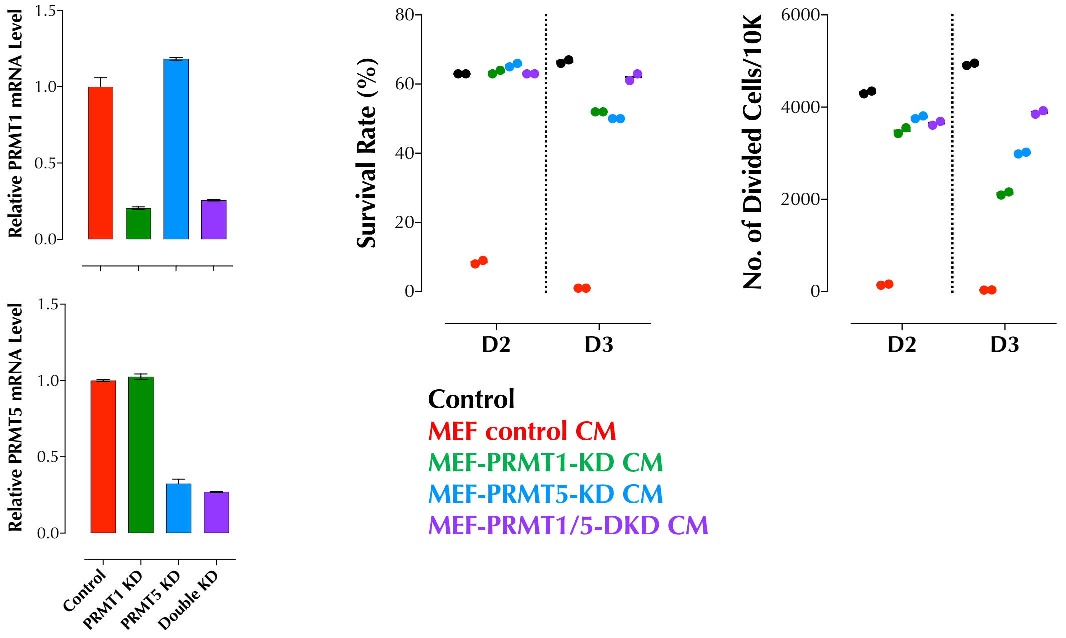

H

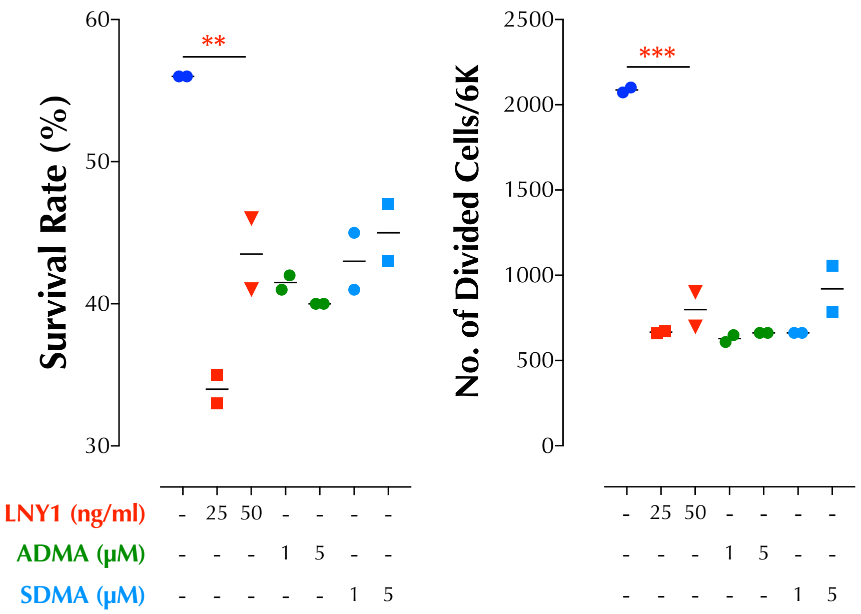

Supplementary Figure 1

I

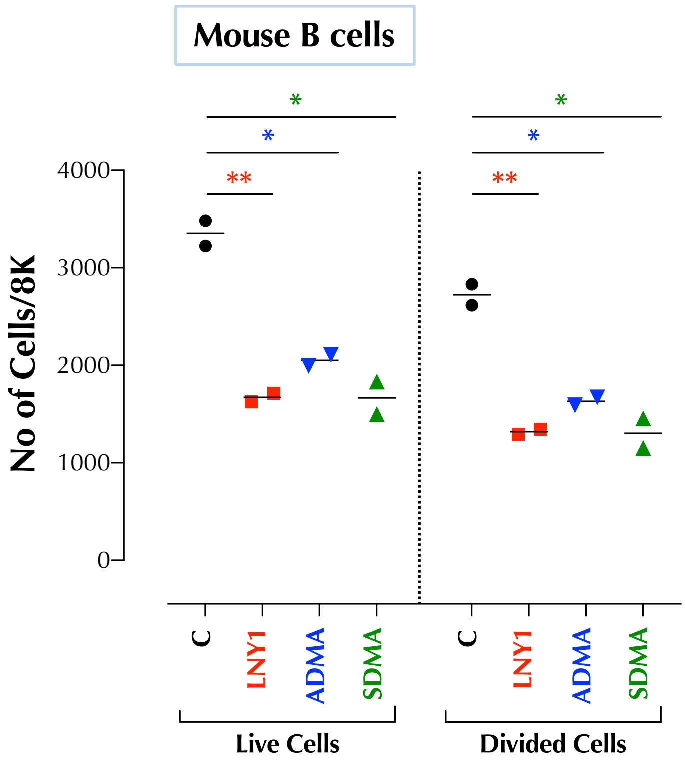

J

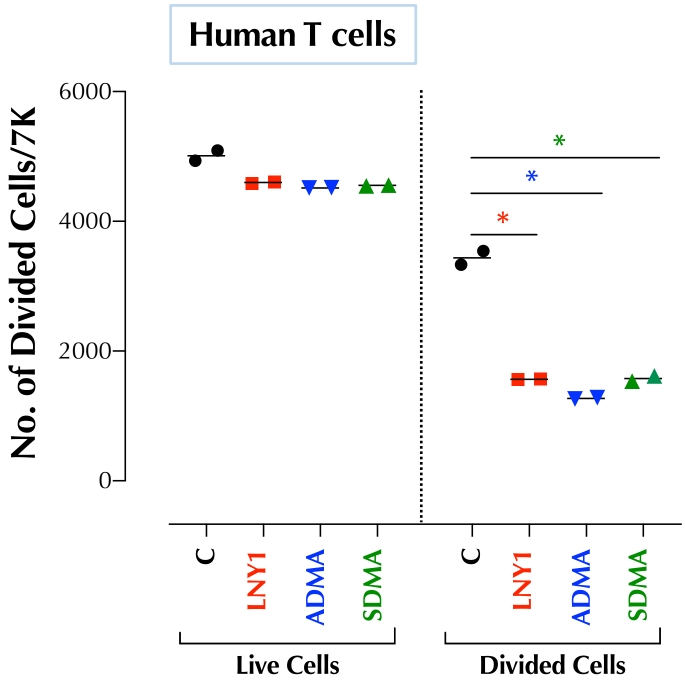

Supplementary Figure 1

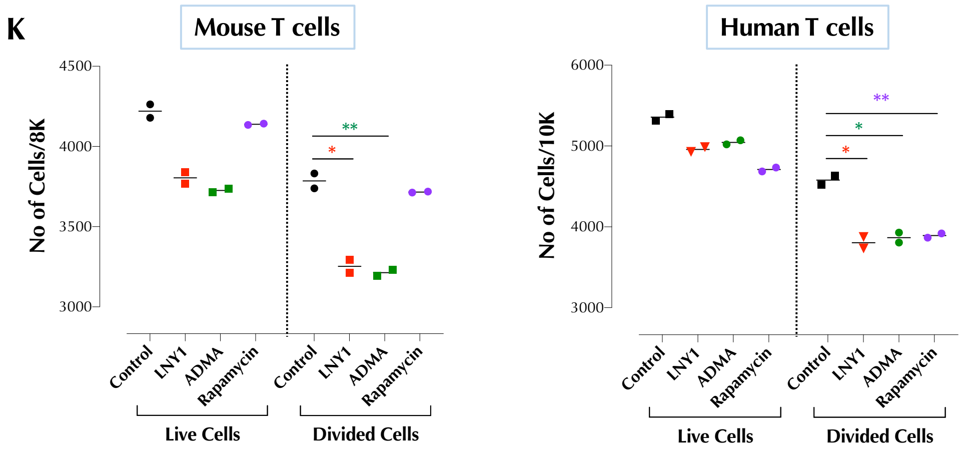

Supplementary Figure 2. ADMA and SDMA inhibit T cell proliferation via induction of mitochondrial ROS

A. LNY1 modifies the TCR-induced gene expression profile. Mouse splenic $CD3^+$ T cells were activated with or without LNY1 (100ng/ml) for 24h and the gene expression profiles were analyzed by RNAseq. PCA data show that LNY1 changed the TCR-induced gene expression pattern (left). While the pathways regulating metabolisms and cell cycle are down-regulated by LNY1, the pathways related to oxidative phosphorylation and chemokine/cytokine signaling were up-regulated (right). (n=3 independent biological samples). **B.** LNY1 decreases the TCR-induced mitochondrial membrane potential. Mouse splenic $CD3^+$ T cells were activated for 24h with the CD3/CD28 antibody-coated activation beads (cells:beads=4:1) with or without LNY1 (100ng/ml). The mitochondrial membrane potential was measured with TMRE by flow cytometry as described in the method. (n=3 independent biological samples, P<0.0001). **C.** LNY1 increases TCR-induced reactive oxygen species (ROS) in the mitochondria. Mouse splenic $CD3^+$ T cells were activated for 24h with the CD3/CD28 antibody-coated activation beads (cells:beads=4:1) with or without LNY1 (100ng/ml). The mitochondria-specific ROS was measured by flow cytometry. (n=3 independent biological samples, P=0.0001). **D.** LNY1 induces ROS in the mitochondria but not in the cytoplasm. Mouse splenic CD3+ T cells were incubated with LNY1 (100ng/ml. left) or tBHP (100μM, right) for the indicated time periods, and the mitochondrial or cytosolic ROS was measured with CellROX Green or CellROX Deep Red, respectively, as described in the method (the statistical significance was analyzed by 2-way ANOVA). **E.** LNY1 enhances TCR-induced mitochondrial ROS**.** To assess the effects of LNY1 on TCR-induced mitochondrial ROS, mouse splenic $CD3^+$ T cells were activated with the CD3/CD28 antibody-coated activation beads (cells:beads=4:1) with or without LNY1 (100ng/ml) for the indicated time periods and the mitochondrial ROS was measure by flow cytometry. (n=3 independent biological samples, ***<0.001, ****<0.0001). **F.** LNY1 induces mitochondrial ROS in mouse T cells. Upper panel: To detect ROS in live cells, freshly isolated mouse splenic T cells were incubated with LNY1 (100ng/ml) or nothing for 30min with or without MitoQ (100nM) in the presence of Hoechst 33342 (1μg/ml) and the images the live cells were acquired by confocal microscopy. Hoechst 33342 positive cells represent the dying cells that lost membrane integrity. Lower panel: To localize the mitochondria, T cells were incubated with MitoTracker Deep Red and Hoechst 33342 (1μg/ml) for 30min and the images the live cells were acquired by confocal microscopy. **G.**

The iNOS inhibitor N6-(1-iminoethyl)-L-lysine (NIL) or the antioxidants NAC and MitoQ does not significantly affect T cell proliferation. Mouse splenic $CD3^+$ T cells were activated with the CD3/CD28 antibody-coated activation beads (mouse T cells, cells:beads=4:1) with the indicated chemicals. Cell survival and proliferation were analyzed after 72h post stimulation. (n = 4, no statistical significance). **H.** The iNOS inhibitor NIL does not induce ROS in the mitochondria. Real-time mitochondrial ROS induced by ADMA or NIL was measured by flow cytometry.

Supplementary Figure 2

A

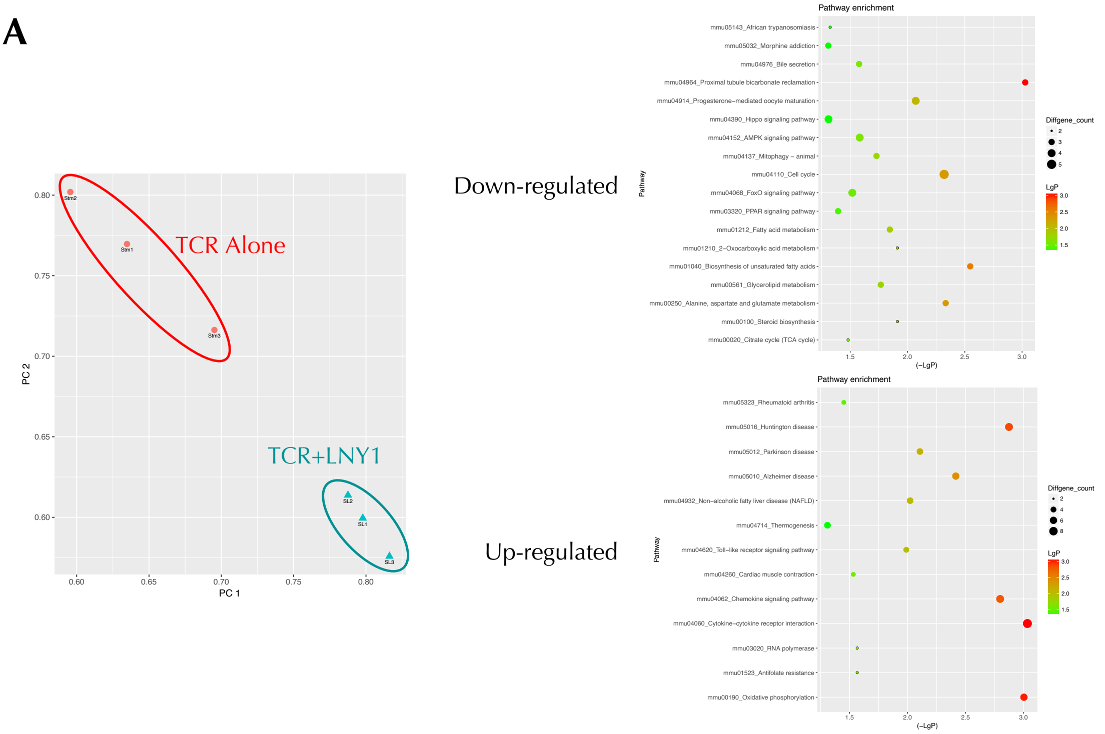

B

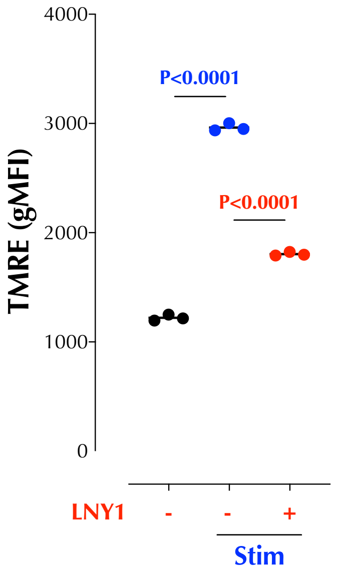

C

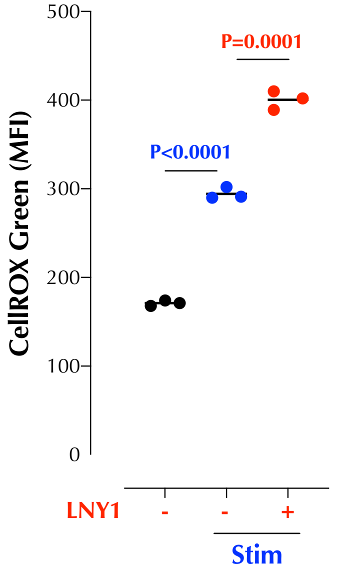

Supplementary Figure 2

D

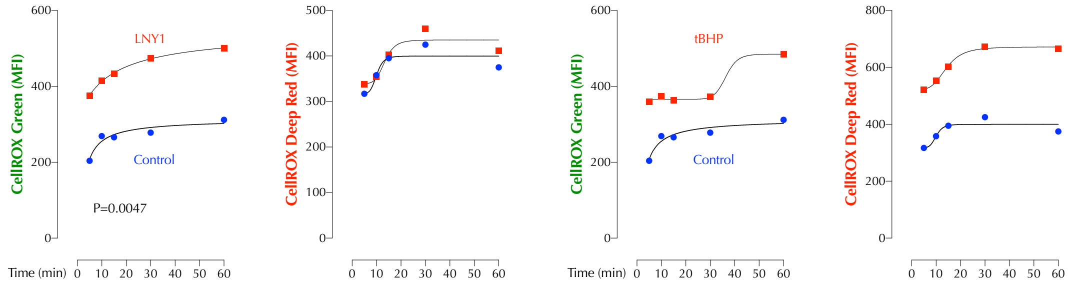

E

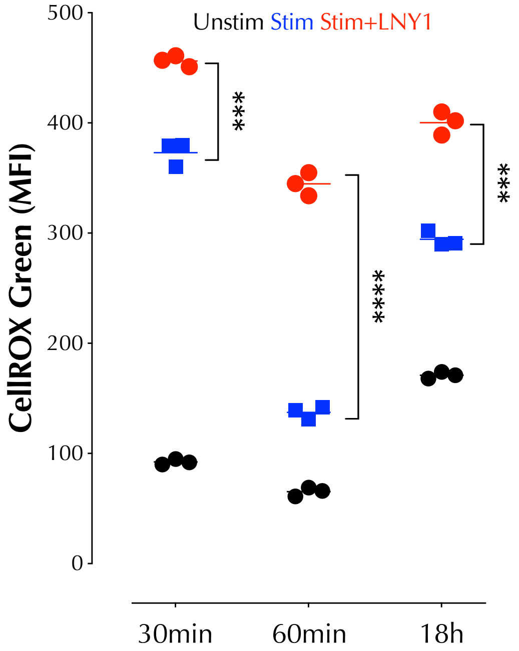

Supplementary Figure 2

F

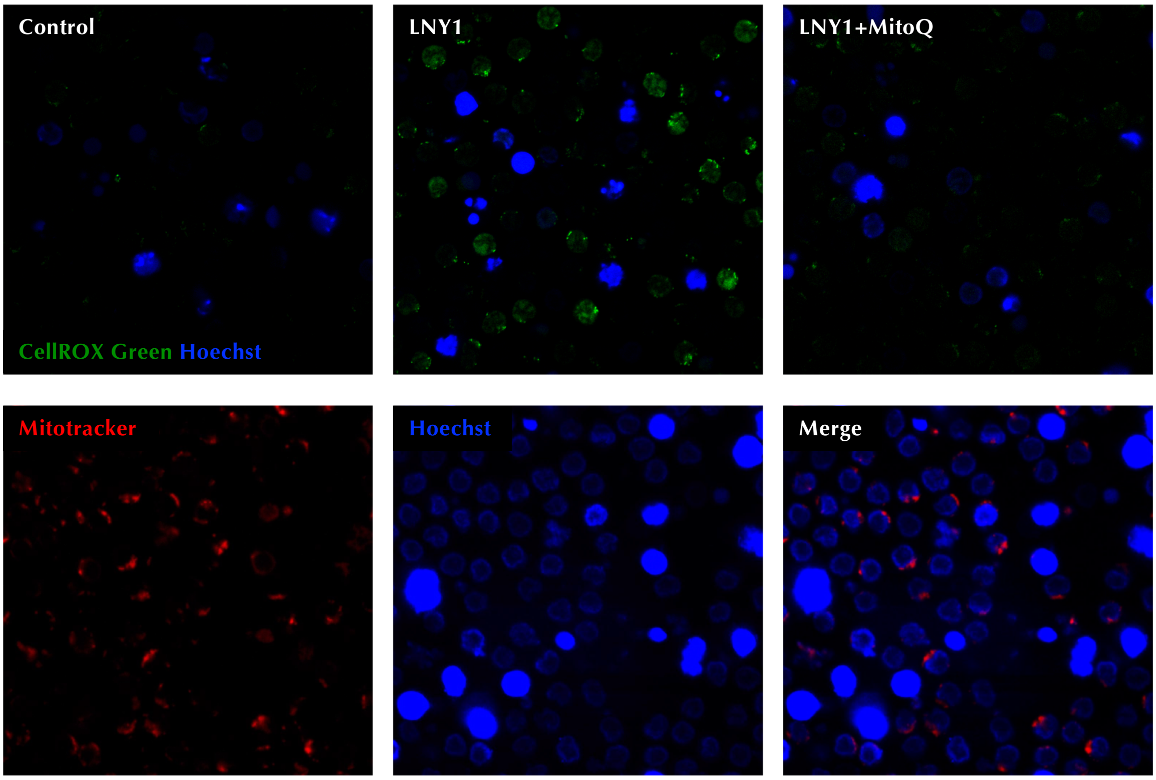

Supplementary Figure 2

G

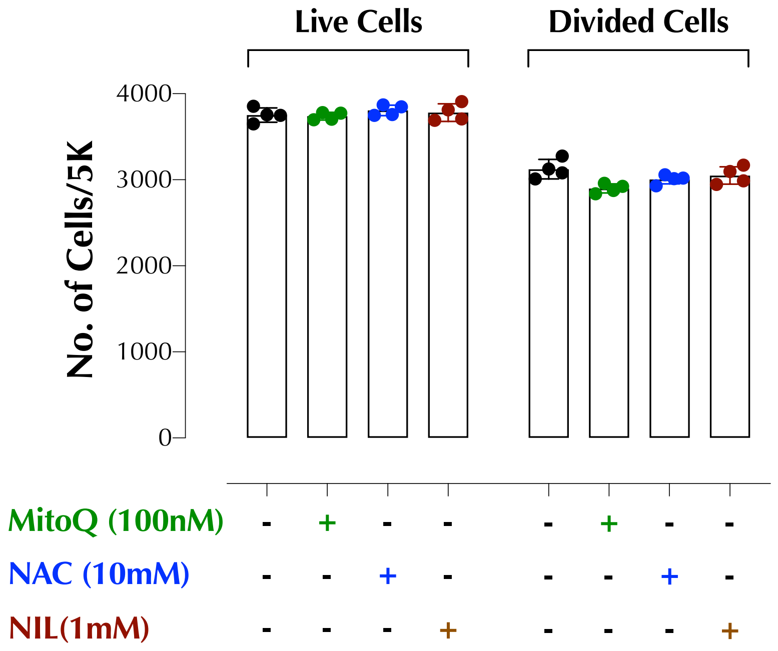

H

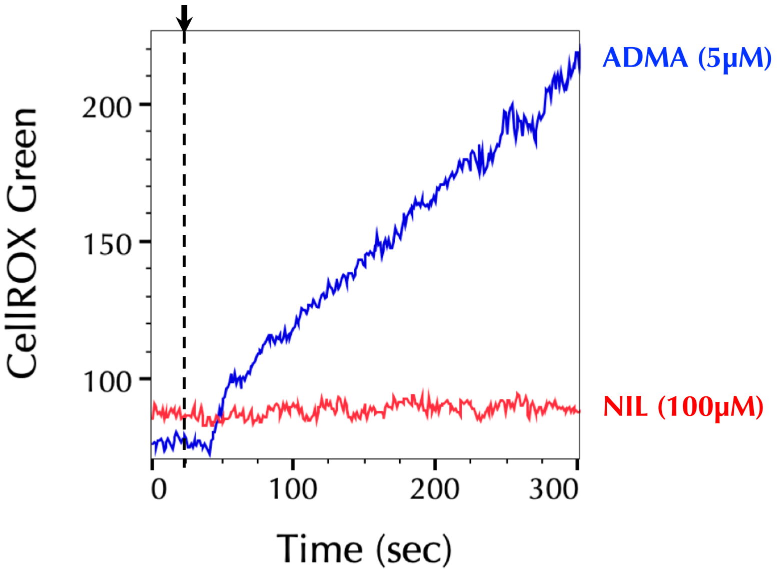

Supplementary Figure 3. ADMA and SDMA must be metabolized by AGXT2 to DMGV to induce ROS

A. Schematic representation of the ADMA and SDMA metabolic pathways. **B.** L-citrulline neither induces ROS nor significantly affects T cell survival or proliferation. (Top) Mouse splenic $CD3^+$ T cells loaded with CellROX Green were treated (arrow) with DMGV (5µM) or L-citrulline (10µM) and real-time ROS induction was measured by flow cytometry. (Bottom) Mouse splenic $CD3^+$ T cells were activated with or without L-citrulline (10µM). Survival and proliferation were measured by flow cytometry 48h and 72h after activation. (n=2 independent biological samples) **C.** AGXT2 overexpression (OE) significantly enhances mitochondrial ROS induction by ADMA and SDMA. AGXT2 was overexpressed in mouse splenic $CD3^+$ T cells and was confirmed by qPCR. Mitochondrial ROS of control and OE cells were measured after 30min incubation with ADMA (10µM), SDMA (10µM), or DMGV (10µM). (n=2 independent biological samples, **<0.01, ***<0.001). **D**. AGXT2 OE inhibits T cell survival and proliferation. Control or AGXT2-OE mouse splenic $CD3^+$T cells were activated, and cell survival and proliferation were measured after 48h or 72h by flow cytometry (left and upper right). Control and AGXT2-OE mouse splenic $CD3^+$ T cells were activated, with LNY1 (100ng/ml), ADMA (10µM) or Rapamycin (100nM), and cell survival and proliferation were measured after 72h (lower right). (n=2 biological independent samples, **<0.01, ***<0.001) **E.** AGXT2 is localized in the mitochondria. The human colonic carcinoma HCT-8 cells were transfected with mouse AGXT2. After 24h, cells were stained with anti-AGXT2 antibody followed by anti-rabbit IgG-Alexa Fluor 488 and anti-COX IV-Alexa Fluor 647, together with DAPI and images were taken with a confocal microscope as described in the method section. **F.** DMGV induces ROS in mitochondria in all PBMCs. Human PBMCs were isolated from human peripheral blood and then incubated with different concentrations of DMGV or tBHP. Mitochondrial or cytosolic ROS was measured as described in the method section. PBMCs were stained with anti-CD3 and anti-CD19 antibodies before analysis. Monocytes were identified by the cell size (FSC-A). (n=2 independent biological samples) **G.** DMGV does not induce cell death in human PBMCs. Human PBMCs were incubated with different concentrations of DMGV or tBHP. Cell survival was measured by flow cytometry after 24h. PBMCs were stained with anti-CD3 and anti-CD19 antibodies before analysis. Monocytes were identified by the cell size. (n=2 independent biological samples) **H.** DMGV causes cell death in TCR-activated Jurkat T cells via mitochondrial ROS induction. Jurkat T cells were

treated as indicated for 24h, and cell survival was measured by flow cytometry. (n=2 independent biological samples) **I.** ADMA, SDMA, and DMGV block cell cycle progression from S to G0-G1 phase. CTV-labeled mouse T cells were stimulated with the indicated compounds for 3 days and the cell cycle was measured according to the manufacturer's instruction (Yishan Biology, Shanghai, China). The results represent two similar experiments.

Supplementary Figure 3

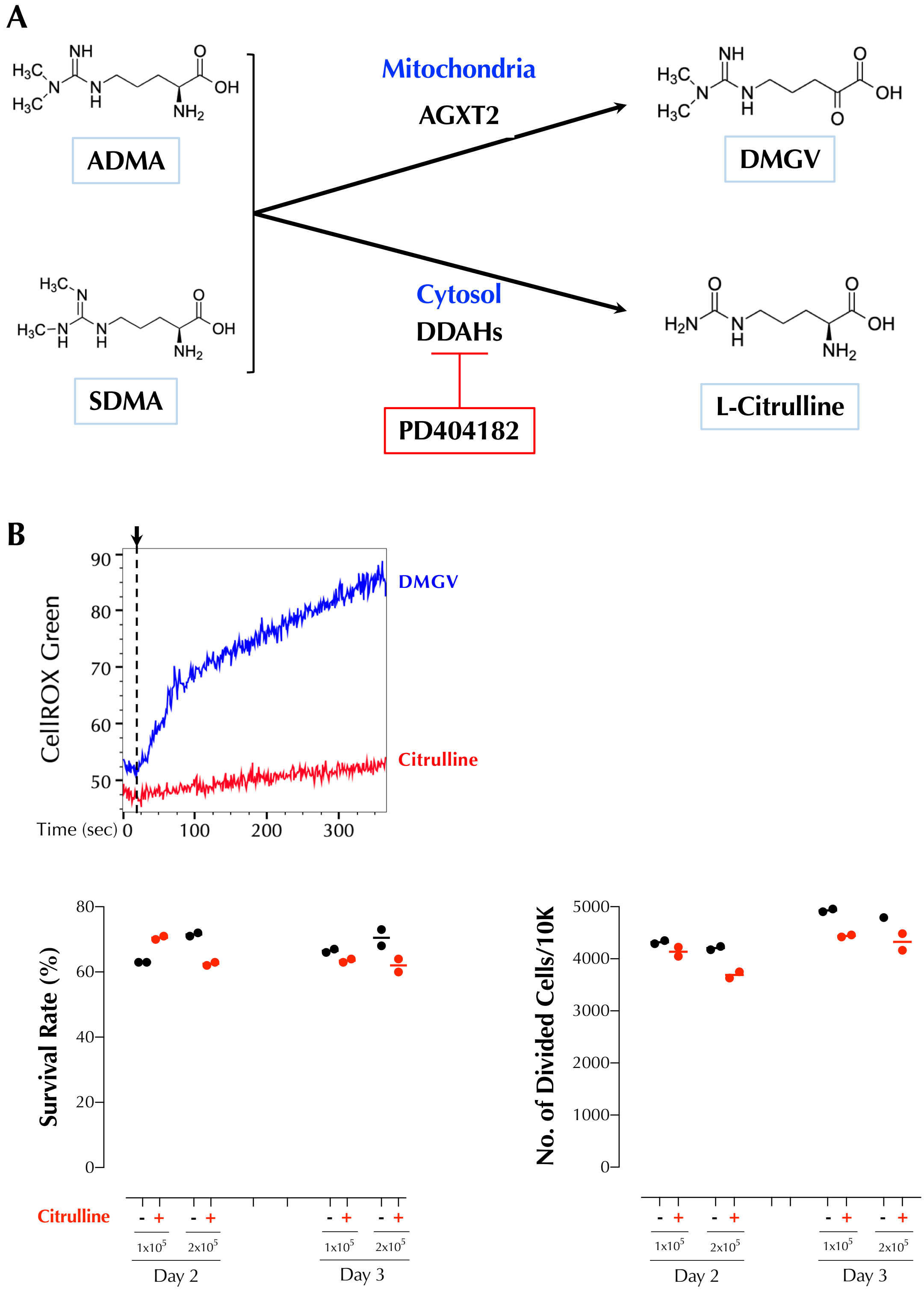

Supplementary Figure 3

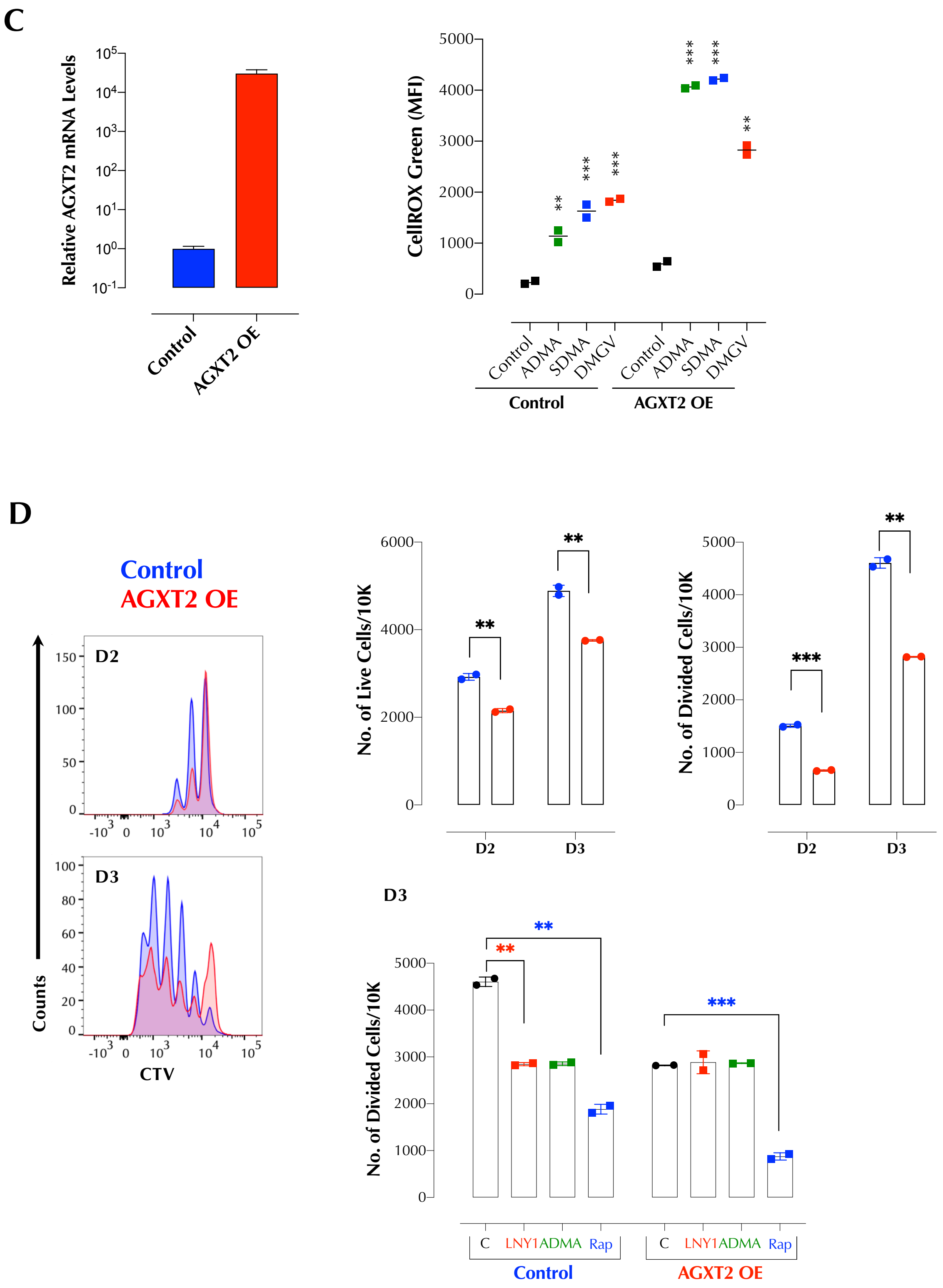

Supplementary Figure 3

E HCT-8

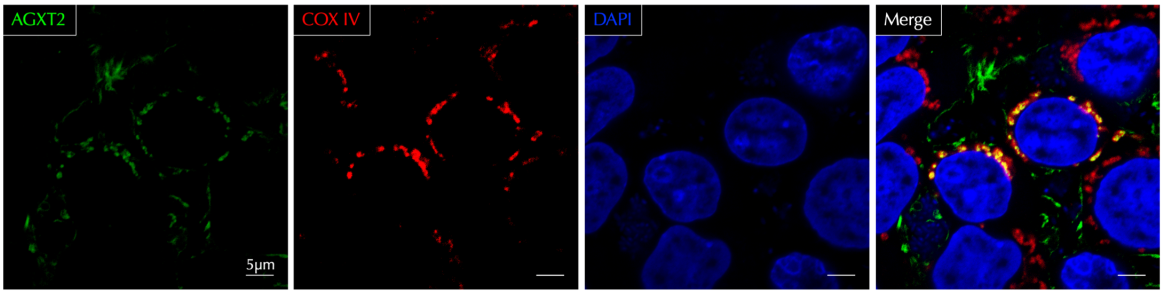

F

Human PBMCs (1h)

Human PBMCs (24h)

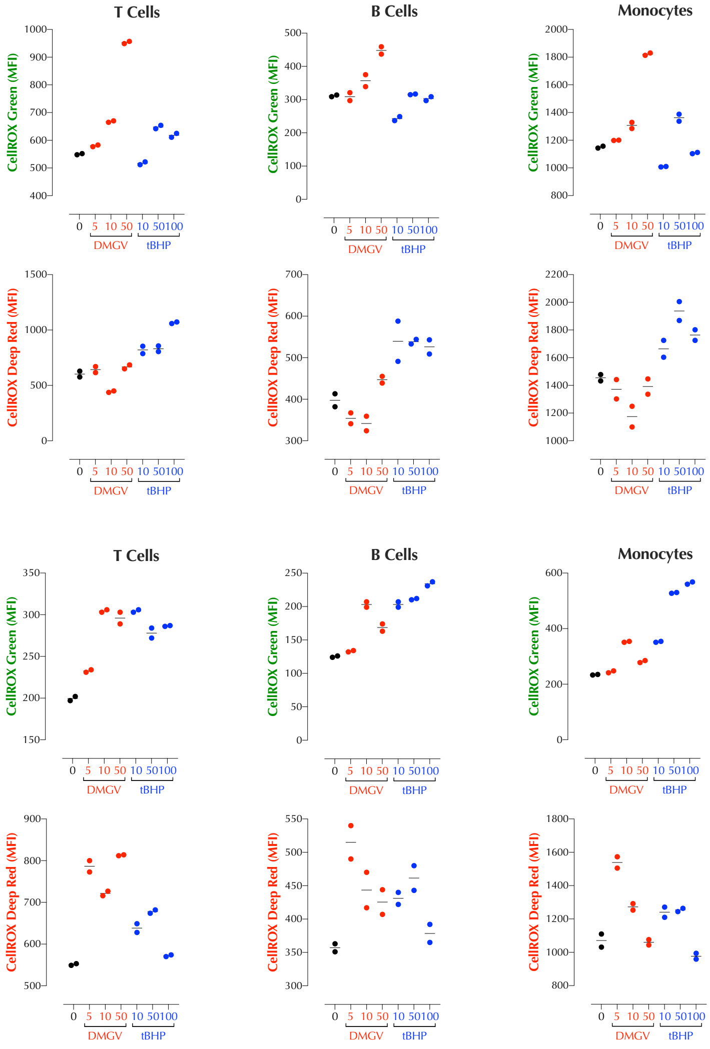

Supplementary Figure 3

G Human PBMCs (24h)

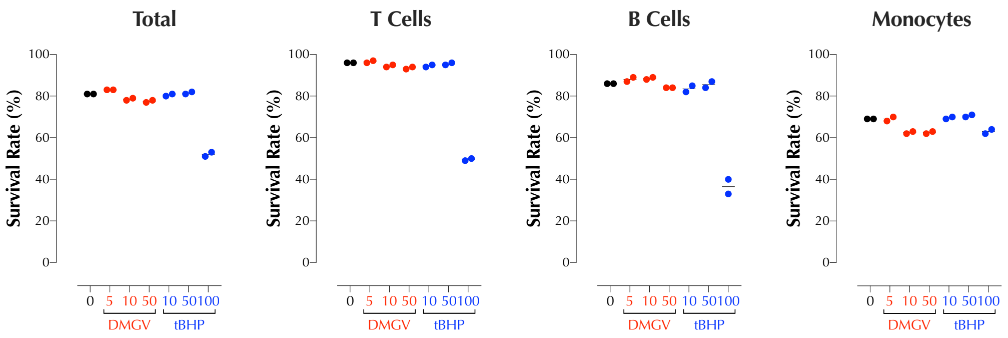

H Jurkat (24h)

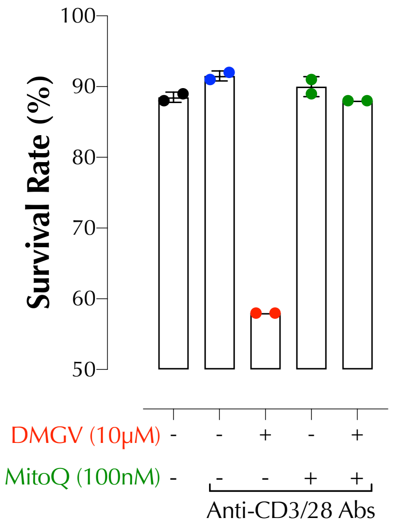

Supplementary Figure 3

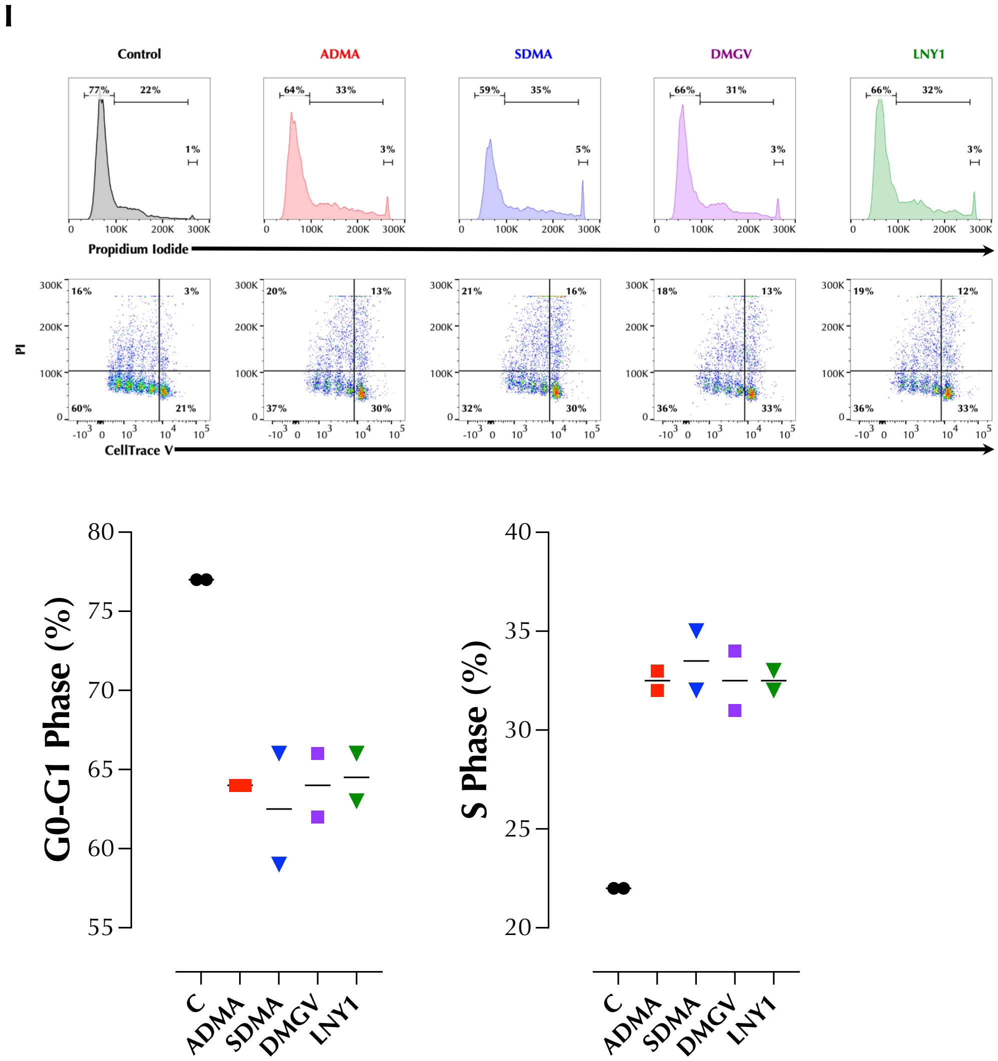

Supplementary Figure 4. AGXT2 heterozygotes are hypomorphs.

A. AGXT2 KO strategy in mice. The details are described in the method section. **B**. A representative genotyping analysis by PCR. **C. D.** AGXT2$^{+/-}$ mice are hypomorphs. The level of AGXT2 mRNA in total thymocytes or splenocytes of AGXT2$^{+/-}$ or AGXT2$^{-/-}$ mice was measure by qPCR. (Each dot represents a single mouse.) **E.** T cell development is not affected by the decreased expression of AGXT2 mRNA. Left: a representative flow cytometry analysis of thymocytes and splenocytes. Right: cumulative cellularity analysis. (Each dot represents a single mouse, DN: double negative, DP: double positive) **F.** AGXT2 mRNA is induced by T cell activation. Splenic T cells were activated for 24h and the levels of AGXT2 and DDAH1 were measured by qPCR. **G.** Mitochondrial ROS induced by DMGV is a rheostat of T cell survival. Splenic AGXT2$^{+/+}$ or AGXT2$^{+/-}$ T cells were stimulated as indicated and the survival rate was measure at 72h by flow cytometry. **H**. Human peripheral CD3^{+} T cells were transfected with AGXT2 siRNA and stimulated the next day as indicated. The survival rate was monitored daily up to 7 days and the cell death rate by 7AAD staining by flow cytometry. **I**. AGXT2 is essential for survival of CD4^{+} effector T cells but not Treg. Naïve CD4^{+} cells were purified and activated under the indicated Th differentiation conditions as described in the method. The number of live cells were counted by flow cytometry after 72h. The data is the representative of 2 separate experiments. **J**. The AGXT2-DMGV axis maintains the survival of Jurkat T cells. Jurkat T cells were electroporated with C or AGXT2 siRNA, cultured with or without DMGV (5µM) or TGFβ (15ng/ml), and the survival rate was measured on day 5. **K.** TGFβ cannot rescue AGXT2$^{+/-}$ T cells in the absence of glutamine. WT and AGXT2$^{+/-}$ T cells were stimulated as indicated (5µM DMGV, 15ng/ml TGFβ, 2mM pyruvate) for 3 days in glutamine-free RPMI and survival and proliferation were measured by flow cytometry. The data represent 3 similar experimental results. **L.** Glucose uptake is not significantly affected in AGXT2$^{+/-}$ T cells. WT and AGXT2$^{+/-}$ T cells were stimulated up to 2 days, and glucose uptake was measured at the indicated time point with 2-NBGD according to the manufacturer's instruction. The data represent 2 similar experimental results.

Supplementary Figure 4

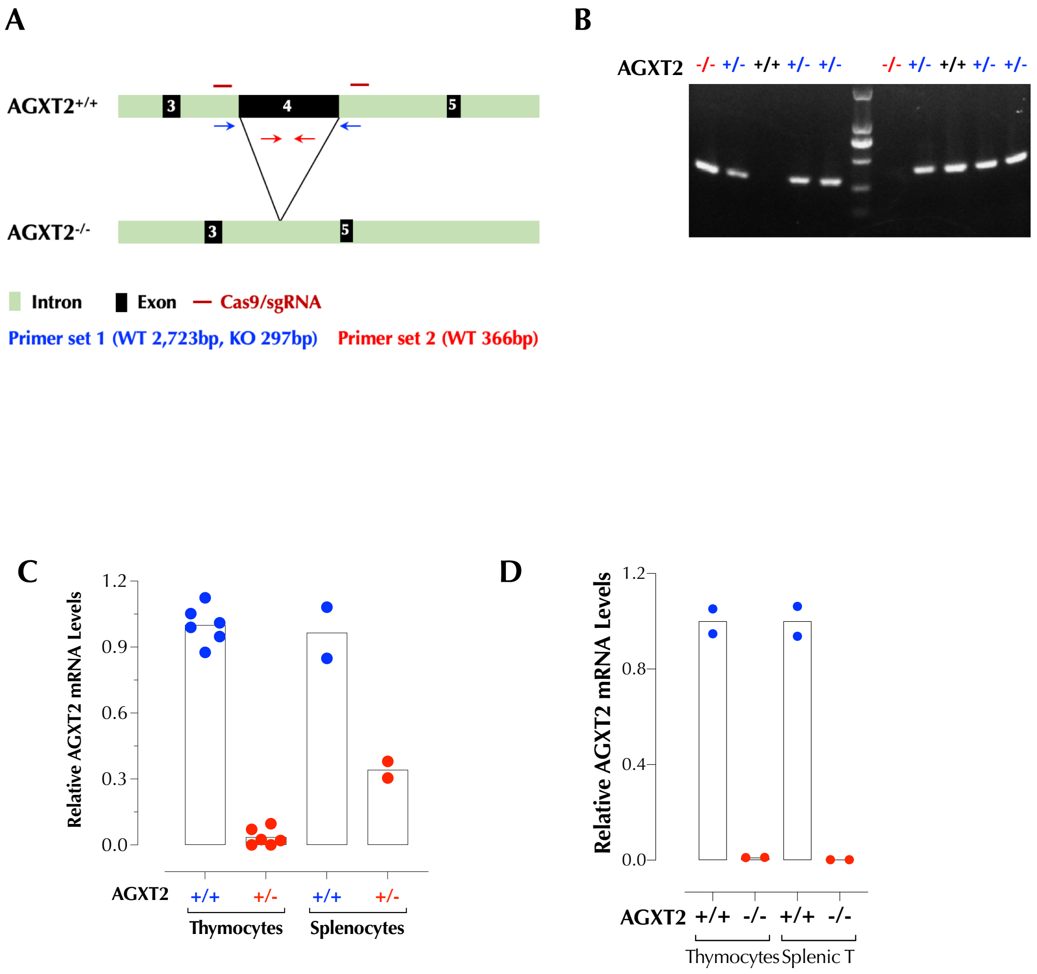

Supplementary Figure 4

E

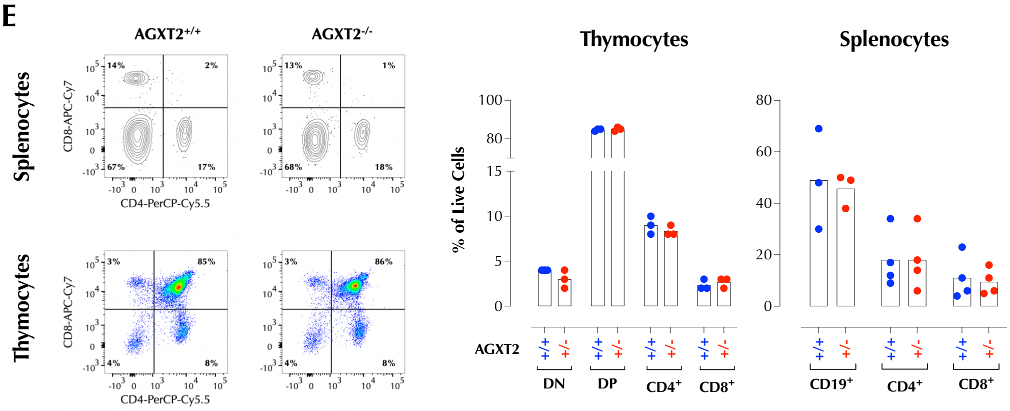

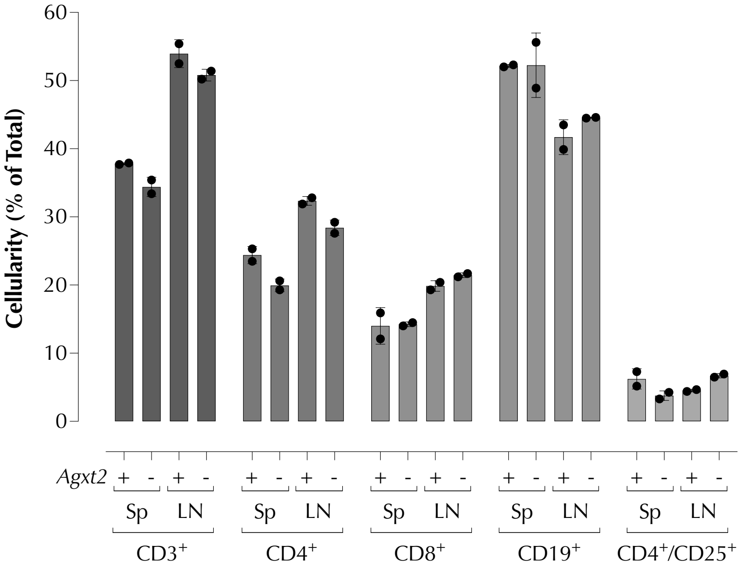

+: +/+, -: -/-,   Sp: Spleens, LN: Lymph Nodes

Supplementary Figure 4

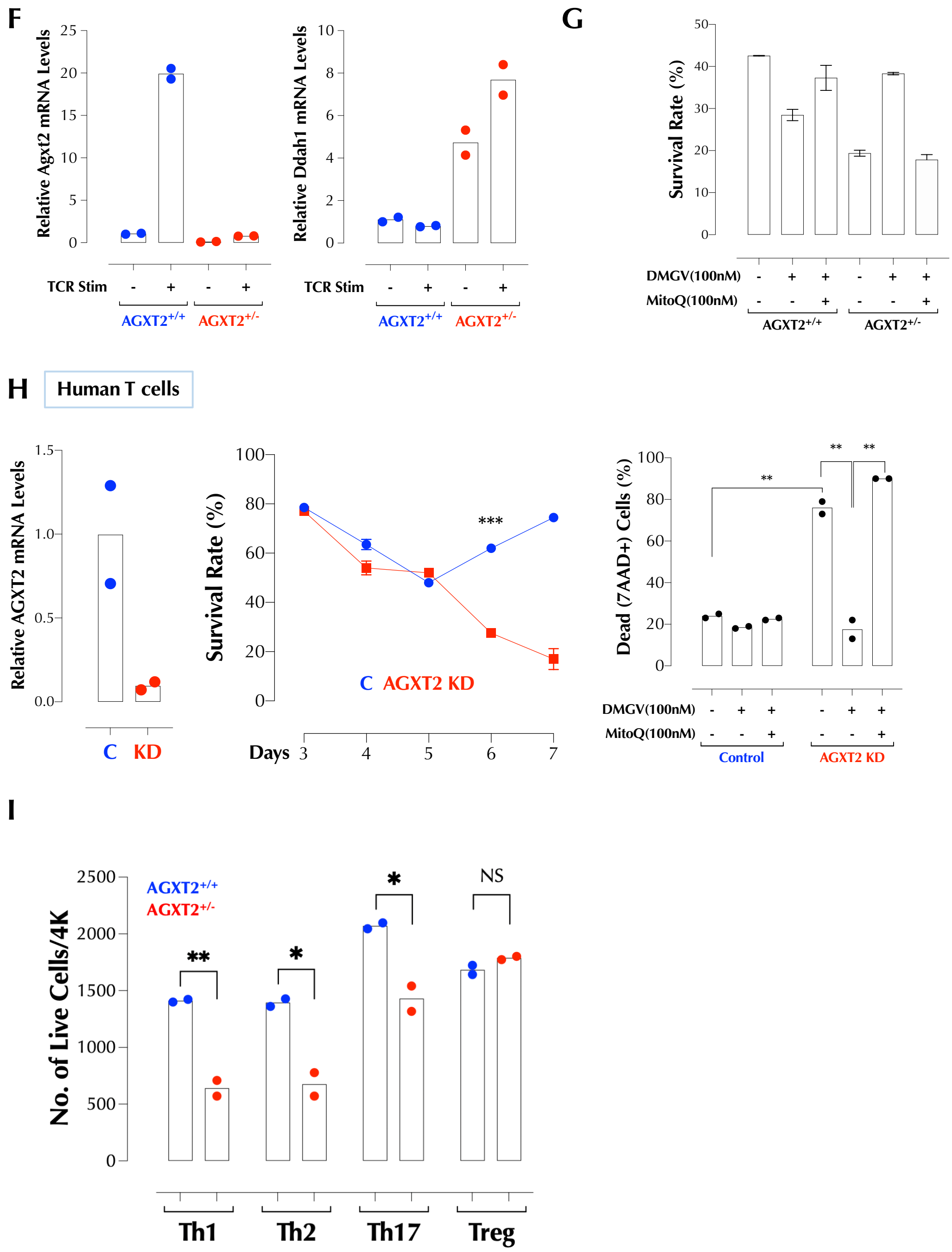

Supplementary Figure 4

J

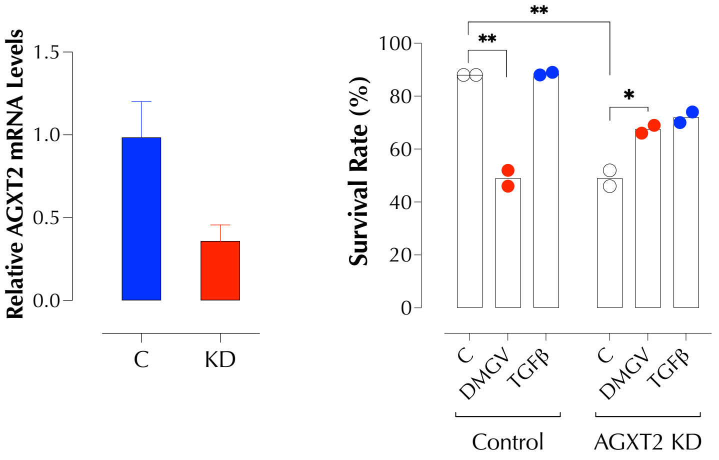

K

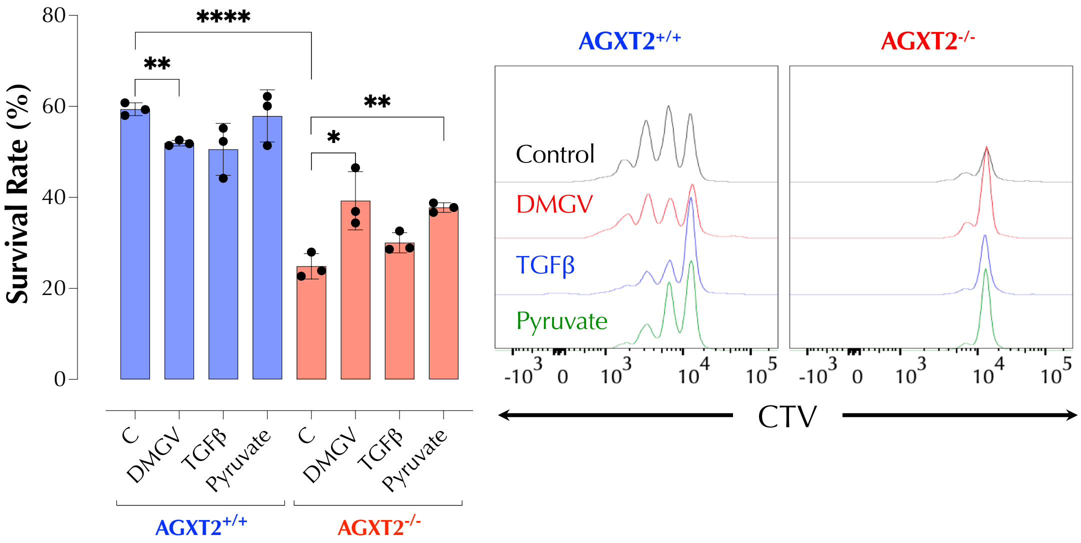

Supplementary Figure 4

L

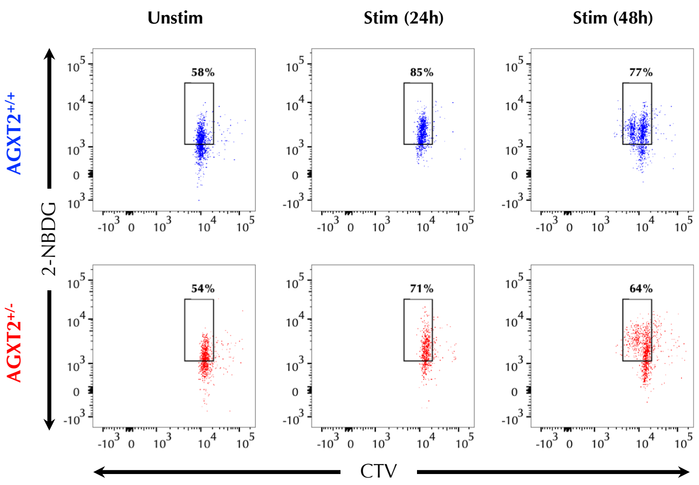

Supplementary Figure 5. DMGV induces calcium efflux mainly via RyR1

A. DMGV induces mitochondrial ROS in Jurkat cells. Jurkat cells were loaded with either MitoSOX or CellROX Green for 15min according to the manufacturer's instruction and ROS induction by DMGV was measured by flow cytometry. The line graph was generated using the FlowJo kinetics function and the mean fluorescence was smoothed with the moving average. **B.** DMGV, but not tBHP, induces ROS in the mitochondria of Jurkat cells. DMGV (5µM) or tBHP (1mM) was added at the indicated time point by an arrow (20 seconds) and MitoQ (100nM) was added 30min before the assay. **C-D.** DMGV induces ROS in a cell-free system (or a permeabilized cell system). Jurkat cells (**C**) or mouse splenic T cells (**D**) were loaded with TMRE or CellROX Green and treated for 2min with digitonin (60µg/ml for Jurkat and 100µg/ml for mouse T cells) before TMRE or ROS measurement by flow cytometry. **E.** The RyR inhibitor ryanodine inhibits DMGV-induced ROS production. Jurkat cells were treated with the indicated amount of ryanodine and CellROX Green for 30min and ROS induction was measured by flow cytometry. **F.** DMGV induces calcium efflux mainly via RyR1. RyR1 or ITPR1 was knocked out using the Crispr/Cas9 system and calcium efflux (bottom left) and ROS induction (bottom right) by DMGV was measured by flow cytometry. **G.** RyR1 KD compromises T cell survival and proliferation. Control and RyR1-KD mouse T cells were stimulated with the indicated compounds for 2 days, and the survival and cell division were measured by flow cytometry,

Supplementary Figure 5

A Jurkat T cells

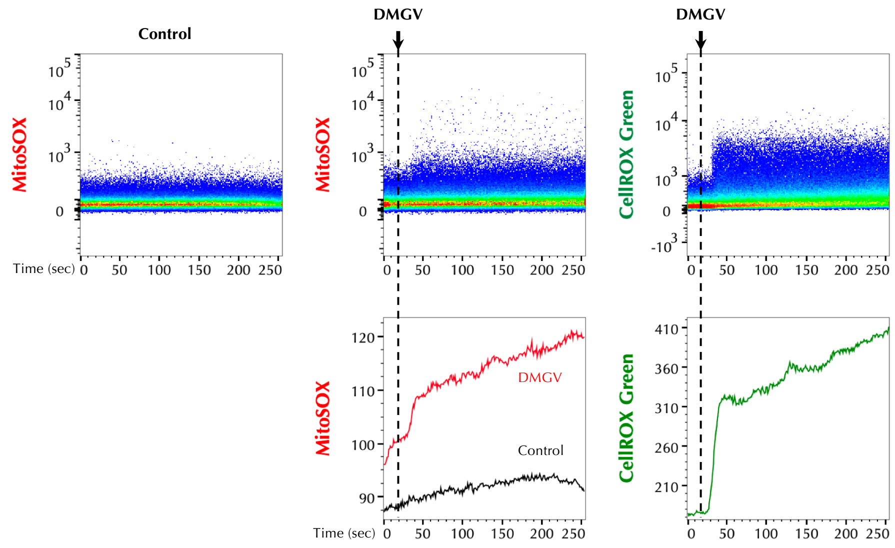

B Jurkat T cells

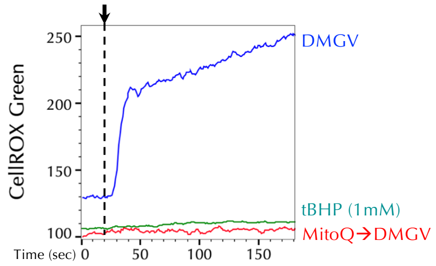

Supplementary Figure 5

C

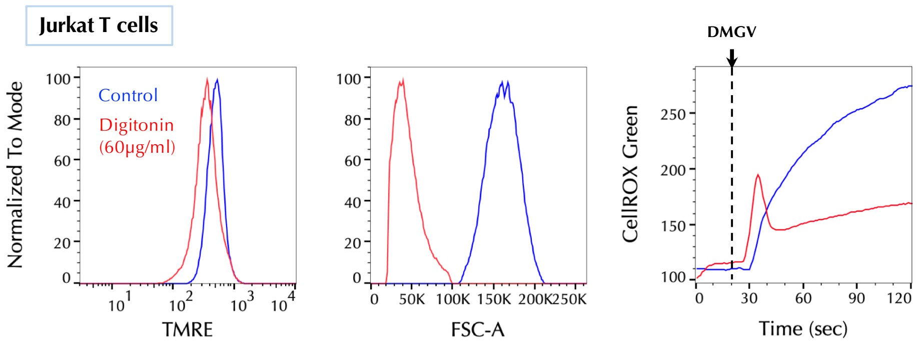

D

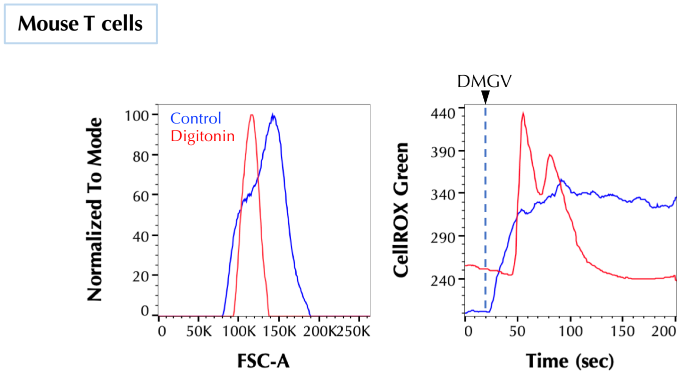

Supplementary Figure 5

E **Jurkat T cells**

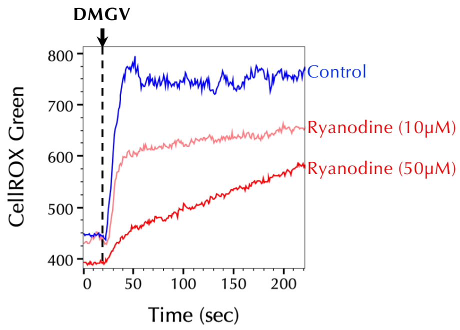

F **Jurkat T cells**

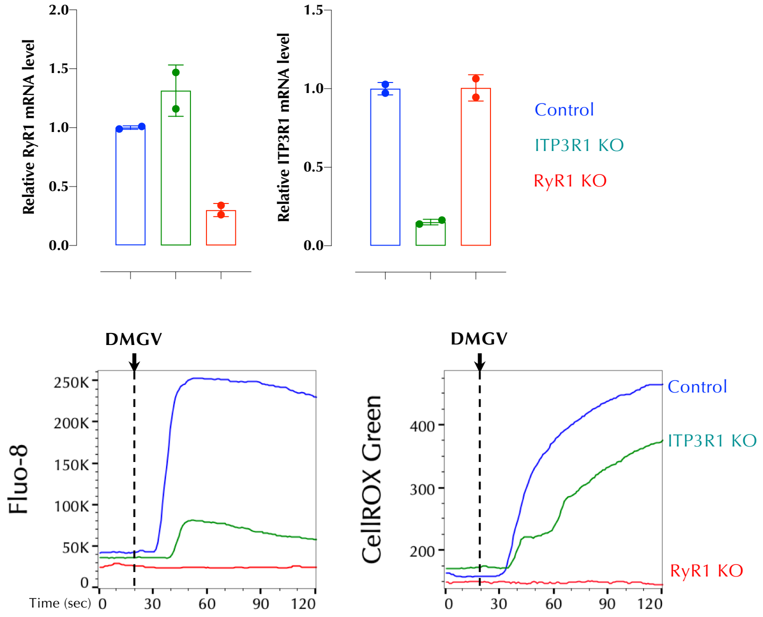

Supplementary Figure 5

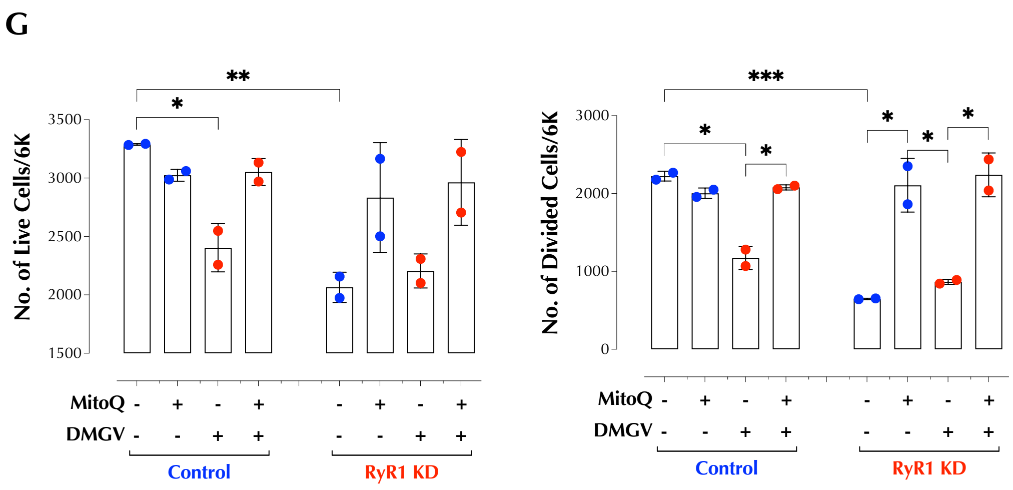

Supplementary Figure 6. Ile127 is essential for DMGV to activate MCU

A. The structural difference between ADMA and DMGV indicates that the highlighted carbonyl group may be essential for the biological activity of DMGV. **B.** MCU-WT, $MCU^{\Delta Ile127}$, $MCU^{\Delta Val135}$, and $MCU^{\Delta Ile127/Val135}$ are localized in the mitochondria. Jurkat cells were transfected with the HA-tagged MCU constructs, and stained with anti-HA antibody followed by anti-rabbit IgG-Alexa Fluor 555, which was followed by staining the mitochondrial protein COX IV-Alexa Fluor 647.

Supplementary Figure 6

A

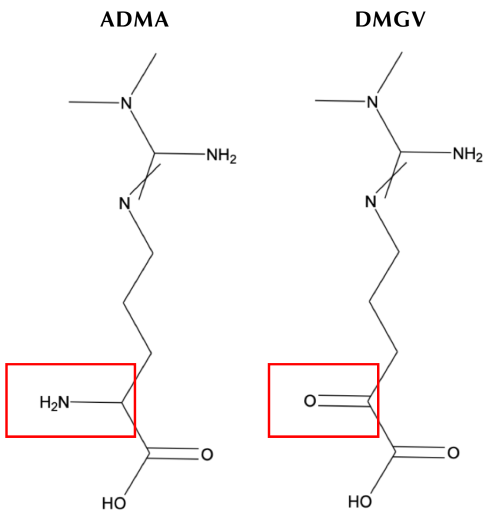

B

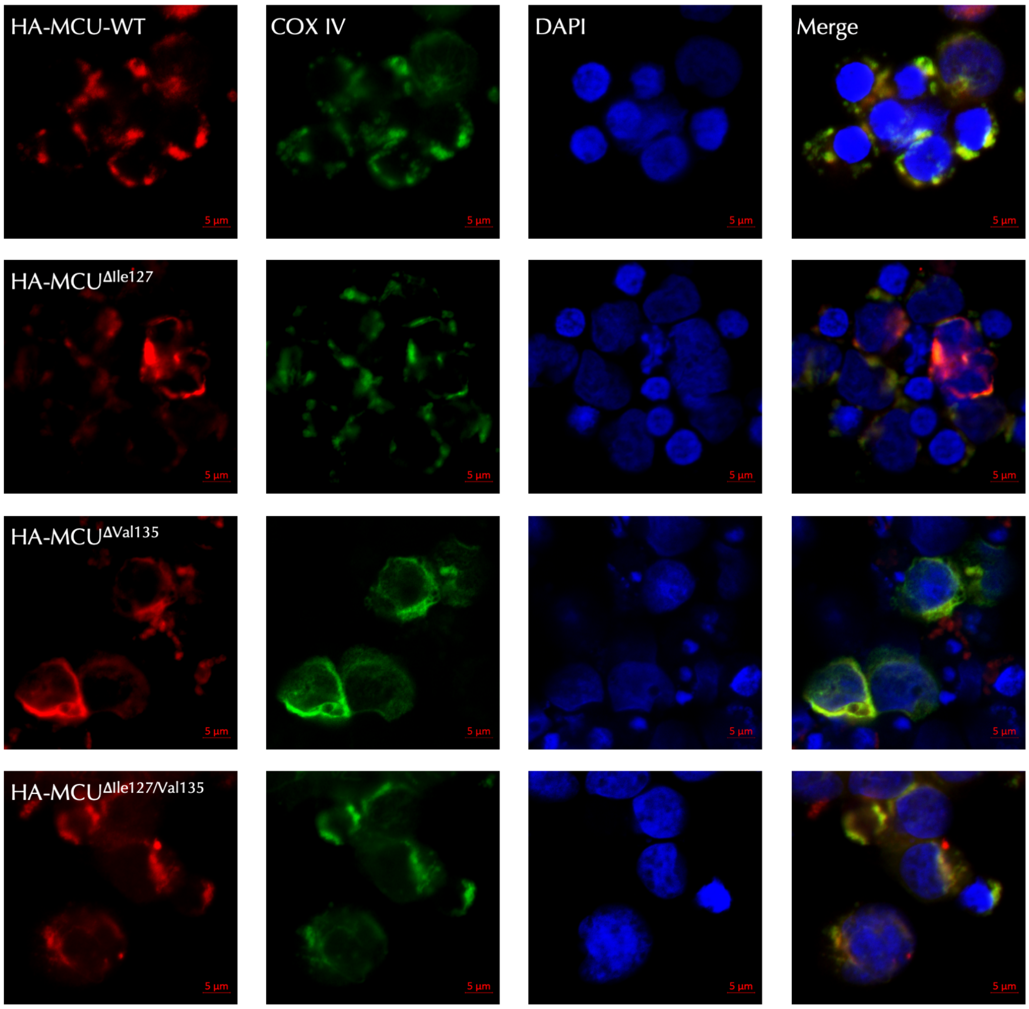

Supplementary Figure 7. DMGV induces mitochondrial calcium and ROS while TCR, Thapsigargin, and ionomycin induces calcium flux to the mitochondria without ROS induction.

A. Jurkat cells stably expressing Mito-GEM-GECO were treated with anti-human CD3 Ab (5µg/ml), DMGV (5µM), or ionomycin (500ng/ml) and the calcium influx was measured by flow cytometry. **B.** TCR, Thapsigargin, or ionomycin does not induce mitochondrial ROS in Jurkat cells. Jurkat cells were treated with anti-human CD3 Ab (5µg/ml), DMGV (5µM), Thapsigargin (1µM), or ionomycin (500ng/ml) and the ROS induction was measured by flow cytometry. **C.** DMGV inhibits OxPhos induced by TCR stimulation in primary mouse and human T cells. Seahorse assay was performed according to manufacturer's instruction in a 24-well format. Anti-CD3 Ab (5µg/ml) with or without DMGV (5µM) was injected at 10min, and MitoQ (100nM) was added before the start. Oligomycin (Oligo, 2µM), FCCP (1.5µM), and rotenone/antimycin A (R/A, 0.5µM) were added at the indicated time points. **D.** Coro-Na Green is localized in the mitochondria. Jurkat T cells were loaded with Coro-Na Green as described [1]. Briefly, Jurkat T cells were washed with HBSS containing Ca^{2+}, Mg^{2+}, and glucose (4g/L), incubated for 1h with 10µM Coro-Na Green AM, washed again, and incubated for a further hour with MitoTracker Deep Red (DR) in the same buffer. The cells were washed and fixed with 4% paraformaldehyde for 15min at room temperature. The cells were cyto-spun and mounted for confocal microscopy. **E.** Combination of anti-CD3 and CD28 Abs or PMA and ionomycin induces mitochondrial ROS. Jurkat cells were stimulated with anti-CD3 Ab (5µg/ml), CD28 Ab (5µg/ml), PMA (50ng/ml), and ionomycin (500ng/ml) alone or in combination as indicated and ROS was measure by flow cytometry. **F.** PMA enhances ionomycin-induced calcium influx, and the combination of PMA and ionomycin induces Na^+ influx to the mitochondria. Calcium influx to cytosol and mitochondria, and Na^+ influx to the mitochondria in Jurkat T cells were measured as described in the method by flow cytometry.

Supplementary Figure 7

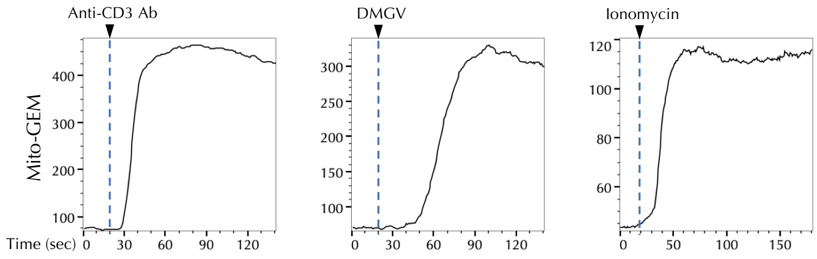

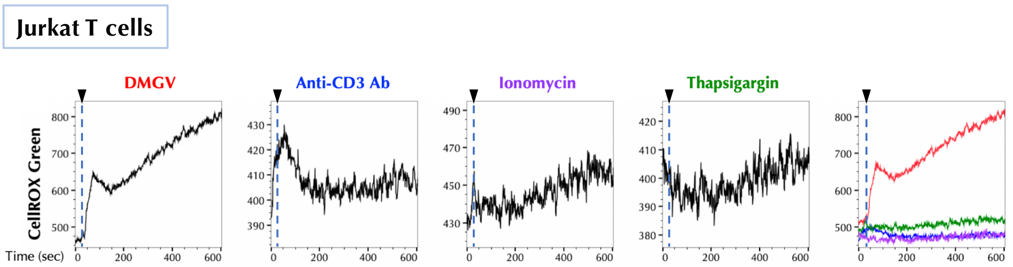

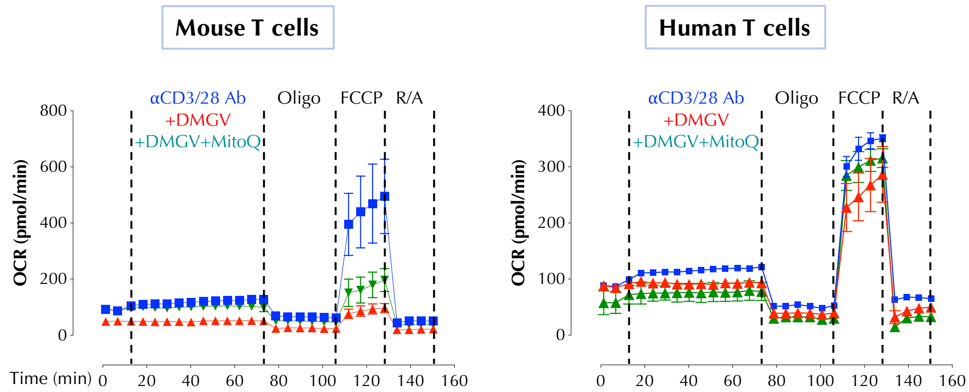

Supplementary Figure 7

D

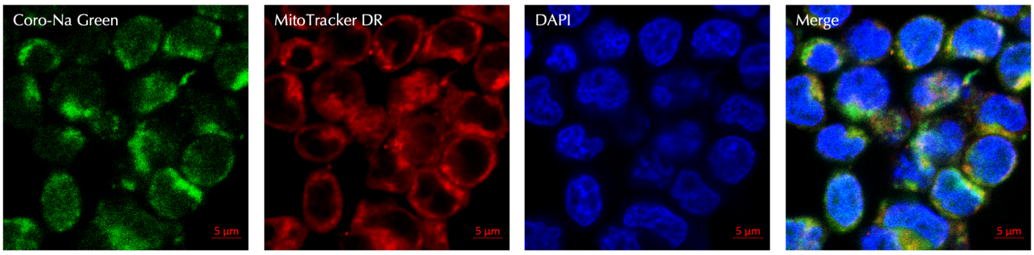

E

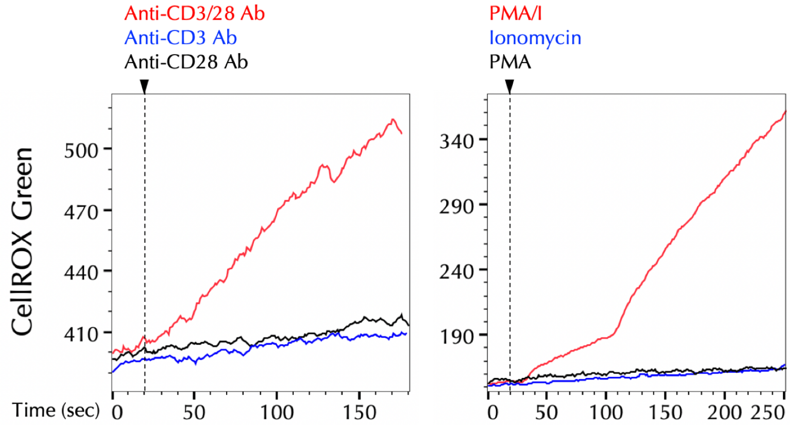

F

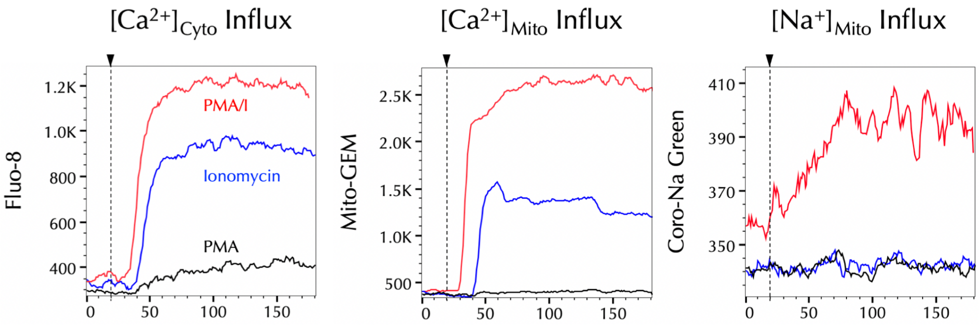

Supplement: Supplementary file 1 [file DataSheet_1.zip › Supplemental Figures 1-7.pdf]
