# Supplementary material for: DMGV Is a Rheostat of T Cell Survival and a Potential Therapeutic for Inflammatory Diseases and Cancers
Source: Front Immunol. 2022 Aug 5;13:918241. doi: 10.3389/fimmu.2022.918241 (PMC9389583; doi:10.3389/fimmu.2022.918241)
Supplement: Supplementary file 2 [file DataSheet_2.zip › Supplemental Figures 8-10.pdf]

Supplementary Figure 8. DMGV suppresses arthritic inflammation in SKG mice.

A. DMGV inhibits survival of dividing T cells. DMGV (5µM) was added to WT or AGXT2-KO T cells 24h or 48h after stimulation and survival rates were measured by flow cytometry. **B. C.** DMGV inhibits cell division *in vivo*. Total mouse splenic T cells from C57BL/6 mice ($3x10^6$/mouse) were adoptively transferred to syngeneic SCID mice (N=2). After 2 weeks, the mice were injected i.p. with PBS or DMGV (1mg/mouse) 2 times in a 3-day interval. On day 7, mice were injected i.p. with EdU (5-ethynyl-2'-deoxyuridine) and labeled for 2 hours. The frozen sections of the spleens and colons were stained with Click-iT® EdU kit according to the manufacturer's instruction. The number of EdU^+ and/or $CD3^+$ cells in the entire spleen or colon section was counted using ImageJ software. **D.** DMGV prevents progression of arthritis in SKG mice. SKG mice with moderate arthritis (AS 4-5) were injected with either PBS or DMGV (750µg/mouse) i.p. as indicated and the ankle swelling was measured for 2 weeks. **E.** Histological analysis of the hind ankles of control and diseased SKG mice (arthritis score 8, AS8). The hind legs were processed and stained for H&E as described [2]. For immunofluorescence, antigen retrieval in citrate buffer (pH 6.0) was performed by heating the tissues in a 100°C water bath for 15min, followed by staining with anti-mouse Il-17A-Alexa fluor 488 and anti-mouse CD3-Alex fluor 594 (1:200 dilution in PBS-0.1%Triton X-100) and confocal imaging. **F.** Arthritis score (AS), H&E staining, and $IL\text{-}17A^+$ T cell staining of individual mouse.

Supplementary Figure 8

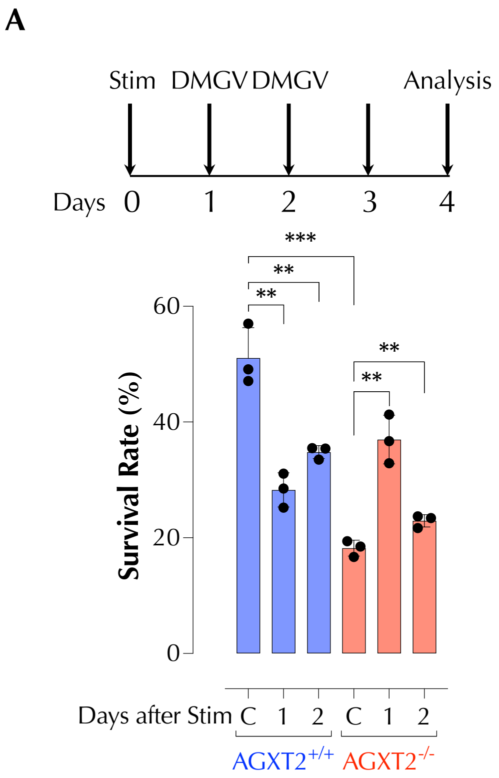

Supplementary Figure 8

B (Spleen)

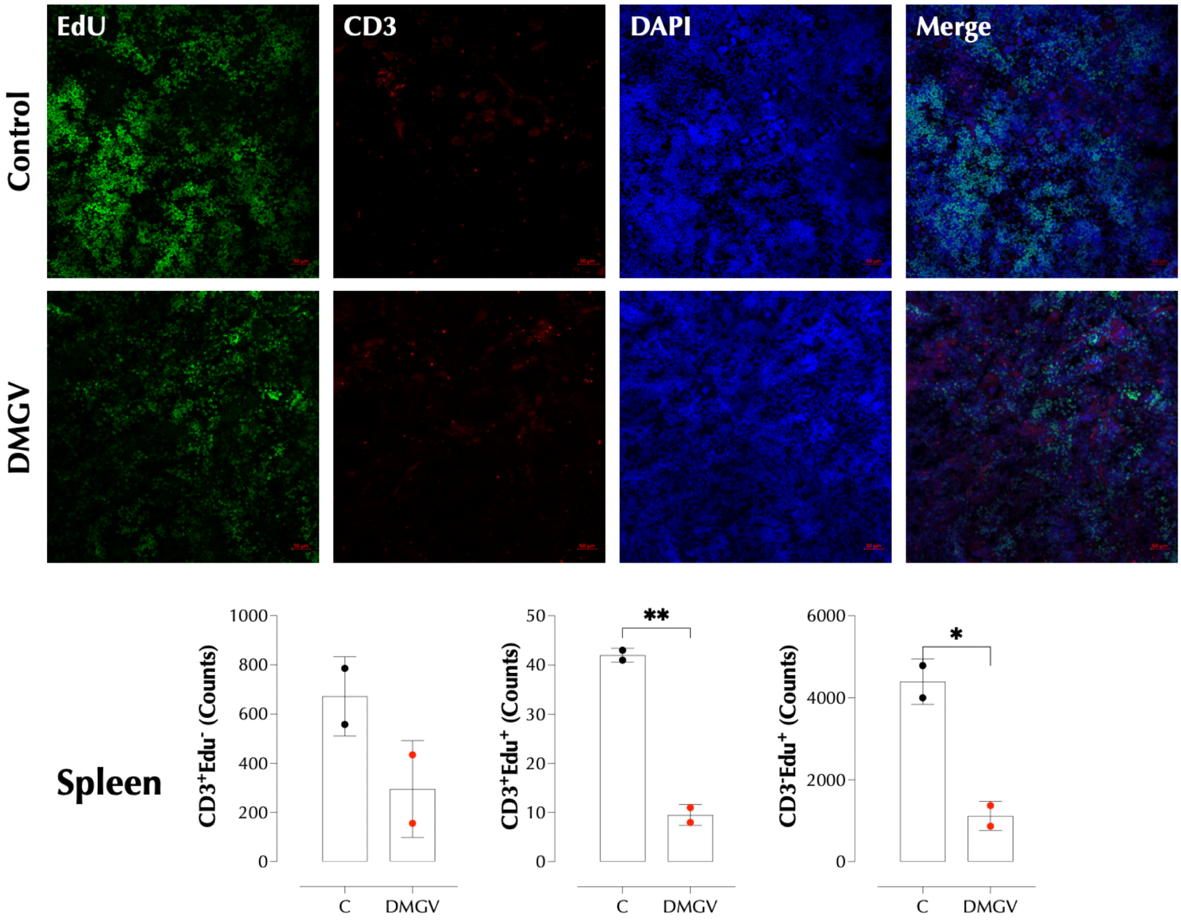

Supplementary Figure 8

C (Colon)

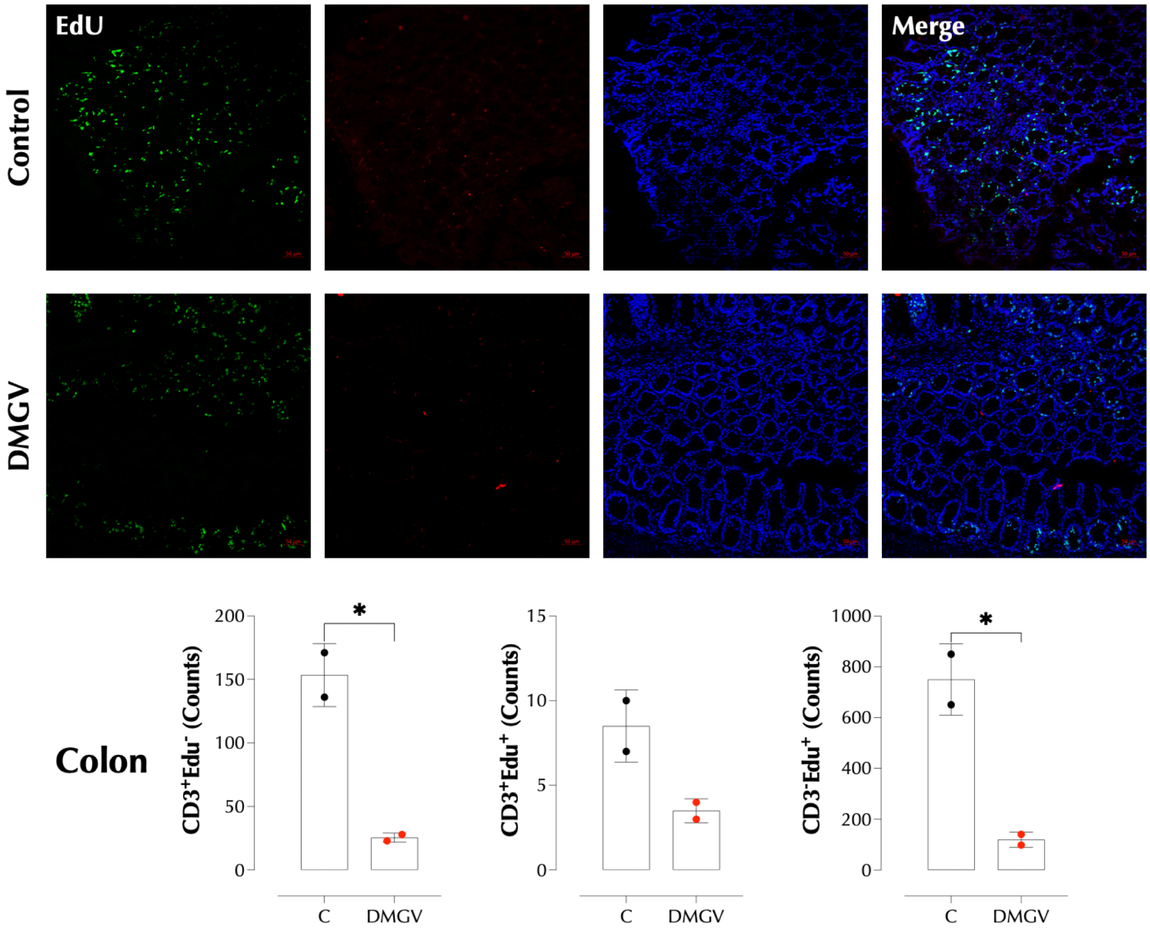

Supplementary Figure 8

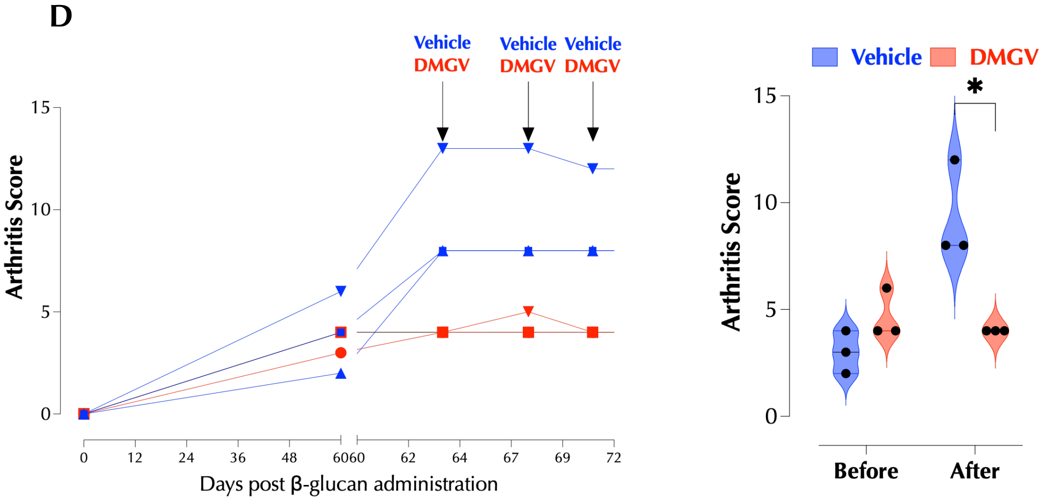

Supplementary Figure 8

E

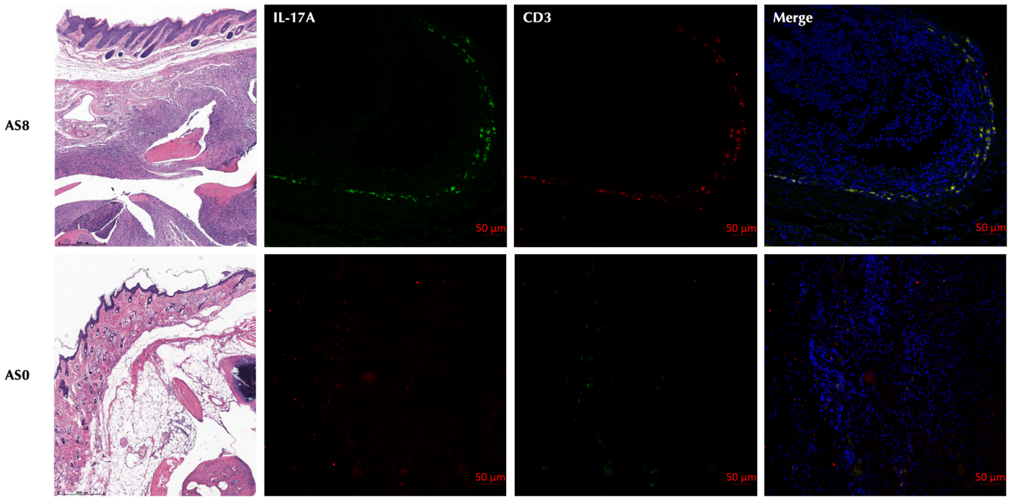

F

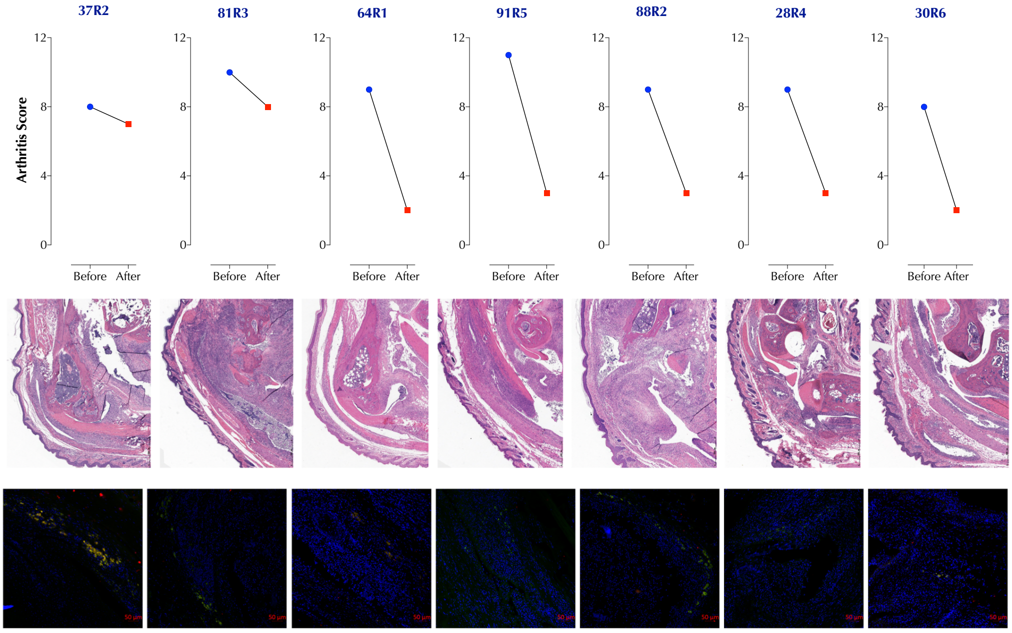

Supplemental Figure 9

A. DMGV induces cell death in cancer cell lines. The indicated cell lines were treated with an increasing amount of DMGV and the survival rate was measured by flow cytometry after 72 hours. The data are the representatives of at least 2 separate experiments. HCT-8 cells were cultured in 2D or 3D Matrigel and survival was measured by CCK-8 as described in the method. **B.** DMGV induces cell death in blood cancer cell lines by the same mechanism that inhibits survival and expansion of normal T cells. Jurat T cells were treated with DMGV with or without NAC (1mM), Ru360 (1µM), CGP (1µM), pyruvate (1mM), βNMN (15µM), or TGFβ (15ng/ml). The survival rate was measured by flow cytometry after 72 hours. **C.** Establishment of the Jurkat leukemia mouse model. NOD/SCID mice were injected with Jurkat T cells ($1x10^7$/mouse in 1ml of PBS) i.v. and the cellularity in the peripheral blood was measured after 2 weeks by flow cytometry. When the Jurkat cells reach above 50% of the blood cells as shown in the right, mice were divided randomly and treated with DMGV. Jurkat and uninjected NOD/SCID mouse were used as the controls.

Supplementary Figure 9

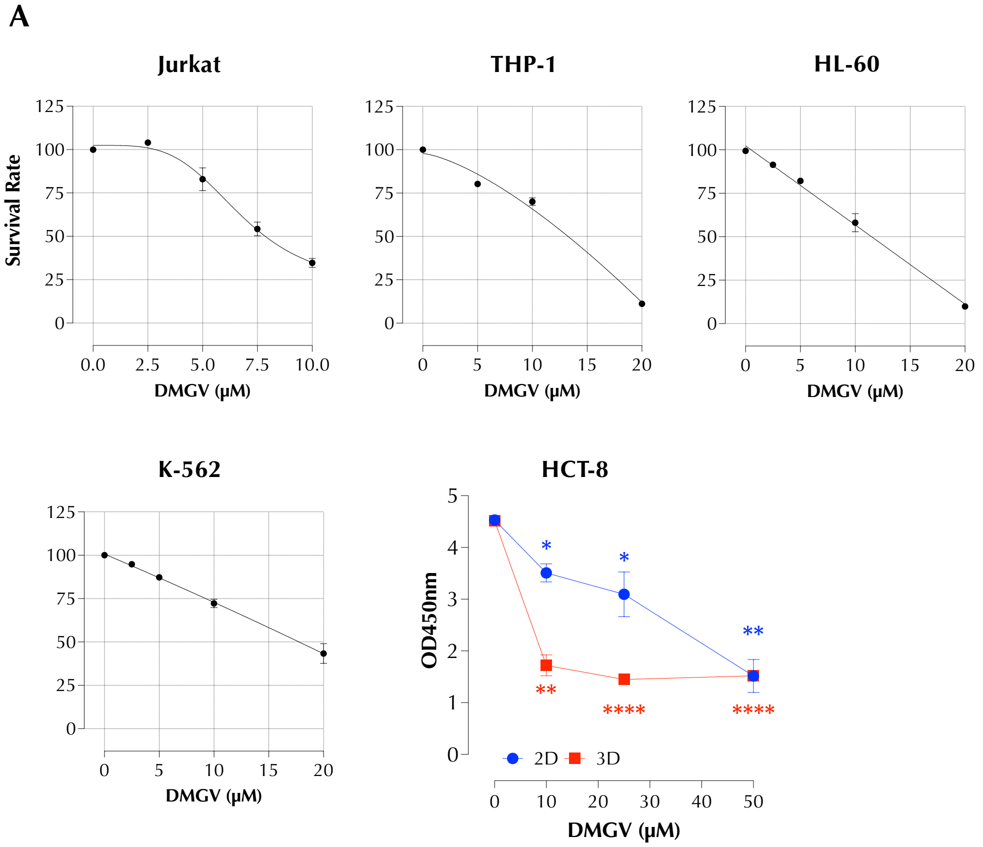

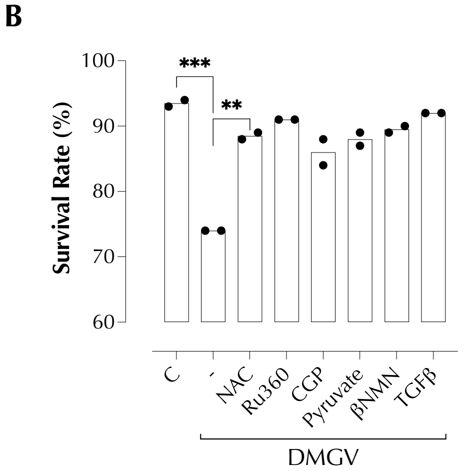

Supplementary Figure 9

C

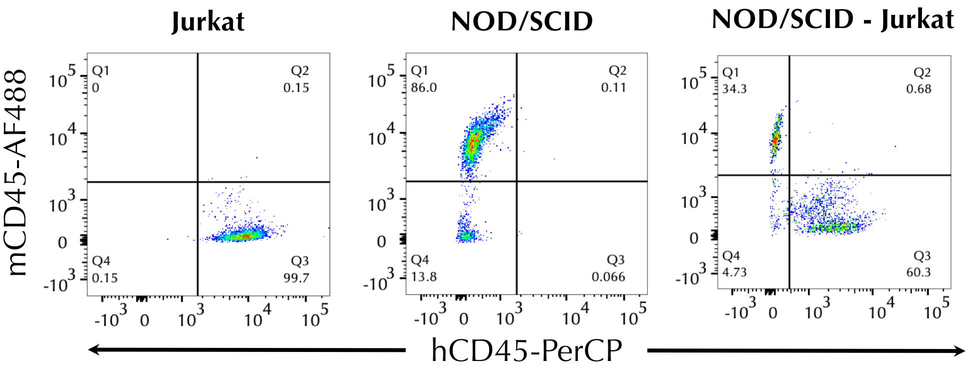

Supplemental Figure 10. DMGV is orally available. We tested bioavailability of oral administration of DMGV in SD rats as described in the method section.

Top: the plasma DMGV level after I.P. injection reached the peak at 0.25h and the half-life was 3.31h. **Middle**: the plasma DMGV level after P.O. reached the peak at 0.833h and the half-life was 6.99h. **Bottom:** the relative bioavailability [F_{Rel}] = P.O./I.P was 15%.

Supplementary Figure 10

Top

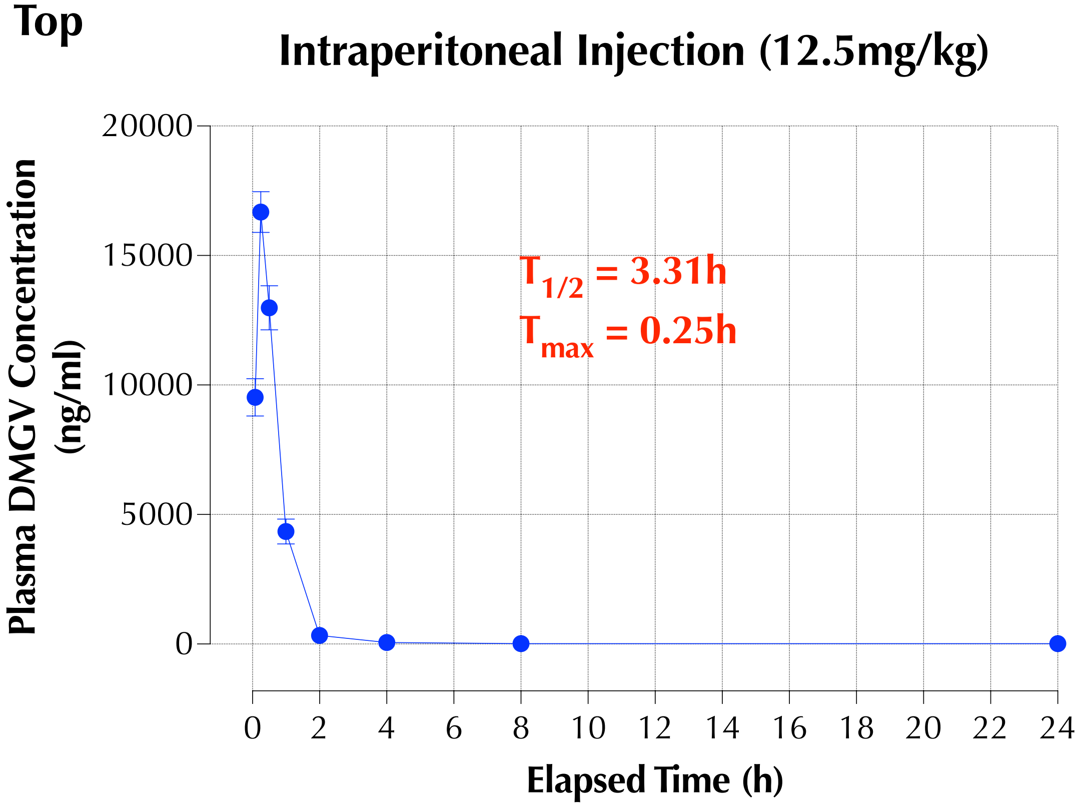

Middle

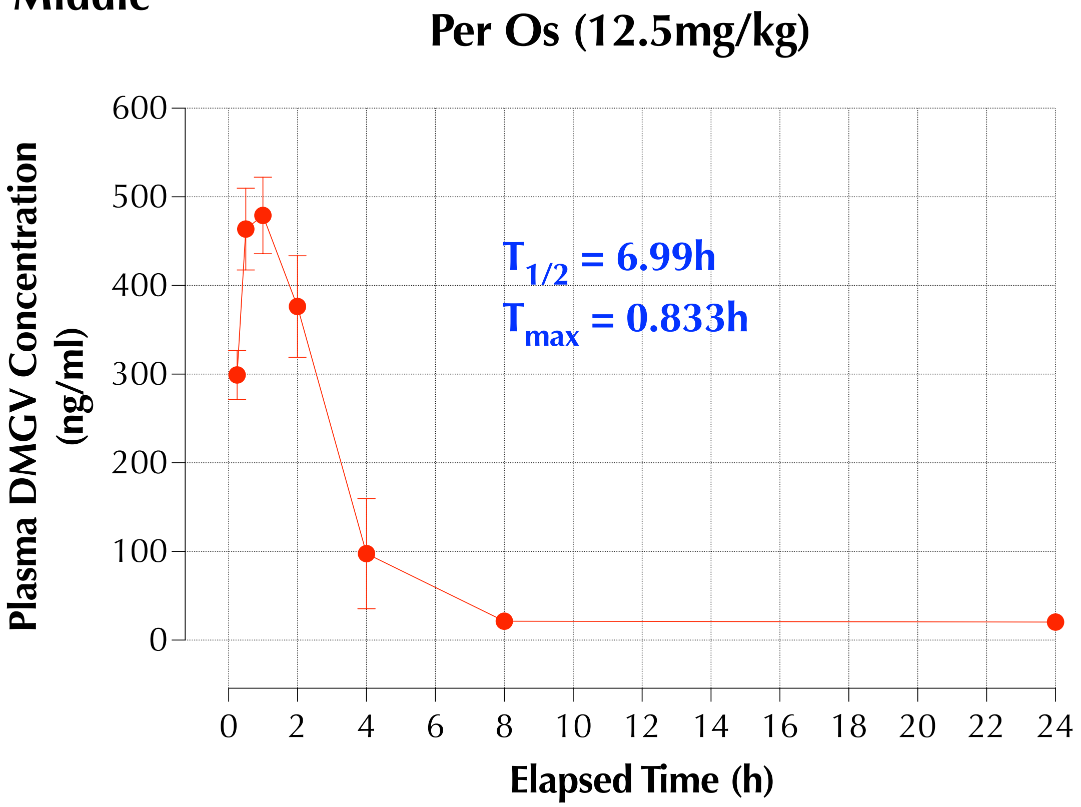

Supplementary Figure 10

Bottom

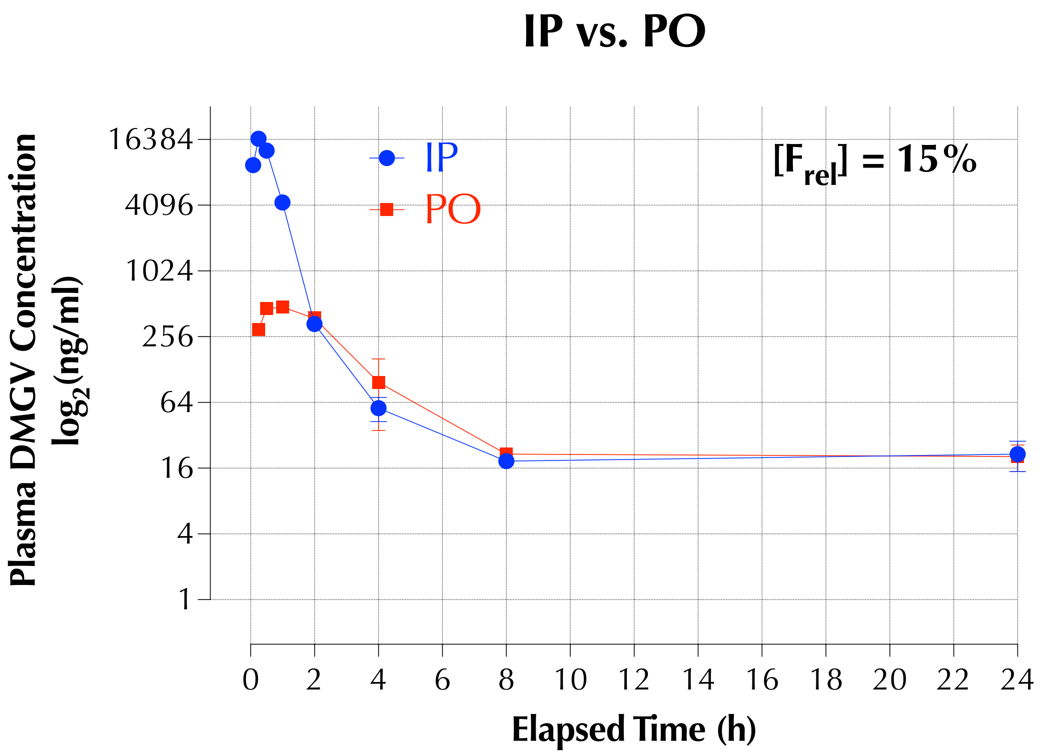

Reference

[1] P. Hernansanz-Agustin, C. Choya-Foces, S. Carregal-Romero, E. Ramos, T. Oliva, T. Villa-Pina, L. Moreno, A. Izquierdo-Alvarez, J.D. Cabrera-Garcia, A. Cortes, A.V. Lechuga-Vieco, P. Jadiya, E. Navarro, E. Parada, A. Palomino-Antolin, D. Tello, R. Acin-Perez, J.C. Rodriguez-Aguilera, P. Navas, A. Cogolludo, I. Lopez-Montero, A. Martinez-Del-Pozo, J. Egea, M.G. Lopez, J.W. Elrod, J. Ruiz-Cabello, A. Bogdanova, J.A. Enriquez, and A. Martinez-Ruiz, Na(+) controls hypoxic signalling by the mitochondrial respiratory chain. Nature 586 (2020) 287-291.

[2] M. Hashimoto, K. Hirota, H. Yoshitomi, S. Maeda, S. Teradaira, S. Akizuki, P. Prieto-Martin, T. Nomura, N. Sakaguchi, J. Kohl, B. Heyman, M. Takahashi, T. Fujita, T. Mimori, and S. Sakaguchi, Complement drives Th17 cell differentiation and triggers autoimmune arthritis. J Exp Med 207 (2010) 1135-43.
